# Axial Ligand Tuning of the Catalytic Two- versus Four-Electron Reduction of Dioxygen by a Dinuclear Cobalt Complex

**DOI:** 10.1021/jacs.6c12922

**Published:** 2026-09-30

**Authors:** Ayan Bera, Xinyang Peng, Dibya Jyoti Barman, Michael Haumann, Sagie Katz, Dennis G.H. Hetterscheid, Peter Hildebrandt, Kallol Ray

**Affiliations:** † Department of Chemistry, 9373 Humboldt-Universität zu Berlin, Brook-Taylor-Straße 2, Berlin 12489, Germany; ‡ 9166 XAS@KMC-3, BESSY-II+, Helmholtz Zentrum Berlin fur Materialien und Energie GmbH, Albert-Einstein-Straße 15, Berlin 12489, Germany; § Department of Chemistry, 26524 Technische Universität Berlin, Berlin 10623, Germany; ∥ Leiden Institute of Chemistry, 4496 Leiden University, P.O. Box 9502, Leiden 2300 RA, The Netherlands

## Abstract

Controlling the two- versus four-electron oxygen reduction reaction (ORR) remains a central challenge, as the factors that determine whether peroxide is released as H_2_O_2_ or further reduced to H_2_O are often difficult to define at the molecular level. Here we show that axial ligation provides a direct tool for switching ORR selectivity in a flexible dicobalt platform, Co_2_L^1,3^ (where L^1,3^ = 1,3-bis­[(bis­(2,2′-bipyridin-6-yl)­amino)­methyl]­benzene). In acetonitrile (MeCN), the solvent-bound complex mediates a mixed 2e^–^/4e^–^ ORR, whereas the presence of additional ligands like triphenylphosphine (PPh_3_) or 2,6-dicyclohexylimidazole (DCHIm) biases the reaction toward sole H_2_O_2_ formation. An anionic thiophenolato coordination, in contrast, redirects the reaction toward a near-quantitative 4e^–^/4H^+^ reduction to water. Kinetic studies reveal distinct empirical rate laws, indicating a ligand-dependent change in the turnover-limiting sequence. A dicobalt­(III)–peroxido intermediate has been identified as the key branching point by spectroscopic and structural characterization of the O_2_-derived intermediate. Neutral axial ligands (MeCN, PPh_3_ or DCHIm) stabilize this intermediate against O–O bond cleavage, thereby leading to the release of H_2_O_2_ upon protonation. In contrast, anionic thiophenolato binding promotes irreversible O–O bond cleavage to form a structurally characterized dicobalt­(III) bis-μ-oxido moiety, with the concomitant oxidation of thiophenolato to phenylthiyl radical and the eventual release of water in the presence of a proton donor. These results establish an axial-ligand-controlled mechanism for tuning ORR selectivity. In particular, the thiophenolate-induced cobalt-mediated O–O bond cleavage represents a bioinspired model of the proposed role of cysteine-assisted O–O bond cleavage in cytochrome P450 enzymes.

## Graphical abstract

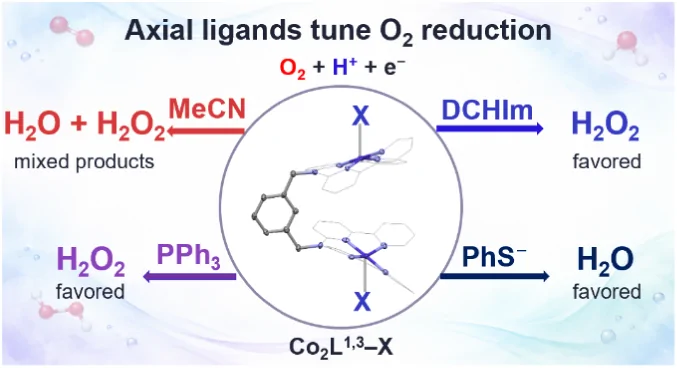

## Introduction

The oxygen reduction reaction (ORR) is an essential process in biological energy metabolism and is also a vital reaction involved in many new artificial energy conversion techniques, including fuel cells.^1^ In protic media, O_2_ reduction can proceed either by a two-electron/two-proton pathway to H_2_O_2_ or by a four-electron/four-proton pathway to water, and the ability to steer this selectivity with earth-abundant transition-metal catalysts is central to efficient energy utilization and decentralized oxidant production.^1b,2^ Molecular catalysts offer a particularly incisive platform in this context. Their well-defined coordination environments enable quantification of the thermodynamics of individual proton- and electron-transfer steps, as well as the stability and reactivity of the reactive oxygen intermediates, with a level of detail that is rarely accessible for heterogeneous materials.^1b,3^

A rich body of work with iron,^4^ cobalt,^5^ manganese^4g,6^ and copper^7^ complexes has mapped out how metal identity, ligand electronics, and the secondary coordination sphere influence both the rate and selectivity of the ORR.^3a,5f,8^ Strategies that have emerged include installing pendant proton relays^8b,9^ and hydrogen-bond donors^8a,9a,9b,10^ , incorporating Lewis acids,^11^ and constructing dinuclear sites^12^ that favor cooperative O–O bond activation. In cobalt chemistry, macrocyclic N_4_ ligands and, more recently, N_2_O_2_ or N_2_S_2_ scaffolds have delivered catalysts that are highly selective often for the two-electron reduction of O_2_ to H_2_O_2_.^5d,5i,8b^ However, this selectivity is not universal, as related N_2_S_2_-supported cobalt complexes can also promote the four-electron reduction of O_2_ to H_2_O.^5e^ These reactions often proceed via Co–superoxido or Co–hydroperoxido resting states, in which a delicate balance between O–O bond retention and protonation steps is shown to control product release.^1a,5a,5d–5f,5h,8b,13^ Complementary studies on multinuclear architectures have shown that subtle changes in the framework or reaction conditions can bias peroxido intermediates either toward protonation to H_2_O_2_ or toward O–O bond cleavage and water formation.

For example, a hexanuclear cobalt stannoxane complex switches from 4e^–^/4H^+^ to 2e^–^/2H^+^ reduction simply upon raising the temperature, highlighting how small perturbations in the stability of a μ-1,2-peroxidodiocobalt­(III) unit can invert global selectivity.^5g^ More broadly, ORR selectivity can be controlled by the mode of electron delivery. For example, a non-heme diiron thiolate complex was reported to produce H_2_O_2_ selectively under chemical reduction conditions, while predominantly forming H_2_O during electrocatalysis.^14^

In previous studies cofacial dicobalt systems including pillared cobalt bisporphyrins,^12a,15^ porphyrin–corrole dyads,^16^ Schiff-base calixpyrrole Pacman complexes,^17^ and cobalt porphyrin prisms^18^ have been employed for ORR (Table S9), and they all perform a predominant 4e^–^/4H^+^ reduction to H_2_O. Herein, we report the synthesis and characterization of a flexible dicobalt complex, **Co**_**2**_ **L** ^**1,3**^, involving a non-porphyrin ligand system, and its unprecedented ability to change the number of electrons in the catalytic reduction of O_2_ from two to four by simply changing the axial ligation. The reasons for the catalyst’s ability to act in either the two- or four-electron reduction of O_2_ in the presence of decamethylferrocene (Fc*) as a one-electron reductant and trifluoroacetic acid (TFA) as a proton source are elucidated on the basis of detailed kinetic studies of the overall catalytic reactions, as well as individual catalytic steps and the characterization of dicobalt­(III)–peroxido intermediates, which are presumably formed during the catalytic cycle. The mechanistic insights obtained in this study may serve as useful and broadly applicable principles for the future design of more efficient and selective catalysts for the activation of dioxygen.

## Results and Discussion

### Synthesis and Characterization of L^1,3^ and Co_2_L^1,3^

The ligand L^1,3^ (Scheme 1, inset) was synthesized via a S_N_^2^ reaction involving the previously reported Hbbpya (where Hbbpya = N,N-bis­(2,2′-bipyrid-6-yl)­amine)^19^ ligand and α,α′-dibromo-meta-xylene (see SI for details and Figure S1 for characterization). The complex **Co**_**2**_ **L** ^**1,3**^ **(OTf)** was achieved by combining L^1,3^ and two equiv of Co­(OTf)_2_ in MeCN or, DMF at room temperature (see SI for detailed synthesis and characterization). The corresponding **Co**_**2**_ **L** ^**1,3**^ **(BF**_**4**_) complex was also synthesized by using Co­(BF_4_)_2_ as the metal precursor. Single-crystal X-ray diffraction (scXRD) studies highlight the highly flexible nature of the bbpya-based dicobalt scaffold (Scheme 1). In presence of the weakly coordinating and bulky OTf ^–^ anion, the two cobalt centers are held far apart and do not interact directly: the complex adopts an extended conformation with a Co···Co distance of 14.032(1) Å, in which each Co is bound to an individual bbpya arm and the aromatic bridge remains essentially linear (Scheme 1a). In sharp contrast, crystals obtained in the presence of a BF_4_^–^ anion reveal a folded, cofacial arrangement of the two bbpya units. In this structure a BF_4_^–^ anion is trapped in the ligand cleft, drawing the metal ions into close proximity with a Co···Co separation of 6.673(1) Å (Scheme 1b). The complex **Co**_**2**_ **L** ^**1,3**^ **(OTf)** was further characterized by ^1^H-NMR, ESI-MS, and EPR spectroscopy (Figures S2–S3). The ^1^H-NMR spectrum of **Co**_**2**_ **L** ^**1,3**^ **(OTf)** in MeCN-*d*_3_ revealed ligand resonances in the paramagnetic region. X-band EPR spectrum at 13 K exhibited an axial EPR spectrum (Figure S3a), in which the hyperfine splitting due to ^59^Co (*I* = 7/2) is resolved along the z direction, resulting in the *g*_*z*_ component splitting into eight lines. Simulations of the spectra unveiled optimized *g* tensors with principal components [*g*_*x*_, *g*_*y*_, *g*_*z*_] = [2.279, 2.216, 2.023] indicative of a low spin (*S* = 1/2) Co­(II) configuration with a $d_{z^{2}}$ ground state (Figure S3a). The cobalt hyperfine coupling constants were determined to be [*A*_*x*_, *A*_*y*_, *A*_*z*_] = [33, 53, 264] MHz. In addition, super-hyperfine coupling due to two equivalent ^14^N (*I* = 1) nuclei along the z direction (∼33 MHz) could also be observed.

**Scheme 1:**
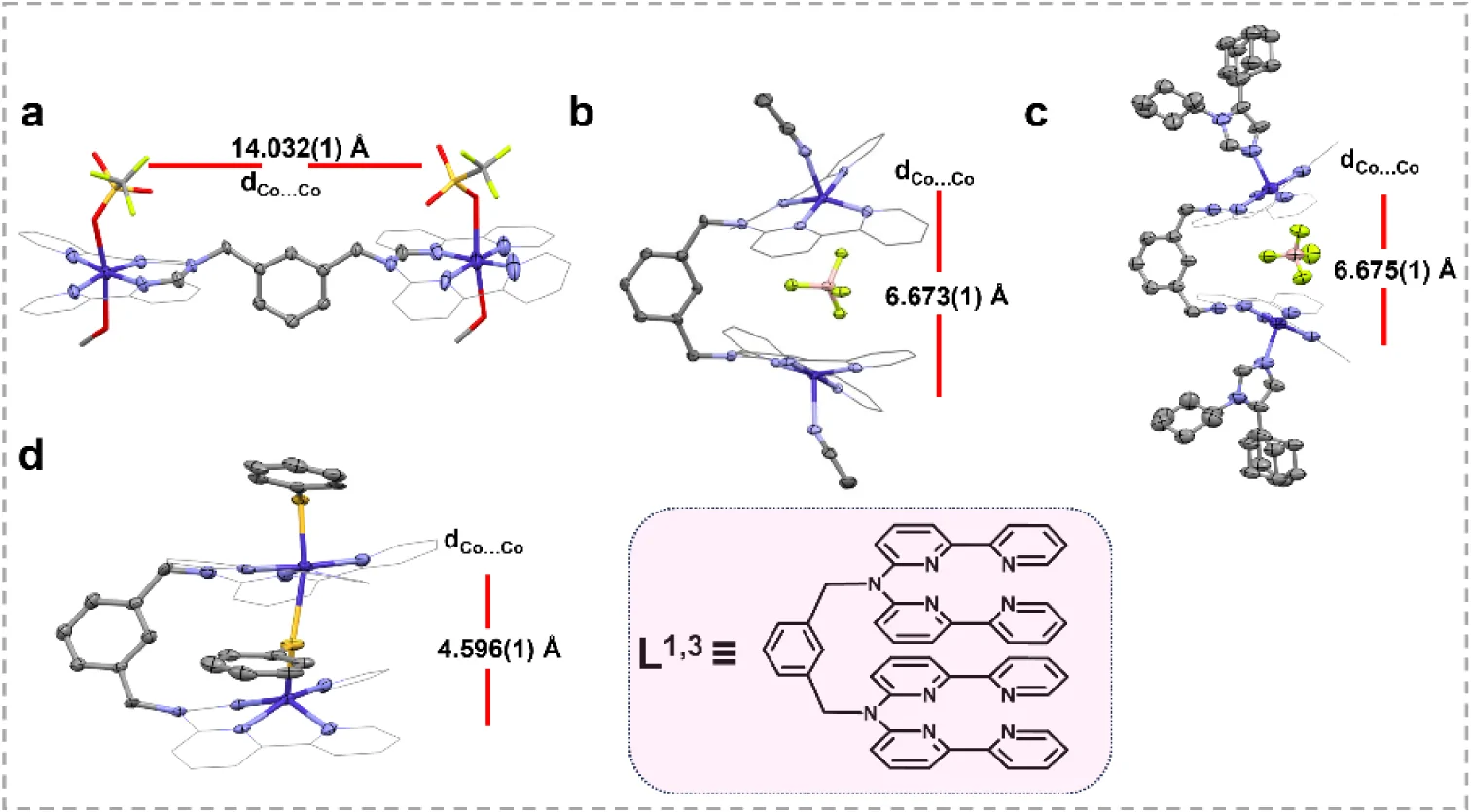
Molecular Structures of (a) **Co_2_L^1,3^(OTf)**, (b) **Co_2_L^1,3^(BF_4_)**, (c) **Co_2_L^1,3^-DCHIm**, and (d) **Co_2_L^1,3^-SPh** with Ellipsoids Shown at 50% Probability^a^

a Non-coordinating anions and hydrogen atoms are omitted for clarity. The chemical structure of L^1,3^ is shown as an inset.

The solution speciation of the dicobalt­(II) complex, **Co**_**2**_ **L** ^**1,3**^ **(OTf)** was then examined by UV–vis spectroscopy in the presence of different axial ligands (Figure S4). Dissolution of the **Co**_**2**_ **L** ^**1,3**^ **(OTf)** complex in MeCN gives the solvent-bound species **Co**_**2**_ **L** ^**1,3**^ **-MeCN**, which shows absorption bands at 421 (*ε* = 2250 M^–1^ cm^–1^), and 455 (*ε* = 2300 M^–1^ cm^–1^) nm (Figure S4a). Addition of PPh_3_ leads to new absorptions at 500 (*ε* = 1200 M^–1^ cm^–1^), and 553 (*ε* = 800 M^–1^ cm^–1^) nm, consistent with formation of the phosphine-bound complex, **Co**_**2**_ **L** ^**1,3**^ **-PPh**_**3**_ (Figure S4b). **Co**_**2**_ **L** ^**1,3**^ **-PPh**_**3**_ displays rhombic *S* = 1/2 signals with *g* = [2.291, 2.147, 1.975]. It shows *A*(^59^Co) = [46, 64, 213] MHz together with pronounced ^31^P superhyperfine coupling, *A*(^31^P) = [219, 211, 8] MHz, supporting retention of the coordinated phosphine in frozen solution. The spectrum is compatible with two magnetically equivalent, weakly interacting low-spin Co­(II) centers (Figure S3b). In contrast, addition of 2,6-dicyclohexylimidazole (DCHIm) attenuates the parent MeCN-bound features without generating a new strongly allowed visible transition, consistent with the formation of **Co**_**2**_ **L** ^**1,3**^ **-DCHIm** (Figure S4c), which is independently authenticated by scXRD (Scheme 1c). The crystal structure reveals five-coordinate, distorted square-pyramidal Co­(II) centers with axially coordinated DCHIm. Notably, the displacement of each Co center from the mean equatorial N_4_-donor plane increases from 0.18 Å in **Co**_**2**_ **L** ^**1,3**^ **-MeCN** to 0.54 Å in **Co**_**2**_ **L** ^**1,3**^ **-DCHIm** (Figure S5). Interestingly, in contrast to the *S* = 1/2 ground-states of **Co**_**2**_ **L** ^**1,3**^ **-MeCN** and **Co**_**2**_ **L** ^**1,3**^ **-PPh**_**3**_, **Co**_**2**_ **L** ^**1,3**^ **-DCHIm** exhibits a broad and strongly anisotropic high-spin Co­(II) signal that is reproduced with a full *S* = 3/2 Hamiltonian using intrinsic *g* = [2.62, 2.18, 2.12], |D| = 53.3 GHz, and |E| = 6.20 GHz (|E/D| = 0.116) (Figure S3c). The corresponding lower-Kramers-doublet effective *g* values are [5.06, 4.29, 2.04]. The spin state change in **Co**_**2**_ **L** ^**1,3**^ **-DCHIm** is attributed to the strong axial sigma donation and steric demand of DCHIm, which likely promote the observed out-of-plane distortion of Co that stabilizes the $d_{x^{2}-y^{2}}$ orbital, thereby, resulting in the *S* = 3/2 ground state. Notably, closely related geometry-dependent low-spin/high-spin changes have been reported for five-coordinate imidazole- and pyridyl-ligated Co­(II) Schiff-base and porphyrin complexes.^20^

The most pronounced spectral change is observed upon addition of NaSPh, which gives an intense band at 462 nm (*ε* = 4650 M^–1^ cm^–1^) and a weak shoulder at 764 nm (*ε* = 300 M^–1^ cm^–1^) (Figure S4d). The high intensity of the 462 nm band is consistent with coordination of an anionic thiophenolato ligand in **Co**_**2**_ **L** ^**1,3**^ **-SPh** and likely reflects enhanced sulfur-to-cobalt charge-transfer character.^21^ The scXRD of **Co**_**2**_ **L** ^**1,3**^ **-SPh** shows one thiophenolate ligand bridging the two cobalt centers through sulfur, while a second thiophenolate ligand is bound terminally in an axial position to one cobalt site (Scheme 1d). Notably, the phenyl group of PhS^–^ is involved in strong π-stacking interaction with the bbpya ligand moieties of **L** ^**1,3**^ (Figure S4e). This asymmetric bridging/terminal binding mode demonstrates that thiophenolate does not simply replace MeCN as a neutral axial donor, but also reorganizes the dicobalt core through strong Co–S interactions. **Co**_**2**_ **L** ^**1,3**^ **-SPh** is EPR silent consistent with antiferromagnetic coupling between the thiophenolato-bridged Co­(II) centers (Figure S3d). Together, these ligand-dependent spectral and structural changes establish formation of the axial-ligand series, **Co**_**2**_ **L** ^**1,3**^ **-X** (where X = MeCN, PPh_3_, DCHIm, and PhS^–^) and show that the electronic structure of the dicobalt­(II) core is strongly modulated by the axial donor.

### Homogeneous Catalytic ORR Studies

Catalytic ORR studies by **Co**_**2**_ **L** ^**1,3**^ bearing different axial ligands, **Co**_**2**_ **L** ^**1,3**^ **-X** (where X = MeCN, PPh_3_, DCHIm, and PhS^–^) were carried out in MeCN at 298 K with Fc* as the reductant and TFA as the proton source. The reduction of O_2_ was monitored by the formation of the decamethylferrocenium cation (Fc*^+^) at 778 nm by UV–vis spectroscopy (Figure 1). Under catalytic conditions, an air-saturated MeCN solution of **Co**_**2**_ **L** ^**1,3**^ **-X** (10 μM) was treated with excess Fc* (1 mM), and TFA (10 mM). In all cases, the addition of O_2_ led to the rapid appearance of the Fc*^+^ band (Figure S6), whereas only a very small background signal was observed in the absence of the cobalt complex under otherwise identical conditions (Figure 1e, black trace), indicating that Fc* does not reduce O_2_ at an appreciable rate without the catalyst.

**Figure 1:**
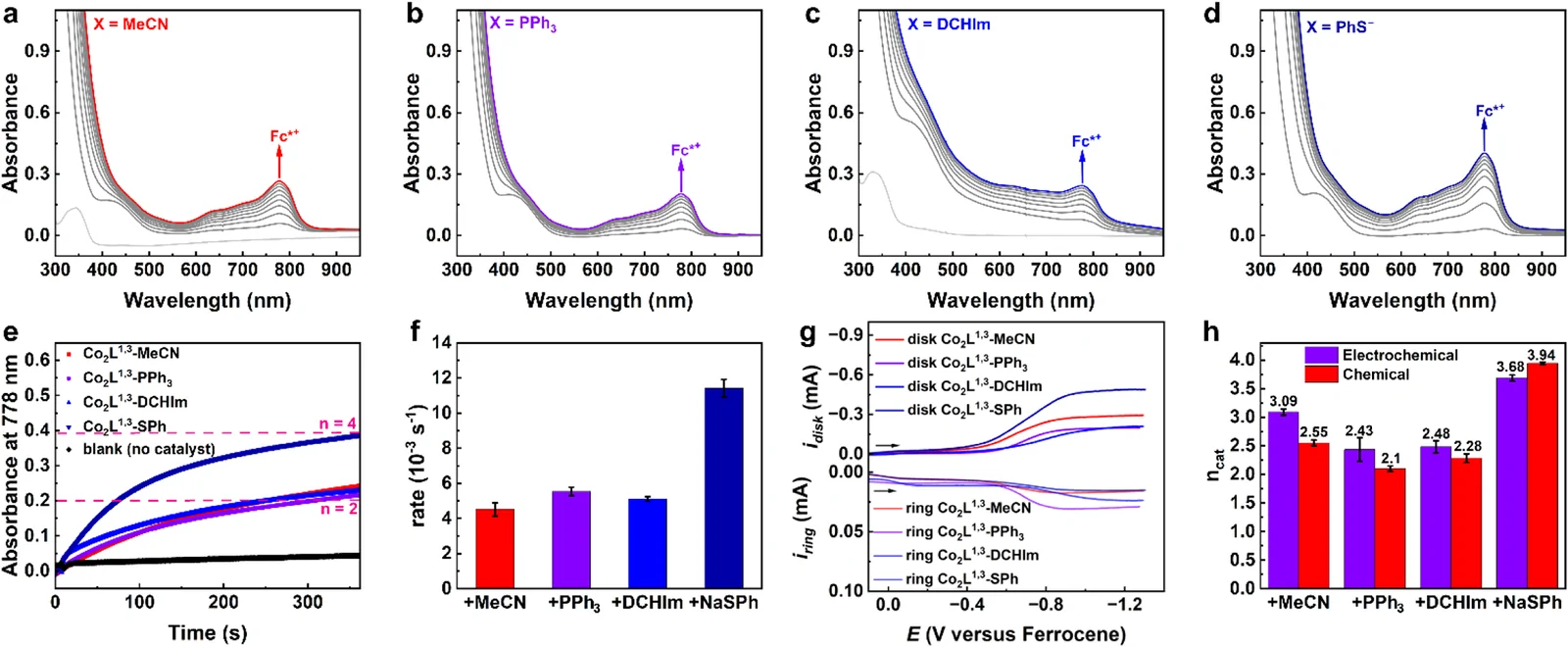
UV–vis spectral changes associated with the catalytic ORR mediated by **Co_2_L^1,3^-X** complexes in MeCN at 298 K under limiting-O_2_ conditions ([O_2_]_0_ = 0.2 mM; [Co_2_L^1,3^-X] = 10 μM; [Fc*] = 1 mM; [TFA] = 10 mM): (a) X = MeCN, (b) X = PPh_3_, (c) X = DCHIm, (d) X = PhS^–^. (e) Time profiles of the Fc*^+^ formation during ORR by **Co_2_L^1,3^-X** complexes. (f) Observed ORR rates of **Co_2_L^1,3^-X** complexes. (g) RRDE measurements performed in O_2_-saturated MeCN ([O_2_] = 8.1 mM; rotation rate = 1600 rpm). (h) Number of electrons transferred per O_2_ molecule (n_cat_), determined from chemical ORR under limiting-O_2_ conditions ([O_2_]_0_ = 0.2 mM) and from RRDE measurements under O_2_-saturated conditions ([O_2_] = 8.1 mM). Values in panels f and h represent average values obtained from three independent experiments, and the error bars represent ± standard deviation (n = 3).

To determine the selectivity of ORR mediated by **Co**_**2**_ **L** ^**1,3**^ **-X**, we also checked the ORR reactivity in the presence of limiting amount of O_2_ (0.2 mM) keeping other conditions fixed (Figure 1). Under these conditions, Fc* is present in large excess relative to O_2_, the overall number of electrons transferred per O_2_ (n_cat_) can be obtained directly from the final concentration of Fc*^+^ according to n_cat_ = [Fc*^+^]_(final)_/[O_2_]_(initial)_. In this system, reduction of O_2_ by 2e^–^/2H^+^ to H_2_O_2_ will correspond to n_cat_ = 2, whereas a full 4e^–^/4H^+^ reduction to 2 H_2_O warrants n_cat_ = 4. For **Co**_**2**_ **L** ^**1,3**^ **-MeCN**, the reaction yields [Fc*^+^]_(final)_ ≈ 0.51 mM from 0.2 mM O_2_, giving n_cat_ = 2.55 (Figure 1a, h). This value indicates a mixture of 2e^–^ and 4e^–^ pathways, corresponding to ca. 72.5% 2e^–^ reduction to H_2_O_2_ and 27.5% 4e^–^ reduction to water (see pages S6–S7 of the SI for calculation details). Independent quantification of H_2_O_2_ by iodometric titration gave 68% H_2_O_2_, in good agreement with the stoichiometric analysis (Figure S7). The stability of **Co**_**2**_ **L** ^**1,3**^ **-MeCN** (10 μM), under catalytic condition was verified. For this purpose, 1 mM fresh Fc* were introduced at 5, 285, and 500 s (Figure S8). In each case, we observed a quasi-quantitative Fc*^+^ production with the same rate, establishing the stability of **Co**_**2**_ **L** ^**1,3**^ **-MeCN** under the conditions employed.^22^

In the presence of PPh_3_ or DCHIm, the **Co**_**2**_ **L** ^**1,3**^ **-X** complexes (X = PPh_3_, DCHIm) display n_cat_ values of 2.10 and 2.28, respectively (Figure 1b,c), which correspond to more quantitative formation of H_2_O_2_. Colorimetric assays further confirmed H_2_O_2_ as the major product in both cases (94% for **Co**_**2**_ **L** ^**1,3**^ **-PPh**_**3**_ and 88% for **Co**_**2**_ **L** ^**1,3**^ **-DCHIm**) (Figures S9–S10). Interestingly, when PhS^–^ was used as an axial ligand the final concentration of generated Fc*^+^ was found to be 3.94 times that of the O_2_ concentration (0.2 mM), giving n_cat_ = 3.94 (Figure 1d, h). Analysis of the reaction mixture by iodometric titration revealed no formation of H_2_O_2_ as the oxygen-derived product (Figure S11). Thus, the reaction of **Co**_**2**_ **L** ^**1,3**^ **-SPh** with O_2_ in the presence of a chemical reductant is selective for 4e^–^/4H^+^ reduction.

Amongst all the complexes, **Co**_**2**_ **L** ^**1,3**^ **-SPh** shows the highest rate for O_2_ reduction with a *k*_obs_ value of 1.2 × 10^–2^ s^–1^ (Figure 1f), which is approximately 2.1–2.6 times faster as compared to that of the **Co**_**2**_ **L** ^**1,3**^ **-X** (X = MeCN (4.5 × 10^–3^ s^–1^), PPh_3_ (5.5 × 10^–3^ s^–1^), or DCHIm (5.1 × 10^–3^ s^–1^)) complexes.

Next, we investigated electrochemical ORR activity of the **Co**_**2**_ **L** ^**1,3**^ **-X** complexes in MeCN at 298 K. **Co**_**2**_ **L** ^**1,3**^ **-MeCN** revealed a redox feature at *E*_1/2_ = 0.58 V vs. Fc^+^/Fc, which can be assigned as the Co^III/II^ redox couple (Table S10; Figure S12a). For **Co**_**2**_ **L** ^**1,3**^ **-X** complexes this peak is shifted cathodically; a trend of increasing cathodic shift with increasing σ-donation in the order PhS^–^ (−0.26 V) > DCHIm (−0.16 V) > PPh_3_ (0.30 V) > MeCN (0.58 V) is observed (Figure S12, Table S10). Under an O_2_-saturated atmosphere in the presence of TFA (20 equiv), the emergence of significant catalytic current with onset potentials of −0.16 V (MeCN), −0.24 V (PPh_3_), −0.10 (DCHIm) and −0.16 V (PhS^–^) is noted, which is dependent on the nature of the axial ligand (Figure S14). Notably, the catalytic currents for the DCHIm and derivatives emerge close to their corresponding Co­(III)/Co­(II) processes, whereas those of the MeCN and PPh_3_ derivatives appear at substantially more negative potentials. Control experiments (Figures S13–S15) in the absence of cobalt catalysts and in the presence or absence of TFA didn’t lead to any catalytic currents originating from ORR. A rinse test confirms that the activity arises from the cobalt catalysts in solution and is not due to any electrode-adsorbed material (Figure S16). UV–vis analysis after electrolysis showed approximately 82% retention of the complex, indicating reasonable stability under the electrocatalytic conditions (Figure S17).

The axial-ligand-dependent selectivity was also examined electrochemically by rotating ring–disk electrode (RRDE) measurements (Figure 1g). The RRDE-derived electron-transfer numbers were n_cat_ = 3.09 for **Co**_**2**_ **L** ^**1,3**^ **-MeCN**, n_cat_ = 2.43 for **Co**_**2**_ **L** ^**1,3**^ **-PPh**_**3**_, n_cat_ = 2.48 for **Co**_**2**_ **L** ^**1,3**^ **-DCHIm** and n_cat_ = 3.68 for **Co**_**2**_ **L** ^**1,3**^ **-SPh** (Figure 1g, h). So, under electrochemical conditions, the same qualitative trend is retained: **Co**_**2**_ **L** ^**1,3**^ **-PPh**_**3**_ gives the most peroxido-biased response, whereas **Co**_**2**_ **L** ^**1,3**^ **-SPh** gives the most four-electron-biased response. Taken together, the data show that under both electrochemical and chemical conditions neutral P- and N-donor ligands favor the 2e^–^ ORR pathway to H_2_O_2_, whereas thiophenolato coordination promotes deeper 4e^–^ reduction to water.

### Kinetic Studies of ORR with Co_2_L^1,3^ in the Presence of Different Axial Ligands

To understand how axial ligation controls both the rate and selectivity of O_2_ reduction, we carried out kinetic studies for **Co**_**2**_ **L** ^**1,3**^ **-X** by monitoring the growth of the Fc*^+^ absorption band at 778 nm by UV–vis spectroscopy. The observed pseudo-first-order rate constants (*k*_obs_) were obtained from single-exponential fits to the time-dependent Fc*^+^ growth traces. To establish the empirical rate laws, the concentrations of **Co**_**2**_ **L** ^**1,3**^ **-X**, TFA, O_2_ and Fc* were independently varied (Figure 2). For **Co**_**2**_ **L** ^**1,3**^ **-MeCN,** *k*_obs_ showed first order dependence on [**Co**_**2**_ **L** ^**1,3**^ **-MeCN**], [TFA] and [O_2_] but no measurable dependence on [Fc*] (Figure 2a–d and Figure S19). As a result, we obtained a rate law for ORR by **Co**_**2**_ **L** ^**1,3**^ **-MeCN**, as shown in eq 1, where *k*_cat_ is the apparent third-order rate constant for the catalytic mixed 2e^–^/4e^–^ reduction of O_2_ by **Co**_**2**_ **L** ^**1,3**^ **-MeCN**.

1 $$\mathrm{rate}=\frac{d\left[{\mathrm{Fc}}^{*+}\right]}{dt}=k_{\mathrm{cat}}\left[Co_{2}L^{1,3}-MeCN\right]\left[O_{2}\right]\left[\mathrm{TFA}\right]$$

**Figure 2:**
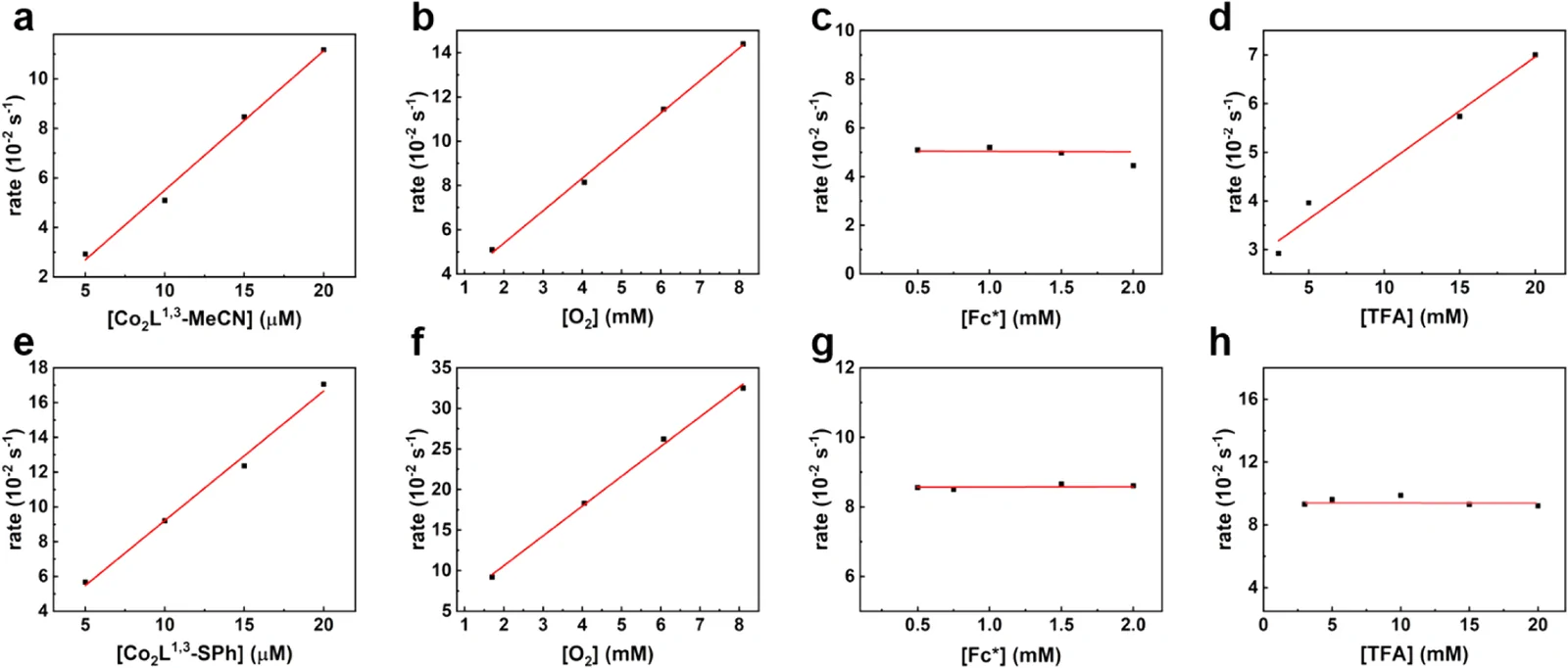
Plots of catalytic ORR rates of **Co_2_L^1,3^-MeCN** vs (a) [**Co_2_L^1,3^-MeCN**] (5–20 μM), (b) [O_2_] (1.7–8 mM), (c) [Fc*] (0.5–2 mM), and (d) [TFA] (3–20 mM). Plots of catalytic ORR rates of **Co_2_L^1,3^-SPh** vs (e) [**Co_2_L^1,3^-SPh**] (5–20 μM), (f) [O_2_] (1.7–8 mM), (g) [Fc*] (0.5–2 mM), and (h) [TFA] (3–20 mM). Typical conditions were as follows: catalyst (10 μM), O_2_ (1.7 mM), TFA (10 mM), and Fc* (1 mM).

Analogous kinetic behavior was observed for the **Co**_**2**_ **L** ^**1,3**^ **-PPh**_**3**_ and **Co**_**2**_ **L** ^**1,3**^ **-DCHIm** complexes, which showed first-order on catalyst, TFA, and O_2_ and zero-order dependence on Fc* (Figures S20–S23). The common rate law for these peroxido-selective systems suggests that proton delivery from TFA remains kinetically involved prior to, or during, the O_2_ reduction step that leads to H_2_O_2_ release.

Interestingly, for the 4e^–^/4H^+^ reduction of O_2_ by **Co**_**2**_ **L** ^**1,3**^ **-SPh**, the rate constant is proportional to the concentration of catalyst and O_2_ but showed no dependence on TFA and Fc* (Figure 2e–2h and Figure S24). Thus, we can get the following rate law as shown in eq 2, where *k*_cat_ is the apparent second-order rate constant for the catalytic 4e^–^ reduction of O_2_ by **Co**_**2**_ **L** ^**1,3**^ **-SPh**.

2 $$\mathrm{rate}=\frac{d\left[{\mathrm{Fc}}^{*+}\right]}{dt}=k_{\mathrm{cat}}\left[Co_{2}L^{1,3}-SPh\right]\left[O_{2}\right]$$

The absence of TFA and Fc* dependence on the rate of the reaction indicates that proton delivery and outer-sphere electron transfer from Fc* are not involved in the turnover-limiting step for this pathway. Instead, the rate is primarily governed by interaction of O_2_ with the thiophenolato-bound dicobalt complex.

### Reaction of Co_2_L^1,3^ with O_2_ and Characterization of Intermediates in the Presence of Different Axial Ligands

To elucidate the origin of the axial-ligand-dependent ORR selectivity, we characterized the oxygenated intermediates formed upon reaction of **Co**_**2**_ **L** ^**1,3**^ **-X** with O_2_. These studies were carried out in MeCN at −40 °C, allowing direct spectroscopic observation of intermediates relevant to the catalytic ORR pathways.

Bubbling of O_2_ into a MeCN solution of **Co**_**2**_ **L** ^**1,3**^ **-MeCN** at −40 °C resulted in the formation of an orange species **I-MeCN**, with absorption maxima at 410 nm (*ε* = 12320 M^–1^ cm^–1^), 522 nm (*ε* = 1680 M^–1^ cm^–1^), and 778 nm (*ε* = 512 M^–1^ cm^–1^) (Figure 3a, red). Formation of **I-MeCN** was reversible upon Ar purging and warming/cooling cycles, suggesting reversible O_2_ binding (Figures S25–S26). The intermediate **I-MeCN** was further characterized by rRaman (rR), ^1^H NMR, XAS, and EPR studies (Figures 3, 4 and Figures S25–S29). The rR spectrum of **I-MeCN** in MeCN prepared with ^16^O_2_ revealed three isotopically sensitive vibrational modes at 829, 664, and 642 cm^–1^, which shifted to 786, 642, and 614 cm^–1^, respectively upon labeling with ^18^O_2_. The 829 cm^–1^ band with an isotope shift of 43 cm^–1^ is assigned to the O–O stretch of a peroxido ligand in **I-MeCN** (calculated shift based on Hooke’s law,^23^ (^16/18^Δ_calc._ = 47 cm^–1^)). The bands at 664 (^16/18^Δ_Exp._ = 23 cm^–1^, ^16/18^Δ_calc._ = 30 cm^–1^) and 642 cm^–1^ (^16/18^Δ_Exp._ = 28 cm^–1^, ^16/18^Δ_calc._ = 29 cm^–1^) are assigned to Co–O vibration modes of an asymmetric *μ*-(*η* ^2^ *:η* ^1^)-type of peroxido moiety in **I-MeCN** (Figure 3b, 5a). A rR investigation was also done in the presence of mixed isotope O_2_ (^16/18^O_2_) containing a mixture of ^16^O_2_, ^16/18^O_2_, ^18^O_2_, which further corroborates the asymmetric peroxido binding motif in **I-MeCN** (Figure 3b, inset). In the mixed-isotope spectrum, there is a mixture of several asymmetric substrates (the symmetric one may be involved as well) which leads to broad and asymmetric line shapes in the ^16^O and in the mixed isotope species (Figure 3b, inset). As a result, in the mixed isotope species, there is a distribution of several strongly overlapping bands, reflecting the distribution of asymmetric structures (Figure 3b, 5a).

**Figure 3:**
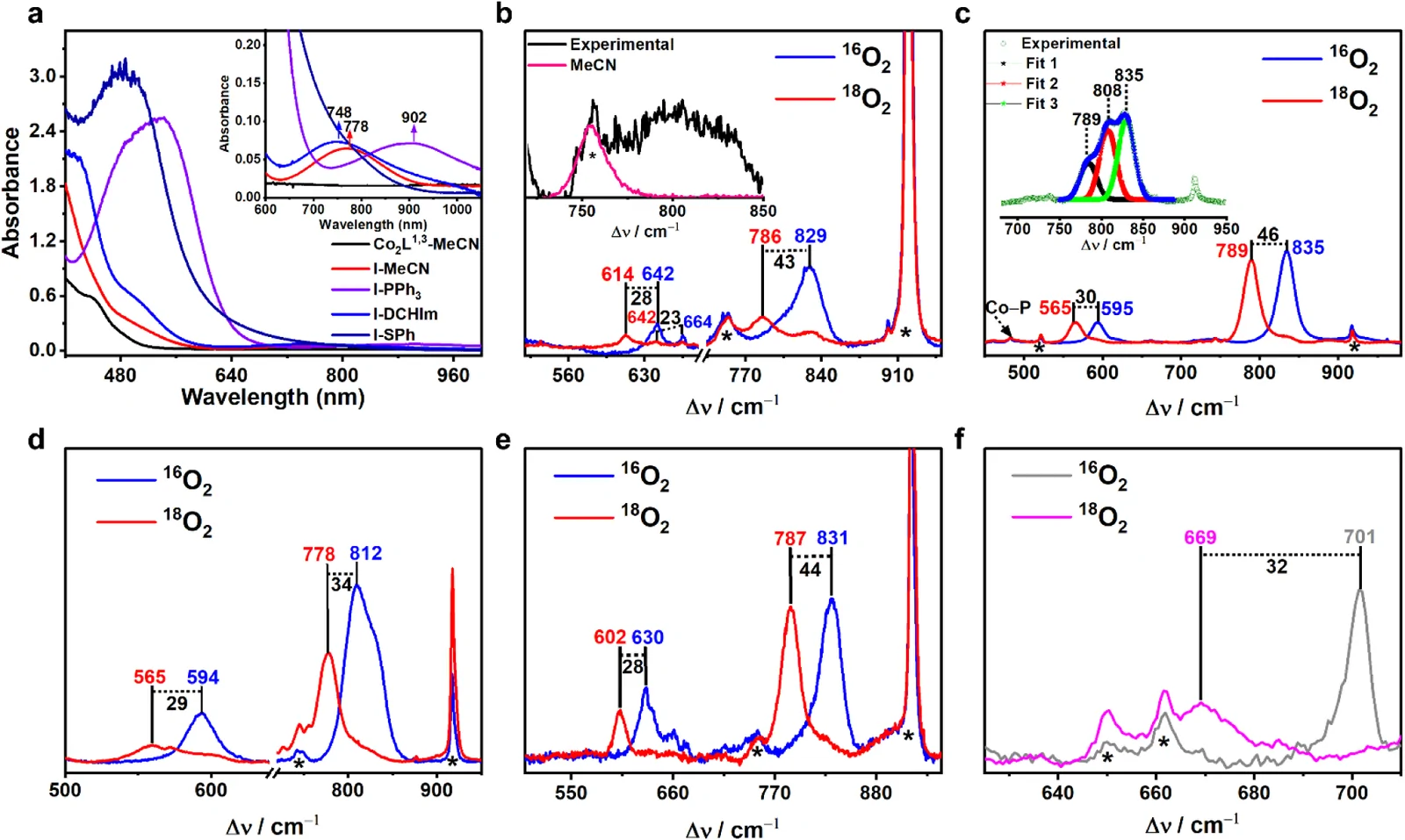
(a) UV–vis spectra of **Co_2_L^1,3^-MeCN** (black) and the oxygenated intermediates (**I-X**) in MeCN at −40 °C. Inset shows the low-energy absorption region highlighting diagnostic bands of the axial-ligand-bound intermediates. rR spectra of (b) **I-MeCN** (blue) and ^18^O_2_-enriched **I-MeCN** (red), and (c) **I-PPh_3_** (blue) and ^18^O_2_-enriched **I-PPh_3_** (red). Inset: rR spectra of **I-MeCN** (black) and **I-PPh_3_** (green) enriched with mixed isotope O_2_ (^16/18^O_2_) containing a mixture of ^16^O_2_, ^16/18^O_2_, ^18^O_2_ and its spectral deconvolution. (d) rR spectra of **I-SPh** (blue) and ^18^O_2_-enriched **I-SPh** (red). (e) rR spectra of **I-DCHIm** (blue) and ^18^O_2_-enriched **I-DCHIm** (red). (f) rR spectra of **D́** (grey) and ^18^O_2_-enriched **D́** (magenta) (λ_ex_ = 568 nm, T = −40 °C in MeCN). The peaks marked by’*’ originates from the solvent.

**Figure 4:**
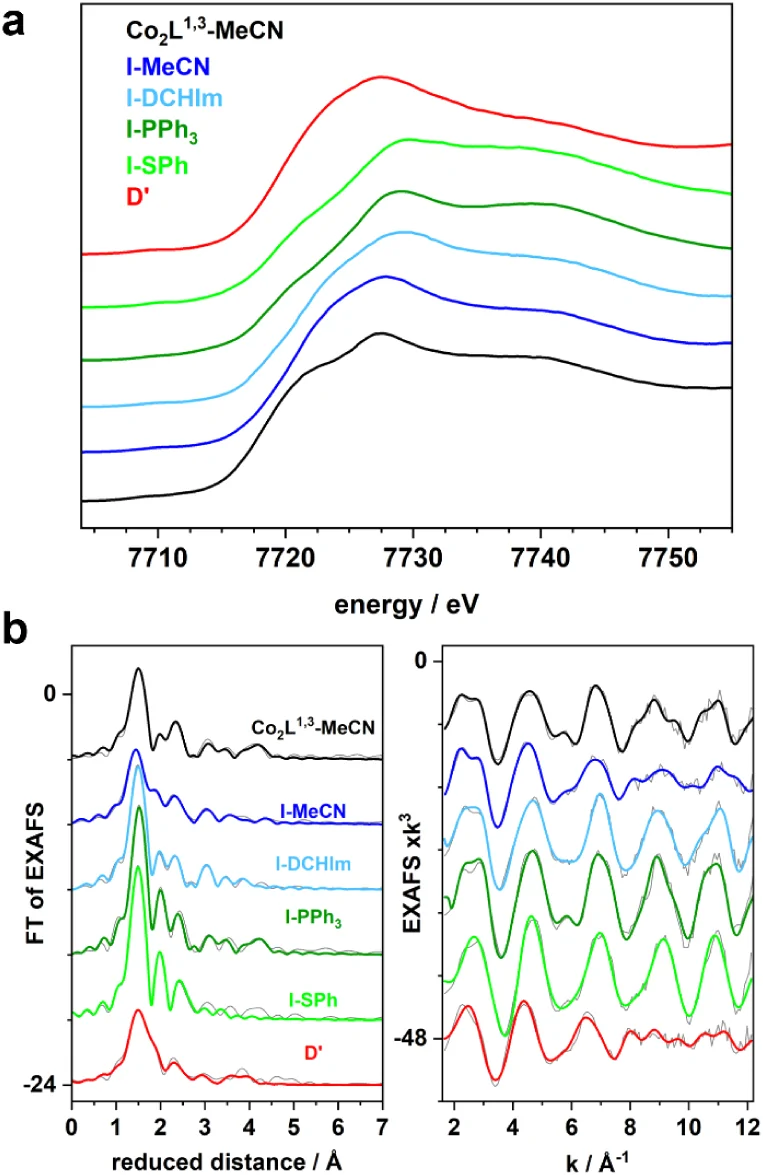
Co XAS spectra. (a) XANES spectra of indicated samples. Co K-edge energies (determined with the integral method in a 0.15–0.9 amplitude range) are shown in Figure S29. (b) EXAFS spectra. The left panels show FTs (experimental data) of EXAFS spectra in the right panel. Thin gray lines, experimental data; thick (colored) lines, simulations with parameters in Table S6. Spectra are vertically stacked for clarity.

**Figure 5:**
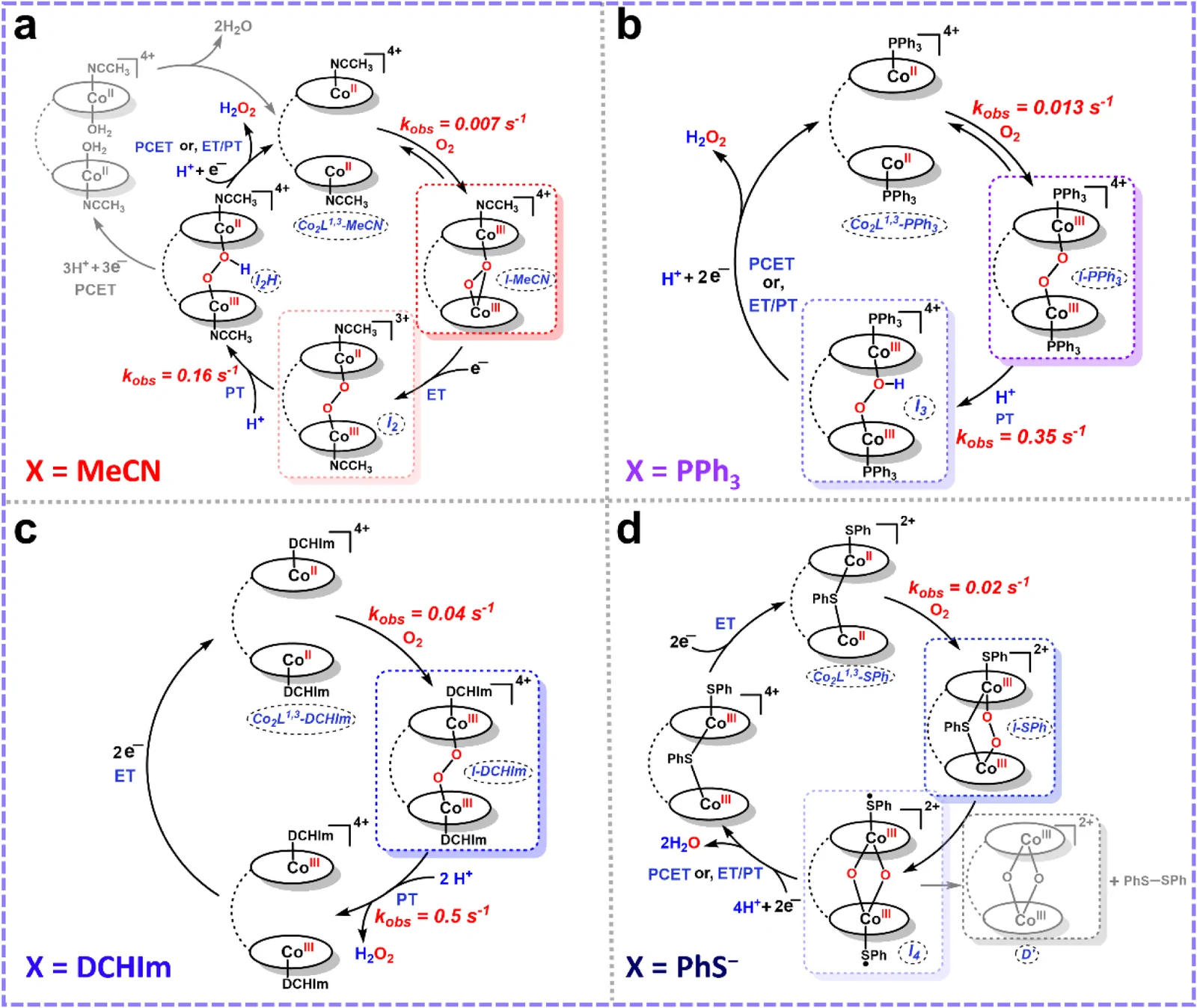
Proposed mechanisms of ORR catalyzed by (a) **Co_2_L^1,3^-MeCN**, (b) **Co_2_L^1,3^-PPh_3_**, (c) **Co_2_L^1,3^-DCHIm**, (d) **Co_2_L^1,3^-SPh.** The measured pseudo-first-order rate constants for the rate influencing O_2_-binding and proton-transfer steps are shown in red; experimental conditions are provided in the text and Supporting Information.The mechanisms shown in gray represent the minor four-electron pathway in (a) and an off-cycle formation of the bis-μ-oxido species and PhSSPh in (d).

Furthermore, **I-MeCN** was X-band EPR silent (Figure S27a) and gave diamagnetic ^1^H NMR when measured at −40 °C (Figure S28), consistent with a diamagnetic dicobalt­(III)–peroxido formulation. In agreement with this assignment, the Co K-edge spectrum of **I-MeCN** (7719.3 eV) showed an energy upshift of ca. 1.3 eV from **Co**_**2**_ **L** ^**1,3**^ **-MeCN** (7718.0 eV), which is in agreement with Co­(III) formation in **I-MeCN** (Figure 4a, Figure S29). The presence of a Co–O_2_ unit in **I-MeCN** was confirmed by EXAFS analysis, which yielded a shorter and a longer Co–O distance (1.85 Å, ∼2.36 Å, Table S6), besides of the four Co–N bonds of the bbpya unit and a Co–MeCN bond (Figure 4b). A Co–Co distance close to 3.3 Å may be tentatively assigned.

Addition of excess PPh_3_ (5 equiv) to **I-MeCN** at −40 °C led to significant changes in the UV-vis spectrum (Figure 3a, violet). The resulting deep purple solution (**I-PPh**_**3**_) featured absorption maxima at 490 nm (*ε* = 12160 M^–1^ cm^–1^), 535 nm (*ε* = 13360 M^–1^ cm^–1^), and 902 nm (*ε* = 650 M^–1^ cm^–1^) (Figure 3a, violet). The formation of **I-PPh**_**3**_ is also reversible in nature (Figure S30); the starting **Co**_**2**_ **L** ^**1,3**^ **-PPh**_**3**_ complex can be regenerated at higher temperatures and in Ar atmosphere. The EPR silence (Figure S27b), diamagnetic ^1^H-NMR (Figure S31a), and a Co K-edge energy of 7720.2 eV are all consistent with a dinuclear Co­(III) assignment of **I-PPh**_**3**_ (Figure 4a, Figure S29). Further support for phosphine coordination was obtained from ^31^P NMR spectroscopy, which shows a new resonance (*δ*_P_) at 16.04 ppm (no OPPh_3_ was detected from ^1^H NMR and GC-MS) in addition to free PPh_3_ (Figure S31b). The rR spectrum revealed the presence of a peroxido moiety in **I-PPh**_**3**_ (Figure 3c), albeit with a different binding motif relative to **IMeCN**. **I-PPh**_**3**_ exhibits a slightly higher *ν*(O–O) vibrational mode at 835 cm^–1^ (^16/18^Δ_Exp._ = 46 cm^–1^, ^16/18^Δ_calc._ = 48 cm^–1^) and significantly lower *ν*(Co–O) mode at 595 cm^–1^ (^16/18^Δ_Exp._ = 30 cm^–1^, ^16/18^Δ_calc._ = 27 cm^–1^) compared to **I-MeCN** (*ν*(O–O) = 829 cm^–1^ and *ν*(Co–O) = 664, 642 cm^–1^) (Table 1). The peroxido binding mode in **I-PPh**_**3**_ was further evaluated by a mixed-isotope rR experiment using an ^16/18^O_2_ mixture containing ^16^O_2_, ^16/18^O_2_, ^18^O_2_ isotopomers. In the *ν*(O–O) region, three components were observed between the *ν* ^16^O_2_- and *ν* ^18^O_2_-derived vibrations (Figure 3c, inset). This three-band pattern is expected for a peroxido core in which the two oxygen atoms are equivalent, because the two possible mixed isotopomers, ν^16^O^18^O and ν^18^O^16^O, are energetically indistinguishable and therefore collapse into a single intermediate *ν*(O–O) band. This behavior contrasts with **I-MeCN,** for which a mixture of several asymmetric lines are observed, consistent with inequivalent oxygen atoms in an asymmetric *μ*-(*η* ^2^:*η* ^1^)-peroxido unit. Thus, the mixed-isotope rR pattern of **I-PPh**_**3**_, together with the single isotope-sensitive Co–O vibration at 595 cm^–1^ supports assignment of **I-PPh**_**3**_ as *μ*-(*η* ^1^:*η* ^1^)-peroxido dicobalt­(III) species (Figure 5b).

**Table 1:** Comparison of the Frequencies and O^16^/O^18^ Shifts (in Brackets), Given in cm^–1^, of the O–O and Co–O Vibrational Modes

	**I-MeCN**	**I-DCHIm**	**I-PPh_3_**	**I-SPh**
ν(Co–O)[^16/18^Δ_Exp._]	664[−23],	630[−28]	595[−30]	594[−29]
	642[−28]			
ν(O–O)[^16/18^Δ_Exp._]	829[−43]	831[−44]	835[−46]	812[−47]

The axial-ligand-induced reorganization of the peroxido binding motif in **I-MeCN** and **I-PPh**_**3**_ is mechanistically significant. Notably, introduction of a stronger σ donating PPh_3_ ligand at the position *trans* to the peroxido ligand may cause such structural reorganization. Consistent with this interpretation, **I-PPh**_**3**_ exhibits a lower *ν*(Co–O) frequency corresponding to a longer Co–O distance than in **I-MeCN**, indicating weakening of the Co–O interaction (Table 1). Together with the greater steric demand of PPh_3_, these electronic effects likely contribute to the change from an asymmetric μ-(*η* ^2^:*η* ^1^)-peroxido core in **I-MeCN** to a μ-(*η* ^1^:*η* ^1^)-peroxido core in **I-PPh**_**3.**_

Addition of DCHIm to **I-MeCN** at −40 °C generated a deep orange intermediate, **I-DCHIm,** with absorption bands at 421 nm (*ε* = 16880 M^–1^ cm^–1^), 515 nm (*ε* = 4160 M^–1^ cm^–1^), and 748 nm (*ε* = 584 M^–1^ cm^–1^) (Figure 3a, blue). The ^1^H NMR spectrum and absence of an X-band EPR signal are consistent with a diamagnetic ground state of **I-DCHIm** (Figures 27c and S32). The Co oxidation state is closer to the trivalent level, as revealed by XANES (Figure 4a, Figure S29). The rR spectra of **I-DCHIm** show isotope-sensitive vibrational modes for *ν*(Co–O) and *ν*(O–O) at 630 cm^–1^ (^16/18^Δ_Exp._ = 28 cm^–1^, ^16/18^Δ_calc._ = 27 cm^–1^) and 831 cm^–1^ (^16/18^Δ_Exp._ = 44 cm^–1^, ^16/18^Δ_calc._ = 47 cm^–1^) (Figure 3e), respectively, supporting a dicobalt­(III)–peroxido formulation. Notably, unlike **I-MeCN** and **I-PPh**_**3**_, the formation of **I-DCHIm** is not thermally reversible and could be generated at room temperature. At this temperature it was sufficiently stable, which allowed its characterization by scXRD. The crystal structure of **I-DCHIm** revealed the end-on peroxide (*μ*-*η* ^1^:*η* ^1^-O_2_) motif, where DCHIm is bound trans to the Co–O bond (Scheme 2a). Although the connectivity and overall coordination geometry of **I-DCHIm** are unambiguous, the crystallographic data are not of sufficient quality to support a detailed quantitative analysis of subtle metrical parameters.

**Scheme 2:**
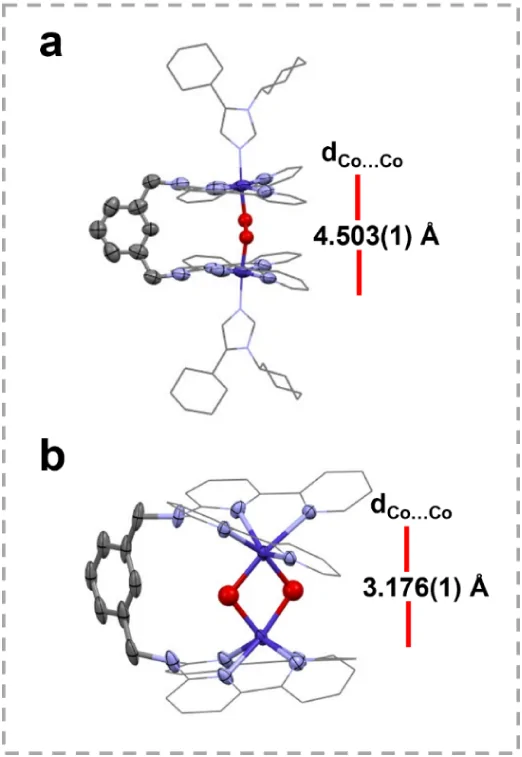
Molecular Structures of (a) I-DCHIm and (b) D́ with Ellipsoids Shown at 50% Probability[Fn sch2-fn2]

i Counter anions and hydrogen atoms are omitted for clarity

The behavior of the oxygenated intermediate changes dramatically in the presence of an anionic axial ligand. Addition of NaSPh to **I-MeCN** at −40 °C immediately generated a new intermediate, **I-SPh**, characterized by an intense absorption band at 486 nm (*ε* = 24100 M^–1^ cm^–1^) (Figure 3a, dark blue). rR measurements confirmed that the peroxido unit remains intact at low temperature, but with significantly weakened O–O and Co–O vibrational bands at 812 and 594 cm^–1^, respectively. Upon ^18^O_2_ substitution, these two bands shift down by 47 cm^–1^ (^16/18^Δ_calc._ = 46 cm^–1^) and 29 cm^–1^ (^16/18^Δ_calc._ = 30 cm^–1^), respectively (Figure 3d). This lower O–O stretching frequency in **I-SPh** relative to **I-X** (X = MeCN, PPh_3_ or DCHIm) (Table 1) species is consistent with stronger activation of the peroxido unit, presumably because of the strongly σ- and π-donating nature of the anionic thiophenolato ligand. The lower *ν*(Co–O) frequency of **I-SPh** (594 cm^–1^) is tentatively attributed to the reorganization of the dicobalt core associated with the bridging thiophenolato arrangement. The Co–O interaction is presumably weakened by increasing charge donation from the bridging thiophenolato group to the peroxido moiety. Nevertheless, Co K-edge XAS supports a dicobalt­(III) formulation of **I-SPh**, while EXAFS analysis is consistent with the presence of a Co–S bond (2.31 Å) in addition to Co–N/O distances from the ligand framework and the peroxido unit (Figure 4b).

Notably, similar to **I-DCHIm**, the formation of **I-SPh** is not thermally reversible. However, **I-SPh** is very unstable at room temparature. Upon warming, **I-SPh** undergoes an irreversible O–O bond cleavage to generate a thermally stable dicobalt­(III) bis-*μ*-oxido complex, **D́**, following first-order kinetics with rate constant (*k*_obs_) of 4.76 × 10^–2^ s^–1^ at room temperature (Figure S33). In this process, thiophenolato acts as an electron donor and is converted to diphenyl disulfide (PhS–SPh), as detected by GC–MS (Figure S34). The dicobalt­(III)-bis-μ-oxido complex was characterized by scXRD, which revealed a distorted octahedral geometry around the Co center and features Co–Co and Co–O distances of 3.1760(9) and 2.017(3) Å (Scheme 2b), respectively. The observed Co–Co and Co–O bonds are slightly longer as compared to previously reported “diamond core” dicobalt­(III)-bis-μ-oxido complexes.^24^ For **D́** in solution, EXAFS yielded Co–O distances of ca. 1.86 Å and 2.15 Å, which is more symmetric than in the **I-X** complexes, and a Co–Co distance (∼3.3 Å) slightly longer than in the crystal. The rR spectrum of **D́** shows an isotope-sensitive Co–O vibration at 701 cm^–1^, which shifts to 669 cm^–1^ upon ^18^O_2_ labeling (^16/18^Δ_Exp._ = 32 cm^–1^, ^16/18^Δ_calc._ = 32 cm^–1^), confirming that the oxido ligands originate from O_2_ (Figure 3f). This peroxido-to-bis-*μ*-oxido conversion and the identification of PhS–SPh in the reaction mixture provide direct evidence of the thiophenolato induced O–O bond cleavage within the dicobalt core.

### Reactivity of Peroxido Intermediates with Protons and Electrons and Catalytic ORR Mechanisms

The catalytic and spectroscopic data together indicate that O_2_ reduction by **Co**_**2**_ **L** ^**1,3**^ **-X** proceeds through dicobalt–peroxido intermediates whose structure and subsequent reactivity are controlled by the nature of the axial ligands. Because conversion of O_2_ to either H_2_O_2_ or H_2_O requires delivery of both protons and electrons we examined the reduction of the trapped peroxido intermediates (**I-X**, X = MeCN, PPh_3_, DCHIm, PhS^–^) by Fc* in the absence and presence of TFA, as well as the protonations of **I-X** in the absence and presence of Fc*.

In **Co**_**2**_ **L** ^**1,3**^ **-MeCN**, the ORR rate depends on the concentrations of the catalyst, O_2_, and TFA but shows no dependence on Fc* (Figure 2a–2d). This rate law indicates that O_2_ binding and proton delivery are involved in the kinetically relevant sequence, whereas electron transfer from Fc* is not rate-limiting.

Consistent with this behavior, the reaction of **Co**_**2**_ **L** ^**1,3**^ **-MeCN** (0.25 mM) with O_2_ (1.7 mM) at −40 °C generated the asymmetric dicobalt­(III)–peroxido intermediate **I-MeCN** within 300 s, following first-order kinetics with a rate constant (*k*_obs_) of 7.3 × 10^–3^ s^–1^ (Figure S35). **I-MeCN** did not react with TFA (Figure S36). However, in the presence of Fc* a fast decay of **I-MeCN** was observed suggesting electron transfer to the dicobalt­(III)–peroxido unit. The resulting species features an intense band at 624 nm and a slightly broad EPR signal consistent with a *S* = 3/2 ground state (Figure S37A) suggesting formation of the mixed valent Co^III^–O_2_–Co^II^ species (**I**_**2**_). In presence of TFA (10 mM) at −40 °C **I**_**2**_ is converted to the hydroperoxido species **I**_**2**_ **H** with *k*_obs_ = 0.16 s^–1^. **I**_**2**_ **H** retained an *S* = 3/2 EPR signal (Figure S37B). The accelerated decay of **I-MeCN** in the simultaneous presence of Fc* (1.5 mM) and TFA (10 mM) further supports a sequence in which electron transfer precedes and enables protonation (Figure S38). Thus, **I-MeCN** is a competent redox-active intermediate, but the absence of Fc* dependence in the catalytic rate law indicates that electron transfer is fast and not rate-limiting under catalytic conditions, whereas the observed acid dependence is consistent with the rate influencing protonation of the reduced O_2_-derived intermediate.

On the basis of these observations, we propose that ORR by **Co**_**2**_ **L** ^**1,3**^ **-MeCN** proceeds through initial O_2_ binding to the reduced dicobalt­(II) site to form **I-MeCN** (Figure 5a), which is then reduced very quickly to the mixed valent species. Further proton/electron transfer steps lead predominantly to H_2_O_2_ release from this intermediate, accounting for the major 2e^–^ ORR pathway. However, the same species can also undergo competing reduction and O–O bond cleavage, giving the minor 4e^–^ pathway to water (Figure 5a, gray). Thus, in the presence of a weak σ-donor MeCN, the O_2^2^_^–^ moiety in intermediate **I-MeCN** is electron deficient (Figure 5a), which leads to fast electron transfer and proton transfer events to form predominantly H_2_O_2_ and minor H_2_O.

For **Co**_**2**_ **L** ^**1,3**^ **-PPh**_**3**_, O_2_ binding to form **I-PPh**_**3**_ is also reversible and occurs with *k*_obs_ = 1.3 × 10^–2^ s^–1^ at −40 °C when 1.7 mM O_2_ was used (Figure S39). However, in contrast to **I-MeCN**, the O_2_^2–^ moiety in **I-PPh**_**3**_ is more nucleophilic and can’t be reduced by Fc* in the absence of TFA. In presence of 10 mM TFA only, it forms a hydroperoxido (**I**_**3**_) intermediate with a rate constant (*k*_obs_) 0.35 s^–1^, as shown by UV–vis and rR studies (Figure S40). **I**_**3**_ exhibits slightly lower *ν*(O–O) vibrational mode at 821 cm^–1^ (^16/18^Δ_Exp._ = 44 cm^–1^, ^16/18^Δ_calc._ = 47 cm^–1^) and a *ν*(Co–O) mode at 554 cm^–1^ (^16/18^Δ_Exp._ = 27 cm^–1^, ^16/18^Δ_calc._ = 27 cm^–1^) compared to **I-PPh**_**3**_ (*ν*(O–O) = 835 cm^–1^ & *ν*(Co–O) = 595 cm^–1^) (Figure S40c). Whereas **I**_**3**_ reacts very slowly with TFA alone at −40 °C, it decays with the liberation of H_2_O_2_ in the presence of TFA (10 mM) and Fc* (1.5 mM) (Figure S41). Thus, H_2_O_2_ release likely occurs through a proton coupled electron transfer (PCET) or uncoupled electron and proton transfer (ET/PT) processes from the hydroperoxido species rather than by simple protonation alone (Figure 5b).

A related but more direct peroxido-release pathway is observed for the DCHIm-bound intermediate. **I-DCHIm** forms with a rate constant (*k*_obs_) of 4.2 × 10^–2^ s^–1^ from the reaction of **Co**_**2**_ **L** ^**1,3**^ **-DCHIm** (0.25 mM) and O_2_ (0.2 mM) at −40 °C in MeCN (Figure S42). **I-DCHIm** also reacts with TFA (10 mM) in the absence of Fc* (Figure S43) with a rate constant of 0.53 s^–1^ at −40 °C. However, unlike **I-PPh_3_**, no isolable hydroperoxido intermediate is detected. Instead, protonation of **I-DCHIm** leads directly to H_2_O_2_ release and formation of a Co­(III) product (Figure 5c). This result suggests that the DCHIm-bound peroxido species is more nucleophilic (because of strong σ- and π-donations from DCHIm) and susceptible to protonolysis, such that any hydroperoxido intermediate formed by a single protonation event is too short-lived to accumulate. The enhanced nucleophilicity of **I-DCHIm** is supported by its reactivity with the electrophilic substrate 2-phenylpropionaldehyde (2-PPA) (Figure S44). Notably, **I-MeCN** and **I-PPh**_**3**_ showed no reaction with 2-PPA. The behavior of **I-DCHIm** is consistent with its catalytic selectivity, where H_2_O_2_ remains the major oxygen-reduction product.

**I-SPh**, which forms with a rate constant of (*k*_obs_) = 2.1 × 10^–2^ s^–1^ from the reaction of **Co**_**2**_ **L** ^**1,3**^ **-SPh** (0.25 mM) and O_2_ (0.2 mM) at −40 °C in MeCN (Figure S45) does not react with Fc* alone, suggesting that direct outer-sphere reduction of the thiophenolato-bound peroxido intermediate is not kinetically competitive. Instead, the peroxido-to-bis-μ-oxido conversion proceeds through intramolecular electron transfer from the bridging thiophenolato to the activated peroxido unit (Figure 5d). Notably, some heme and nonheme metal–thiolate systems have shown that anionic sulfur donors can promote O–O bond activation through a strong donor “push” effect and, in some cases, through direct redox participation of the sulfur ligand.^25^ Further support for the enhanced nucleophilic character of **I-SPh** comes from its reaction with 2-PPA, for which **I-SPh** displays the largest second-order rate constant among the peroxido intermediates examined (Figure S46) in this study. The mechanistic picture is consistent with the catalytic rate law for **Co**_**2**_ **L** ^**1,3**^ **-SPh**, where the rate depends on [**Co**_**2**_ **L** ^**1,3**^ **-SPh]** and [O_2_] but is independent of both TFA and Fc*. Thus, the rate-limiting step is most likely associated with O_2_ binding/peroxido activation and PhS^–^ mediated O–O bond cleavage via intramolecular electron-transfer, rather than external proton or electron deliveries. The identification of PhS–SPh during the decay of **I-SPh** provides evidence in support of PhS^–^ mediated O–O bond cleavage.

## Conclusions

In summary, we have demonstrated that the product selectivity of O_2_ reduction by a new flexible dicobalt platform can be switched by axial ligand coordination. The **Co**_**2**_ **L** ^**1,3**^ framework supports formation of O_2_-derived dicobalt­(III)–peroxido intermediates, which have been thoroughly characterized by UV-vis, rR, NMR, EPR, and XAS studies. The structure, reactivity, and subsequent fate of these intermediates are dictated by the nature of the axial ligands. Kinetic studies show that the slowest step in ORR mediated by **Co**_**2**_ **L** ^**1,3**^ **-X** (X = MeCN, PPh_3_, DCHIm or PhS¯) complexes involve O_2_ binding to the Co­(II) center, which is the rate-determining step (RDS; Figure 5). For **Co**_**2**_ **L** ^**1,3**^ **-X** (X = MeCN, PPh_3_, DCHIm), O_2_ binding is further coupled to proton transfer (PT) events, which also influence the rate of ORR, thereby, resulting in a third-order kinetic law (eq 1). For **Co**_**2**_ **L** ^**1,3**^ **-SPh**, in contrast, PT is not rate influencing and a second order kinetics prevail (eq 2). Notably, electron-transfer (ET) from Fc* is fast and does not influence the rate of ORR in all four cases; however, different moieties are involved in the reduction process. In the presence of a weak σ-donor (MeCN), ORR mediated by **Co**_**2**_ **L** ^**1,3**^ **-MeCN** involves reduction of an electrophilic peroxido **I-MeCN** (Figure 5a) unit. In contrast, **I-PPh**_**3**_, formed by O_2_ binding at **Co**_**2**_ **L** ^**1,3**^ **-PPh**_**3**_ and involving an axial ligand with a stronger σ-donor property than MeCN is more nucleophilic. Accordingly, it needs to be first protonated to the corresponding hydroperoxido intermediate **I**_**3**_ before any ET from Fc* can occur (Figure 5b). Thus, catalytic cycles for ORR mediated by **Co**_**2**_ **L** ^**1,3**^ **-MeCN** and **Co**_**2**_ **L** ^**1,3**^ **-PPh**_**3**_ involve reductions of **I-MeCN** and singly protonated **I-PPh**_**3**_ moieties, respectively, which presumably occur at potentials more negative than the Co^III/II^ potentials. This is also reflected in the onset potentials for catalytic currents at −0.16 V and −0.24 V for **Co**_**2**_ **L** ^**1,3**^ **-MeCN** and **Co**_**2**_ **L** ^**1,3**^ **-PPh**_**3**_, respectively, (Figure S14) in electrocatalytic ORR studies, which are significantly cathodically shifted relative to the measured Co^III/II^ potentials (*E*_*1/2*_ = 0.58 V for **Co**_**2**_ **L** ^**1,3**^ **-MeCN** and 0.30 V for **Co**_**2**_ **L** ^**1,3**^ **-PPh**_**3**_). Notably, for **Co**_**2**_ **L** ^**1,3**^ **-MeCN** and **Co**_**2**_ **L** ^**1,3**^ **-PPh_3_**, O_2_ binding is reversible and not favored at room temperature, which may also contribute to the observed overpotential necessary for electrocatalytic ORR. In contrast, **Co**_**2**_ **L** ^**1,3**^ **-DCHIm** binds O_2_ irreversibly at room-temperature to form **I-DCHIm**, and is even more nucleophilic than **I-PPh**_**3**_ owing to stronger σ-donor properties of DCHIm. Accordingly, **I-DCHIm** can be directly protonated to yield H_2_O_2_ (Figure 5c). Fc* is now utilized to regenerate the Co­(II) precursor by reduction of Co­(III); correspondingly, the onset potential (−0.10 V) is close to the Co^III/II^ potential (−0.16 V). Coordination of an anionic thiophenolato ligand, which is strongly σ- and π-donating, makes the O_2_^2–^ moiety in **I-SPh** unstable against irreversible O–O bond cleavage by an intramolecular ET step from PhS¯ to O_2^2^_^–^, leading to near-quantitative 4e^–^/4H^+^ reduction to water. The intermediate (**I**_**4**_) formed upon O–O bond cleavage is transient, and is instantly converted to H_2_O and **Co**_**2**_ **L** ^**1,3**^ **-SPh** in the presence of H^+^ and Fc* (Figure 5d). These make the proton and electron transfer steps not rate influencing. Also, the electrochemical ORR occurs at potential close to the Co^III/II^ potential, consistent with the mechanism shown in Figure 5d. Thus, the present study demonstrates that within the same Co–N_4_ manifold, the nature of the axial ligand controls the 2e^–^/2H^+^ vs 4e^–^/4H^+^ ORR selectivity. While a strong σ-donating and π-accepting axial ligand like PPh_3_ prefers 2e^–^/2H^+^ ORR to H_2_O_2_, strong σ- and π- axial donation is a prerequisite for the 4e^–^/4H^+^ selectivity. Notably, in biology, cysteine has been discussed as the premier amino acid for O–O bond cleavage.^26^ Through its highly reactive and versatile thiolate group, cysteine is proposed to facilitate heterolytic O–O bond cleavage by serving as a potent proximal ligand in metalloenzymes.^26b,27^ In the present study involving cobalt-dioxygen intermediates, we now show that the axially ligated thiolate ligand promotes a homolytic O–O bond cleavage leading to 4e^–^/4H^+^ ORR selectivity.

## Supplementary Material



## Notes

Experimental Section, synthesis of complexes and generation of intermediates and its reactivity, Figures S1–S47 (characterization of complexes, additional spectroscopic data, electrochemical data, and kinetics study), and Tables S1–S10 (for detailed crystallographic data, EPR, electrochemistry, and XAS data) (PDF)

## Abbreviations

ORR: Oxygen Reduction Reaction
PPh_3_: Triphenylphosphine
DCHIm: 2,6-dicyclohexylimidazole
TFA: Trifluoroacetic acid
Fc*: Decamethylferrocene
XAS: X-ray Absorption Spectroscopy
rR: Resonance Raman
