# Supplementary material for: Axial Ligand Tuning of the Catalytic Two- versus Four-Electron Reduction of Dioxygen by a Dinuclear Cobalt Complex
Source: J Am Chem Soc. 2026 Sep 30;148(40):43376–88. doi: 10.1021/jacs.6c12922 (PMC13674264; doi:10.1021/jacs.6c12922)
Supplement: Supplementary file 1 [file ja6c12922_si_001.pdf]

Supporting Information for:

Axial Ligand Tuning of the Catalytic Two- versus Four-Electron Reduction of Dioxygen by a Dinuclear Cobalt Complex

Ayan Bera[a], Xinyang Peng[a], Dibya Jyoti Barman[a], Michael Haumann[b], Sagie Katz[c], Dennis G.H. Hetterscheid[d], Peter Hildebrandt[c], and Kallol Ray*,[a]

[a]Department of Chemistry, Humboldt-Universität zu Berlin, Brook-Taylor-Straße 2, 12489 Berlin, Germany

[b]XAS@KMC-3, BESSY-II+, Helmholtz Center Berlin, Albert-Einstein-Straße 15, 12489 Berlin, Germany

[c]Department of Chemistry, Technische Universität Berlin, 10623 Berlin, Germany

[d]Leiden Institute of Chemistry, Leiden University, P.O. Box 9502, 2300 RA Leiden, The Netherlands

Table of Contents

1 Experimental section 3

1.1 Materials 3

1.2 Instrumentation and experimental methods 3

2 Syntheses of compounds and intermediates 5

2.1 Syntheses of ligands and complexes 5

2.2 Generation of Intermediates (I-X, X = MeCN, Ph_3P, DCHIm, and PhS^-) 6

2.3 Calculation of $2e^-/4e^-$ ORR selectivity from n. 6

2.4 Spectrophotometric iodometric quantification of H_2O_2 7

2.5 Resonance Raman sample preparation 7

3 Supplementary tables 8

4 Supplementary figures 17

5 References 50

1 Experimental section

1.1 Materials

The chemicals employed were purchased from the companies ABCR, ACROS, SIGMA-ALDRICH, TCI, Alfa Aesar, BLDpharm and used without further purification. Anhydrous solvents (acetonitrile, dimethyl formamide, diethyl ether, THF, butyronitrile) were purchased from CARL-ROTH GmbH under the tradename ROTIDRY (>99.5%, < 50 ppm H_2O) and degassed by freeze-pump-thaw methods and dried over activated molecular sieve (3 Å or 4 Å) prior to use. Dried and degassed acetone was purchased from THERMO SCIENTIFIC under the trade name ACRO SEAL (>99.8%, extra dry). Deuterated solvents and $^{18}O_2$ were purchased from EURISO-TOP. Mixed isotope O_2 ($^{16/18}O_2$) containing a mixture of $^{16}O_2$, $^{16/18}O_2$, $^{18}O_2$ was prepared following a same procedure as reported before.[1] Preparation and handling of air or water sensitive compounds were performed under an inert atmosphere using either Schlenk techniques or a glovebox GS111717 filled with nitrogen (N_2) from GS GLOVEBOX SYSTEMTECHNIK. Argon (Ar), N_2 of quality 5.0 were used for this purpose and were purchased from AIR LIQUIDE.

1.2 Instrumentation and experimental methods

UV-vis spectroscopy. The UV-Vis absorption spectra were recorded with an 8453 UV-Visible Spectroscopy system from Agilent. The measurements were carried out in 10 mm precision cuvettes made of SUPRASIL® quartz glass, the closures of which were equipped with a septum. The measurements at low temperatures were carried out by cooling the cuvette holder using a cooling thermostat USP-203-A from Unisoku Scientific Instruments. The analysis of the spectra was carried out with the software UV-Visible Chemstation from Agilent.

Elemental analysis. All elemental analyses were performed by the analytical service of the Institut für Chemie of the Humboldt-Universität zu Berlin. The percentages of Carbon, Hydrogen, Nitrogen and Sulphur were determined using an HEKAtech EURO EA 3000 analyzer. The reported values are the result of an average of two independent measurements.

EPR spectroscopy. CW EPR spectra were collected by using a Bruker EMXplus Instrument at a frequency of ca. 9.35 GHz (X-Band) in perpendicular mode. All samples were measured at an average temperature of 14 K as frozen solutions (powder spectra) by the use of a liq. helium recirculating cooling system provided by ColdEdge. Spin-Hamiltonian-based simulations of EPR spectra were performed using the EasySpin toolbox[2]. Quantification of the concentration of the paramagnetic species against a copper (II) standard of known concentration was carried out by calculation of the respective double integrals upon polynomial baseline correction.

Gas Chromatography (GC). GC analysis was carried out using an AGILENT 7890B gas chromatograph (HP5 column, 30 m) with a flame-ionization detector coupled to an EI-MS AGILENT 5977B spectrometer with a triple-axis detector. The instrument was equipped with an autoinjector AGILENT G4513A (injection of approx. 10 µL). The GC method starts with an oven temperature of 75 °C (hold time 0.5 min) and includes two ramps (ramp 1: 75 °C-190 °C, 10 °C/min; ramp 2: 190 °C-300 °C, 20 °C/min, hold time 10 min at 300 °C), a total run-time of 27.5 min and a solvent delay of 2.9 min. MS peaks were analyzed and compared with the library database of NIST MS Search 2.3. All EI-MS spectra of the detected peaks were in good agreement with the library database of the expected substances.

Nuclear magnetic resonance (NMR) spectroscopy. All NMR spectra were recorded at 20 °C using BRUKER NMR spectrometer (Avance DPX 300 MHz, Avance 400 MHz, Avance III 500 MHz) equipped with a cryostat. ^{1}H and ^{13}C NMR were recorded in deuterated solvents and chemical shifts (δ, ppm) referenced against residual protic solvent peaks. ^{13}C NMR which were recorded in D_2O is

referenced using DSS (4,4-dimethyl-4-silapentane-1-sulfonic acid, δ = 0 ppm). The ^{19}F NMR is referenced with respect to trichlorofluoromethane ($CFCl_3$).

Electrospray ionization mass spectrometry (ESI-MS). ESI-MS spectra in solution were recorded by using an ADVION EXPRESSION CMS spectrometer; acetonitrile was used as an eluent and the sample was directly injected into the instruments. The analysis of the data was carried out with the ADVION DATA EXPRESS Version 6.0.11.3.

Cyclic voltammetry (CV). Cyclic Voltammetry was carried out with a CHI 600E potentiostat using a 3 mm diameter glassy carbon disk electrode (ALS Co Ltd.) as working and a platinum wire (length 5 cm, diameter 0.5 mm; ALS Co Ltd.) as counter electrode. Prior to use, electrodes were polished with 0.05 µm alumina suspensions. $Ag/AgNO_3$ (100 mM $AgNO_3$ and 0.1 M $[N(n\text{-}Bu)_4]PF_6$ in MeCN) was used as the reference electrode. CV measurements were performed in a three-necked glass cell under an Ar atmosphere. 2 mL of 0.5 mM compound solution in Acetonitrile was taken in the presence of 100 mM $[N(n\text{-}Bu)_4]PF_6$ as the supporting electrolyte. Unless noted otherwise, a scan rate of 100 mV/s was applied. Ferrocene (Fc) was used as an internal reference, and the potential scale is normalized with respect to the potential of the $Fc^{+/0}$ couple.

Single crystal X-ray structure determinations (SC-XRD). Data collections were performed at 100 K on a BRUKER D8 VENTURE diffractometer by using Mo Kα radiation (λ = 0.71073 Å). The structure was solved with the XT solution program[3] using Intrinsic Phasing methods and by using Olex2 1.5[4] as the graphical interface. The model was refined with XL using full matrix least squares minimisation on F^2.[5]

X-ray absorption spectroscopy (XAS). XAS at the Co K-edge was performed at beamline KMC-3 at the BESSY-II synchrotron (Helmholtz Center Berlin, Germany) as described earlier[6] using a set-up including a Si[111] double-crystal monochromator, a 13-element energy-resolving Si-drift detector (RaySpec) for X-ray fluorescence monitoring, and DXP-XMAP pulse processing electronics (XIA). Samples were held at 20 K in a closed-cycle liquid-helium cryostat (Oxford). The energy axis of the monochromator was calibrated (accuracy ± 0.1 eV) using the K-edge spectrum of a cobalt foil (fitted reference energy of 7709 eV in the first derivative spectrum). The spot size on the samples was ca. 2 x 4 mm (vertical x horizontal) as set by a focusing mirror and slits. X-ray fluorescence spectra were collected using a continuous scan mode of the monochromator (scan duration ~7 min). Up to 4 scans were averaged (2 scans per sample spot) for signal-to-noise ratio improvement. XAS data were processed (dead-time correction, background subtraction, normalization) to yield XANES and EXAFS spectra using our earlier described procedures and in-house software.[7] K-edge energies were determined at half-height (50 % level of normalized absorption). k^3-weighted EXAFS spectra were simulated with in-house software and phase functions from FEFF9 (S_0^2 was 0.85 for Co).[8]

Resonance Raman. Resonance Raman spectra were measured of the sample in solution at −40 °C (Bruker cryostat) using a Horiba Jobin-Yvon LabRAM HR800 confocal Raman spectrometer. Spectra were excited with 568 nm using a Kr^+ (Coherent) laser. The laser power at the sample was 5 mW. In general, the spectra were accumulated for 10 minutes. The sample concentration in MeCN/acetone-d^6 was between 5 – 10 mM.

2 Syntheses of compounds and intermediates

2.1 Syntheses of ligands and complexes

The ligands **N, N-bis(2,2′-bipyrid-6-yl) amine (Hbbpya)**, and was synthesized as described in the literature.[9]

Synthesis of L[1,3]

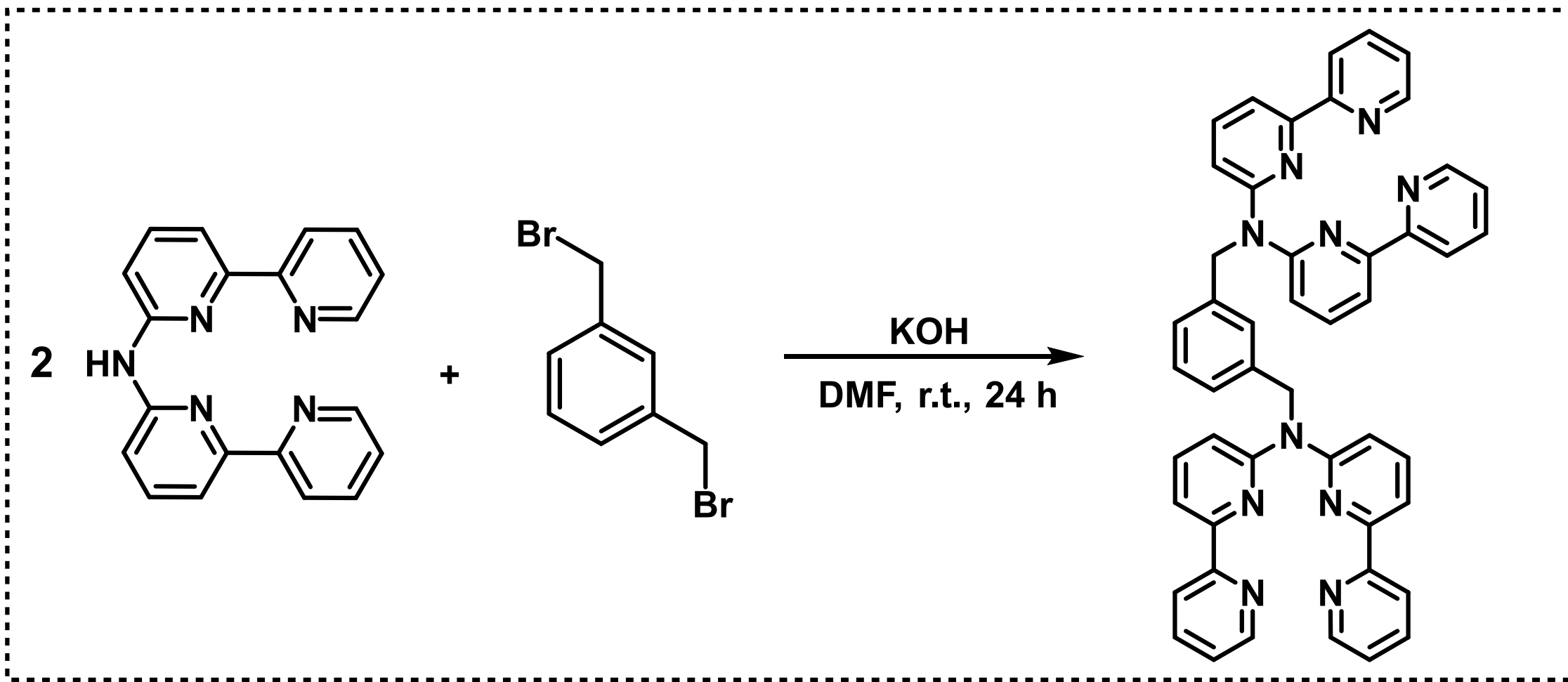

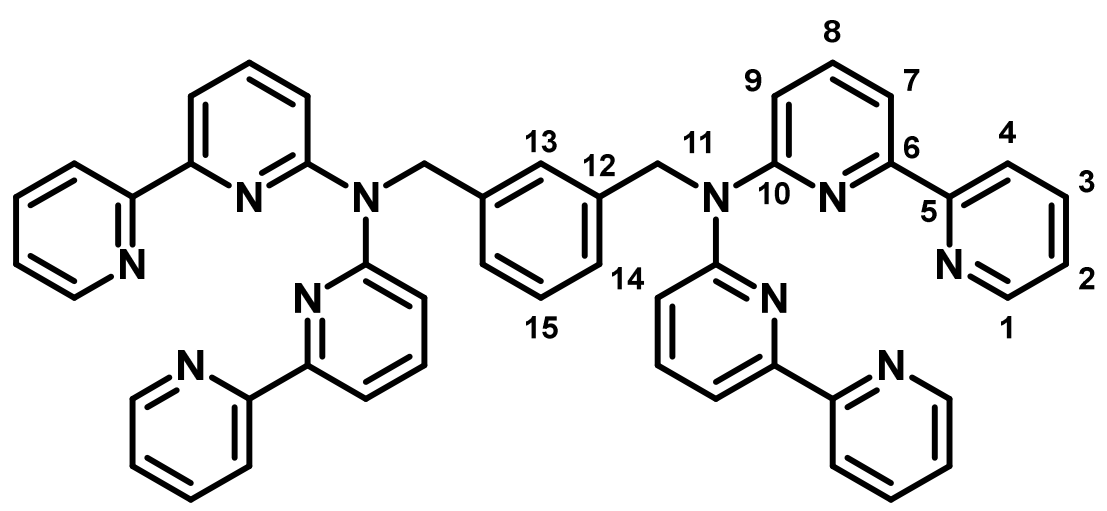

Hbbpya (834.1 mg, 2.563 mmol, 2.0 equiv.), potassium hydroxide (KOH) (358.8 mg, 6.408 mmol, 5.0 equiv.) and α,α′-dibromo-*m*-xylene (338.3 mg, 1.281 mmol, 1.0 eq.) were dissolved in 40 mL DMF. The reaction mixture was stirred for 24 h. With time, the color of the solution changed to brown. After stirring for 24 h, the DMF was removed under vacuo and the residue was redissolved in DCM, then washed twice with 50 mL of distilled water and additional brine. After that, the organic phases were collected, filtered through sodium sulphate, and then dried to a dark brown oil under vacuo. The brown oil was redissolved in a 4:1 *v/v* chloroform-acetone mixture, filtered through silica gel, and dried under vacuo. When the vacuum was applied, the product formed as a fine yellow powder. The reaction yielded (636 mg, 0.84 mmol, 66%) of $L^{1,3}$.

^{1}<u>H-NMR:</u> (300 MHz, $CDCl_3$) δ [ppm] = 8.59 (m, 4H, H_1), 8.07 (m, 4H, H_4), 7.86 (d, 4H, J = 7.9 Hz, H_7), 7.54 (td, J = 8.0, 1.1 Hz, 4H, H_8), 7.41 (t, 4H, H_3, H_{13}, H_{15}), 7.29 (m, 4H, H_9), 7.18 (m, 4H, H_2), 6.99 (d, J = 4.8 Hz, 4H, H_{14}), 5.61 Hz (s, 4H, H_{11}).

^{13}<u>C-NMR:</u> (75 MHz, $CDCl_3$) δ [ppm] = 156.29, 156.21, 154.28 (C_q), 148.97 (C_1), 138.01 (C_3), 136.82 (C_8), 128.28 (C_9), 125.62, (C_7), 125.51 (C_2), 123.52 (C_4), 121.07 (C_q), 114.47 (C_{14}), 114.10 (C_{15}), 51.45 (C_{11}).

Synthesis of $Co_2L^{1,3}$

For complexation, $L^{1,3}$ (70 mg, 0.09 mmol, 1.0 equiv.) was dissolved in 5 mL MeCN and stirred for ten minutes. Then $Co(OTf)_2$ (68 mg, 0.18 mmol, 2.1 equiv.) was dissolved in 5 mL MeCN and added to the solution. The color of the solution changed to yellow. After 24 hours of stirring at room temperature, the solution was filtered into an excess of Et_2O to precipitate out the complex. Then the yellow precipitate was dried under vacuo and subsequently washed several times with Et_2O to get $Co_2L^{1,3}$ (80

mg, 0.05 mmol, 58%). The complex was crystallized by vapor diffusion with DMF/Et_2O or, MeOH/Et_2O at room temperature.

ESI-MS: (positive) calculated m/z = 584.04 for $[Co_2L^{1,3}(OTf)_2]^{2+}$, found m/z = 584.4 (100%).

Synthesis of $Co_2L^{1,3}(BF_4)$

The complex $Co_2L^{1,3}(BF_4)$ was synthesised in similar procedure with $Co(BF_4)_2$ as metal precursor. $Co_2L^{1,3}(BF_4)$ was crystallized by vapor diffusion with MeCN/Et_2O at −20 ºC.

2.2 Generation of Intermediates (I-X, X = MeCN, Ph_3P, DCHIm, and PhS^-)

For generation of **I-MeCN**, a 0.5 mM solution of **$Co_2L^{1,3}$-MeCN** was prepared in dry, degassed MeCN and cooled to −40 °C. Dioxygen was then introduced by bubbling O_2 through the cold solution via a cannula for 30 s. Formation of **I-MeCN** was monitored by UV–vis spectroscopy by following the growth of the diagnostic absorption band at 778 nm.

For generation of the axial-ligand-bound intermediates **I-X** (X = Ph_3P, DCHIm, PhS^-), **I-MeCN** was first generated at −40 °C as described above. Subsequently, Ph_3P or DCHIm (5 equiv.), or NaSPh (2 equiv.), was added to the cold solution. Formation of the corresponding intermediates was monitored by UV–vis spectroscopy by following the characteristic absorption features described in the main text.

Alternatively, the same intermediates could be generated by first adding Ph_3P or DCHIm (5 equiv.), or NaSPh (2 equiv.), to a 0.5 mM solution of **$Co_2L^{1,3}$-MeCN** in dry, degassed MeCN at −40 °C, followed by introduction of O_2 by bubbling through the cold solution for 30 s. The resulting intermediates were identified by their characteristic UV–vis spectral features.

2.3 Calculation of $2e^-/4e^-$ ORR selectivity from n.

The experimentally determined electron-transfer number, n, represents the average number of electrons transferred per O_2 molecule consumed. Considering only reduction of O_2 to H_2O_2 through the $2e^-$ pathway and to H_2O through the $4e^-$ pathway, the product-pathway fractions can be obtained by electron balance as follows:

$$O_2 + 2H^+ + 2e^- \rightarrow H_2O_2 \qquad (1)$$

$$O_2 + 4H^+ + 4e^- \rightarrow 2H_2O \qquad (2)$$

If f_2e and f_4e are the fractions of O_2 undergoing the $2e^-$ and $4e^-$ pathways, respectively, then:

$$f_2e + f_4e = 1 \qquad (3)$$

$$n_{cat} = 2f_2e + 4f_4e \qquad (4)$$

Replacing $f_4e = (1 - f_2e)$ in equation 4, we get,

$$f_2e = (4 - n_{cat})/2 \quad \text{and} \quad f_4e = (n_{cat} - 2)/2 \qquad (5)$$

Accordingly, the pathway selectivities are calculated as:

$$\%H_2O_2 \text{ pathway} = 100(4 - n_{cat})/2 = 50(4 - n_{cat}) \qquad (6)$$

$$\%H_2O \text{ pathway} = 100(n_{cat} - 2)/2 = 50(n_{cat} - 2) \qquad (7)$$

For $Co_2L^{1,3}$-MeCN, n_{cat} = 2.55:

$\%H_2O_2$ pathway = 50(4 − 2.55) = 72.5%

$\%H_2O$ pathway = 50(2.55 − 2) = 27.5%

2.4 Spectrophotometric iodometric quantification of H_2O_2

For catalysis, **$Co_2L^{1,3}$-X** (X = Ph_3P, DCHIm, PhS^-) complexes were prepared by adding Ph_3P or DCHIm (5 equiv.), or NaSPh (2 equiv.), to a 10 µM solution of **$Co_2L^{1,3}$-MeCN** in dry, degassed MeCN. Chemical ORR was performed in air-saturated MeCN at 298 K using **$Co_2L^{1,3}$-X** (10 µM), O_2 (1.7 mM), Fc* (1 mM), and TFA (10 mM). Completion of the reaction was established from the plateau of the Fc^{*+} absorption at 778 nm. The reaction mixture was then passed through a short mini-column containing silica and $MgSO_4$ (3:1) to remove the cobalt-containing species. A 100 µL aliquot of the filtrate was diluted with MeCN to a final assay volume of 2 mL, corresponding to a dilution factor of 20 and subsequently it was purged with Ar to remove residual O_2. NaI was added to give a final iodide concentration of 0.10 M. UV–vis spectra were recorded at 25 °C in a 1 cm quartz cuvette until the absorbance at 361 nm reached a constant value. The spectrum of the identically treated sample before addition of NaI was used for background correction.

Under acidic conditions, H_2O_2 oxidizes iodide to iodine, which is trapped by excess iodide as I_3^-. The individual and net reactions are:

$H_2O_2 + 2\,I^- + 2\,H^+ \rightarrow I_2 + 2\,H_2O$ (8)

$I_2 + I^- \rightleftharpoons I_3^-$ (9)

$H_2O_2 + 3\,I^- + 2\,H^+ \rightarrow I_3^- + 2\,H_2O$ (10)

Equation 10. establishes a 1:1 molar relationship between H_2O_2 and I_3^-. The background-corrected absorbance was calculated as:

$\Delta A_{361} = A_{361}(\text{with NaI}) - A_{361}(\text{without NaI})$ (11)

The I_3^- concentration in the diluted assay solution was obtained from the Beer–Lambert law:

$\Delta A_{361} = \varepsilon_{361}.l.[I_3^-]\text{assay}$ (5)

$[I_3^-]\text{assay} = \Delta A_{361}/(\varepsilon_{361}.l) = [H_2O_2]\text{assay}$ (12)

where ε_{361} is the molar absorption coefficient of I_3^- at 361 nm and l is the optical path length. For direct calculation, $\varepsilon_{361} = 2.80 \times 10^4\ M^{-1}\ cm^{-1}$ and l = 1.00 cm were used.[10]

2.5 Resonance Raman sample preparation

The Co concentration used is 5-10 mM in MeCN. For the $^{16}O_2$-labeled intermediates, a solution of the corresponding starting complex **$Co_2L^{1,3}$-X** (X = MeCN, Ph_3P, DCHIm, PhS^-) in a NMR tube was cooled to the temperature required for intermediate formation. O_2 from a balloon (1 bar) was bubbled through the solution for 1 min, after which the sample was rapidly frozen in liquid N_2.

For the $^{18}O_2$-labeled samples, $^{18}O_2$ gas (0.3 bar) was condensed and subsequently allowed to warm and expand into a small balloon. Although, identical concentration of Co is used for the $^{16}O_2$ and $^{18}O_2$ samples, the signals corresponding to the O-O and Co-O vibrations are in general lower for 18O2, because of the lower pressure of O_2 used, which may lead to lower yields and stability of the Co-O_2-Co intermediates. The gas was then bubbled through a precooled solution of the corresponding complex at −40 °C. The sample was immediately defrosted and transferred to a Raman cell precooled to −40 °C, and the spectrum was recorded at −40 °C.

3 Supplementary tables

Table S1. Crystallographic data for **$Co_2L^{1,3}$(OTf)**.

CCDC No.	2561981
Chemical formula	$0.047(C_{51}H_{41.951}Co_2F_6N_{10}O_{7.951}S_2)\cdot 0.047(C_{52}H_{44}Co_2F_6N_{10}O_8S_2)\cdot 0.186(CF_3O_3S)\cdot 0.076$[C2H3N]·0.047[CH4O]
M_r	146.47
Crystal system, space group	Triclinic, *P-1*
Temperature (K)	100
a, *b*, *c* (Å)	15.176 (4), 16.788 (6), 24.646 (7)
α, β, γ (°)	81.922 (9), 81.430 (11), 87.380 (9)
V (Å^3)	6146 (3)
Z	43
Radiation type	Mo *K*α
μ (mm^{-1})	0.79
Crystal size (mm)	0.2 × 0.06 × 0.02
T_{min}, T_{max}	0.646, 0.745
No. of measured, independent and observed [$I > 2\sigma(I)$] reflections	305164, 25251, 18943
R_{int}	0.099
$(\sin \theta/\lambda)_{max}$ (Å^{-1})	0.627
$R[F^2 > 2\sigma(F^2)]$, $wR(F^2)$, S	0.103, 0.255, 1.06
No. of reflections	25251
No. of parameters	1734
No. of restraints	7
H-atom treatment	H atoms treated by a mixture of independent and constrained refinement
$\Delta\rho_{max}$, $\Delta\rho_{min}$ (e Å^{-3})	2.94, −1.46

Table S2. Crystallographic data for **$Co_2L^{1,3}(BF_4)$**.

CCDC No	2561977
Chemical formula	$0.053(C_{52}H_{42}Co_2N_{12})\cdot 0.211(BF_4)\cdot 0.105(C_2H_3N)\cdot 0.192[C_2H_3N]$
M_r	80.53
Crystal system, space group	Triclinic, *P-1*
Temperature (K)	100
a, b, c (Å)	12.0749 (18), 16.853 (2), 19.355 (3)
α, β, γ (°)	113.660 (4), 94.178 (4), 104.302 (4)
V (Å^3)	3429.6 (9)
Z	38
Radiation type	Mo Kα
μ (mm^{-1})	0.58
Crystal size (mm)	0.45 × 0.3 × 0.08
Diffractometer	Bruker APEX-II CCD
No. of measured, independent and observed [I > 2σ(I)] reflections	100556, 13367, 9068
R_{int}	0.146
$(\sin \theta/\lambda)_{max}$ (Å^{-1})	0.617
Refinement	
$R[F^2 > 2\sigma(F^2)]$, $wR(F^2)$, S	0.087, 0.241, 1.05
No. of reflections	13367
No. of parameters	833
H-atom treatment	H-atom parameters constrained
$\Delta\rho_{max}$, $\Delta\rho_{min}$ (e Å^{-3})	1.361, −1.544

Table S3. Crystallographic data for **$Co_2L^{1,3}$-DCHIm**.

CCDC No	2561979
Chemical formula	$C_{78}H_{82.187}Co_2N_{14}·4(BF_4)·2[C_2H_3N]$
M_r	1762.97
Crystal system, space group	Orthorhombic, *Pnma*
Temperature (K)	100
a, *b*, *c* (Å)	12.4626 (7), 34.4258 (19), 18.8864 (10)
V (Å^3)	8102.9 (8)
Z	4
Radiation type	Mo $K\alpha$
μ (mm^{-1})	0.50
Crystal size (mm)	0.12 × 0.07 × 0.04
T_{min}, T_{max}	0.651, 0.745
No. of measured, independent and observed [$I > 2\sigma(I)$] reflections	180244, 8469, 4422
R_{int}	0.184
$(\sin \theta/\lambda)_{max}$ (Å^{-1})	0.627
$R[F^2 > 2\sigma(F^2)]$, $wR(F^2)$, S	0.067, 0.203, 1.02
No. of reflections	8469
No. of parameters	572
No. of restraints	495
H-atom treatment	H-atom parameters constrained
$\Delta\rho_{max}$, $\Delta\rho_{min}$ (e Å^{-3})	0.57, −0.36

Table S4. Crystallographic data for **I-DCHIm**.

CCDC No	2561980
Chemical formula	$0.022(C_{76}H_{84}Co_2N_{14}O_2)\cdot 0.022(C_{78}H_{85}Co_2N_{14}O_2)\cdot 0.177(CF_3O_3S)$ $\cdot 0.045(C_4H_8O)$
M_r	90.63
Crystal system, space group	Monoclinic, *P*2/*c*
Temperature (K)	100
a, *b*, *c* (Å)	31.8390 (18), 12.8843 (7), 24.3292 (13)
β (°)	107.526 (2)
V (Å^3)	9517.1 (9)
Z	89
Radiation type	Mo *K*α
μ (mm^{-1})	0.53
Crystal size (mm)	0.10 × 0.06 × 0.04
Diffractometer	Bruker D8 Venture
T_{min}, T_{max}	0.643, 0.745
No. of measured, independent and observed [$I > 2\sigma(I)$] reflections	289840, 19612, 13671
R_{int}	0.095
$(\sin \theta/\lambda)_{max}$ (Å^{-1})	0.628
Refinement	
$R[F^2 > 2\sigma(F^2)]$, $wR(F^2)$, S	0.140, 0.440, 1.77
No. of reflections	19612
No. of parameters	1341
No. of restraints	501
H-atom treatment	H-atom parameters constrained
$(\Delta/\sigma)_{max}$	1.400
$\Delta\rho_{max}$, $\Delta\rho_{min}$ (e Å^{-3})	3.57, −1.81

Table S5. Crystallographic data for **$Co_2L^{1,3}$-SPh** and **D´**.

CCDC No	2563723	2561978
Chemical formula	$C_{114}H_{85}Co_4N_{20}S_3 \cdot 5(CF_3O_3S)$	$0.069(C_{48}H_{36}Co_2N_{10}O_2) \cdot 0.276(C_3H_7NO) \cdot 0.138(BF_4)$
M_r	916.33	94.40
Crystal system, space group	Triclinic, *P-1*	Triclinic, *P-1*
Temperature (K)	100	102
a, *b*, *c* (Å)	15.2480 (18), 15.2653 (17), 32.841 (3)	10.2711 (9), 15.8635 (12), 20.0880 (17)
α, β, γ (°)	86.543 (3), 80.667 (4), 64.187 (3)	112.397 (3), 90.311 (3), 92.267 (3)
V (Å^3)	6789.9 (12)	3023.0 (4)
Z	2	29
Radiation type	Mo $K\alpha$	Mo $K\alpha$
μ (mm^{-1})	0.27	0.64
Crystal size (mm)	0.4 × 0.08 × 0.03	0.12 × 0.02 × 0.01
Diffractometer	Bruker D8 Venture	Bruker *APEX*-II CCD
T_{min}, T_{max}	0.673, 0.745	0.673, 0.745
No. of measured, independent and observed [$I > 2\sigma(I)$] reflections	287224, 23512, 19083	177068, 12454, 9068
R_{int}	0.069	0.131
$(\sin \theta/\lambda)_{max}$ (Å^{-1})	0.595	0.628
$R[F^2 > 2\sigma(F^2)]$, $wR(F^2)$, S	0.089, 0.268, 1.06	0.083, 0.252, 1.03
No. of reflections	23512	12454
No. of parameters	1837	837
H-atom treatment	H-atom parameters constrained	H-atom parameters constrained
$\Delta\rho_{max}$, $\Delta\rho_{min}$ (e Å^{-3})	4.26, −1.21	2.54, −1.01

Table S6: Co-EXAFS simulation parameters.

(coordination number, N [per Co], interatomic distance, R [Å], Debye-Waller parameter, $2\sigma^2$ x10^3 [$Å^2$], fit error sum, R_F(1-4 Å) [%]); *, fixed parameter; #, coupled parameter)

shell	Co-N	Co-N/S/P	Co-O	Co-O	Co-C	Co-C	Co-Co	R_F
**$Co_2L^{1,3}$−MeCN**								
N	4*	1,0	0,3#	0.3#	3*	3*	0,8	
R	1.94	2.25	1.85	2.41	2.84	3.78	4.43	25.3
$2\sigma^2$	13	2*	2*	2*	17#	17#	10*	
**I-MeCN**								
N	4*	1.2	0.5#	0.5#	3*	3*	0.5	
R	1.97	2.17	1.84	2.36	2.78	3.92	3.27	16.7
$2\sigma^2$	16	2*	2*	2*	32#	32#	10*	
**I-DCHIm**								
N	4*	1.1	0.7#	0.7#	3*	3*	0.5	
R	1.92	2.19	1.84	2.37	2.76	3.75	3.61	16.4
$2\sigma^2$	8	2*	2*	2*	19#	19#	10*	
**I-PPh_3**								
N	4*	0.6	0.7#	0.7#	3*	3*	1.0	
R	1.93	2.32	1.85	2.63	2.85	3.71	3.86	15.2
$2\sigma^2$	6	2*	2*	2*	6#	6#	10*	
**I-SPh**								
N	4*	0.6	0.8#	0.8#	3*	3*	0.7	
R	1.92	2.31	1.85	2.56	2.80	3.84	3.63	14.7
$2\sigma^2$	7	2*	2*	2*	6#	6#	10*	
**D´**								
N	4*	0.3	0.8#	0.8#	3*	3*	0.8	
R	2.01	2.47	1.86	2.15	2.85	3.91	3.31	20.9
$2\sigma^2$	12	2*	2*	2*	38#	38#	10*	

Table S7. Comparison of the Selected Bond Lengths (in Å) (EXAFS determined) of **I-X** Intermediates.

	**I-MeCN**	**I-DCHIm**	**I-PPh$_3$**	**I-SPh**
Co–N_{ligand}	1.97	1.92	1.93	1.92
Co-N/P/S	2.17	2.19	2.32	2.31
Co–O_{peroxo}	1.84<br>2.36	1.84<br>2.37	1.85<br>2.63	1.85<br>2.56

Table S8. Summary of EPR parameters for **$Co_2L^{1,3}$-X** (where X = MeCN, Ph_3P, DCHIm, and PhS^-) complexes.

Complex	Spin state	*g* values	$A(^{59}Co)$ / MHz	Axial-ligand hyperfine / MHz	Linewidth / mT
$Co_2L^{1,3}$-MeCN	$S = 1/2$	2.280, 2.216, 2.023	33, 54, 264	$A(^{14}N)$ = 2, 27, 33	3.86
$Co_2L^{1,3}$-PPh_3	$S = 1/2$	2.291, 2.147, 1.975	46, 64, 213	$A(^{31}P)$ = 219, 211, 8	4.93
$Co_2L^{1,3}$-DCHIm	$S = 3/2$	intrinsic: 2.62, 2.18, 2.12 effective: 5.06, 4.29, 2.04	not resolved	not resolved	43.8
$Co_2L^{1,3}$-SPh	$S = 0$	no Co(II) signal detected	-	-	-

$Co_2L^{1,3}$-DCHIm zero-field-splitting parameters: $|D|$ = 53.3 GHz = 1.78 cm^{-1}; $|E|$ = 6.20 GHz = 0.207 cm^{-1}; $|E/D|$ = 0.116.

Table S9. Representative cofacial dicobalt ORR catalysts.

Catalyst	Mode and conditions	Activity metric	Selectivity	Ref.
**Co_2(DPX); Co_2(DPD);**	RRDE in aqueous acid; chemical ORR for Co_2(DPX) in PhCN with ferrocene derivatives/$HClO_4$	Co_2(DPX): k_{cat} = 3.6 × 10^5 M^{-1} s^{-1} (chemical)	RRDE H_2O selectivity: 72% (DPX), 80% (DPD); chemical Co_2(DPX) gives essentially 4e^- reduction without detectable H_2O_2	11
**Co_2(DPXM); Co_2(DPDM)**	Adsorbed-catalyst RRDE in aqueous acid	E_{cat} ≈ +0.24 and +0.25 V vs Ag/AgCl	H_2O selectivity: 52% (DPXM), 46% (DPDM)	11b
**(PCY)Co_2 series; especially (PCA)Co_2**	Adsorbed on edge-plane graphite; RRDE in 1.0 M $HClO_4$	$E_{1/2}$ = 0.41–0.47 V vs SCE; (PCA)Co_2 apparent k ≈ 2 × 10^4 M^{-1} s^{-1}	n = 3.5–3.9; (PCA)Co_2 n = 3.9 with only a small H_2O_2 fraction	12
**[Co_2(L)]**	Chemical: PhCN/Fc/TFA; electrochemical: CH_2Cl_2/TFA	$k$obs = 3.8 × 10^{-3} s^{-1}; 7–16 turnovers; Epc ≈ −0.27 V vs Fc^+/Fc (transient)	Little/no H_2O_2 above background; predominantly H_2O	13
**Co_2(LF)**	Chemical: PhCN, 0.4 M TFA, Me_2Fc or Fc, O_2 = 1.73 mM	$k$cat = 6.1 × 10^2 M^{-1} s^{-1}	n ≈ 3.58, favoring H_2O	14
**Co Prism**	Homogeneous chemical ORR and homogeneous RRDE; compared with monomeric CoTPyP	TOF = 66 h^{-1}; RRDE-derived kobs = 1.1 h^{-1} (CoTPyP: 6 and 0.05 h^{-1})	Faradaic selectivity evaluated; primary emphasis is the ≈10–20-fold cofacial rate enhancement	15
**Co-Tolyl/Phenyl/Chloro/CF_3 prisms**	Catalyst inks; RRDE in 0.5 M H_2SO_4	$E_{cat/2}$ = 0.35–0.38 V vs Ag/AgCl; ks = 0.9–3.4 × 10^5 M^{-1} s^{-1}	H_2O_2 = 1.6–10.7% (H_2O = 89.3–98.4%)	16
**Pacman dyads 1 and 2**	RRDE in 0.5 M H_2SO_4	$E_{1/2}$ = 0.51 V (1) and 0.70 V (2) vs RHE	n = 3.9 (1) and 3.8 (2)	17
**Py_3Ph and Py_2Ph_2 Co prisms**	Heterogeneous catalyst inks; aqueous RRDE	Standard ORR rate constants show ≈2 orders of magnitude cofacial enhancement over monomeric CoTPyP	H_2O_2 ≈ 5% (Py_3Ph) and ≈37% (Py_2Ph_2)	18
**Co-oNC**	Chemical homogeneous ORR and heterogeneous aqueous RRDE	Direct chemical/electrochemical comparison within one rigid cage	H_2O selectivity = 88% (chemical) and 83% (heterogeneous)	19
**$Co_2L^{1,3}$-MeCN**	Chemical: MeCN, Fc*, TFA; electrochemical: O_2-saturated MeCN/TFA RRDE	Chemical $k$obs = 5.1 × 10^{-3} s^{-1}	Chemical, n = 2.55; H_2O_2 = 68% by iodometry. RRDE n = 3.07	**this work**
**$Co_2L^{1,3}$-PPh_3**	Same chemical and RRDE protocols as above	Chemical $k$obs = 5.5 × 10^{-3} s^{-1}	Chemical n = 2.15; H_2O_2 = 94% by iodometry. RRDE n = 2.2	**this work**
**$Co_2L^{1,3}$-DCHIm**	Same chemical and RRDE protocols as above	Chemical k_{obs} = 4.5 × 1010^{-3} s^{-1}	Chemical n = 2.35; H_2O_2 = 88% by iodometry. RRDE n = 2.3	**this work**
**$Co_2L^{1,3}$-SPh**	Same chemical and RRDE protocols as above	Chemical k_{obs} = 1.15 × 10^{-2} s^{-1}	Chemical n = 3.95; no H_2O_2 detected. RRDE n = 3.63	**this work**

Table S10. Peak and midpoint potentials for the $\mathbf{Co_2L^{1,3}}$**-X** (where X = MeCN, Ph_3P, DCHIm, and PhS^-) series.

**Complex**	E_{pc} **(V)**	E_{pa} **(V)**	ΔE_p **(V)**	$E_{1/2}$ **(V)**
$Co_2L^{1,3}$-MeCN	+0.23	+0.92	0.69	+0.58
$Co_2L^{1,3}$-PPh_3	+0.17	+0.43	0.26	+0.30
$Co_2L^{1,3}$-DCHIm	−0.35	+0.01	0.36	−0.17
$Co_2L^{1,3}$-SPh	−0.29	−0.23	0.06	−0.26

All potentials are given in volts vs Fc^+/Fc. $E_{1/2} = (E_{pa} + E_{pc})/2$; $\Delta E_p = E_{pa} - E_{pc}$.

4 Supplementary figures

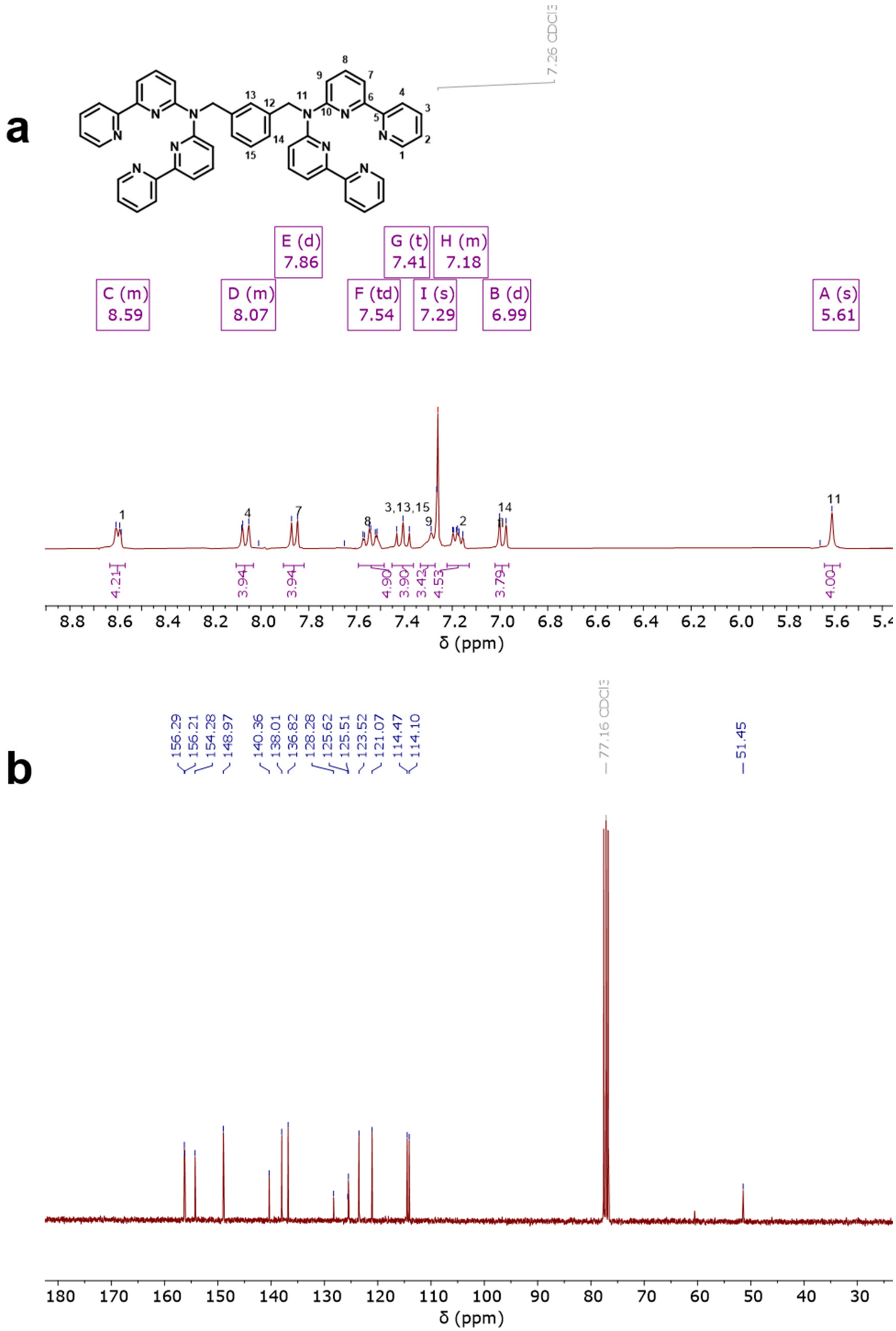

Figure S1. (a) ^{1}H-NMR and (b) ^{13}C-NMR spectrum of $\mathbf{L^{1,3}}$ in $CDCl_3$ at 298 K.

a

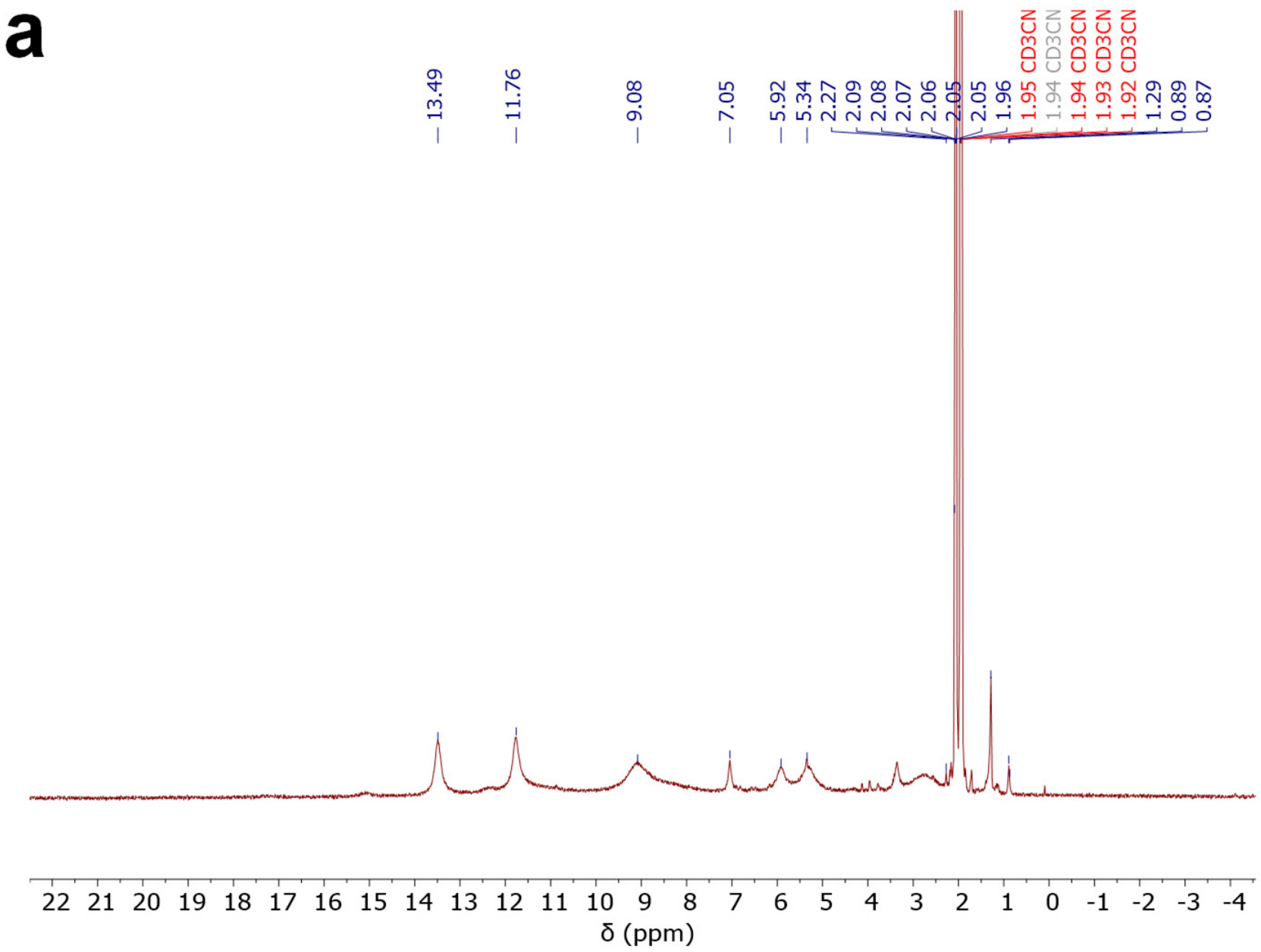

b

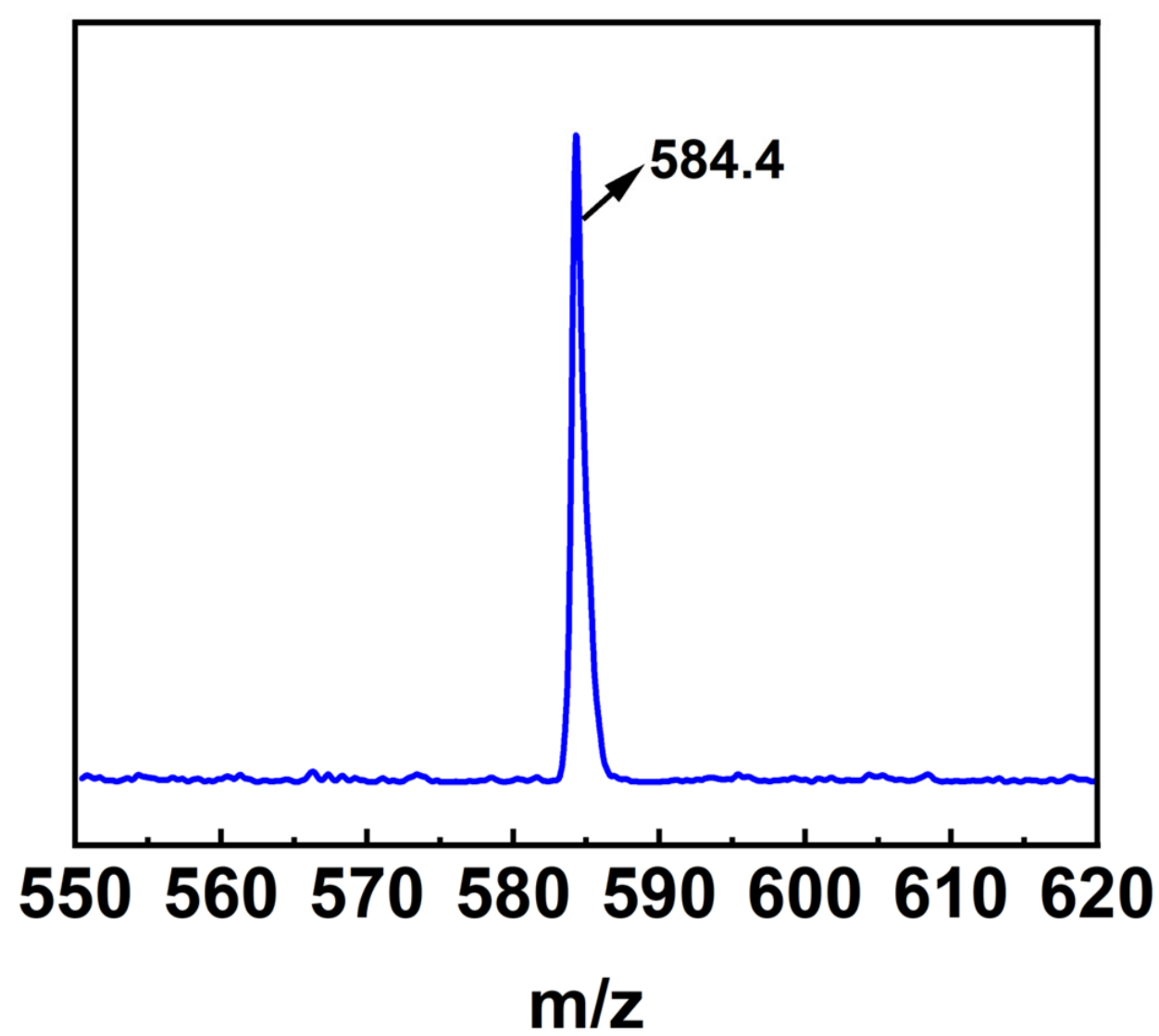

Figure S2. (a) ^{1}H-NMR spectrum of the **Co$_2$L^{1,3}(OTf)** in MeCN-d_3 at 298 K. (b) ESI-MS of **Co$_2$L^{1,3}(OTf)** in MeCN showing a dominant peak at m/z: 584.4 (calculated for $\{Co_2L^{1,3} + 2OTf\}^{2+}$.

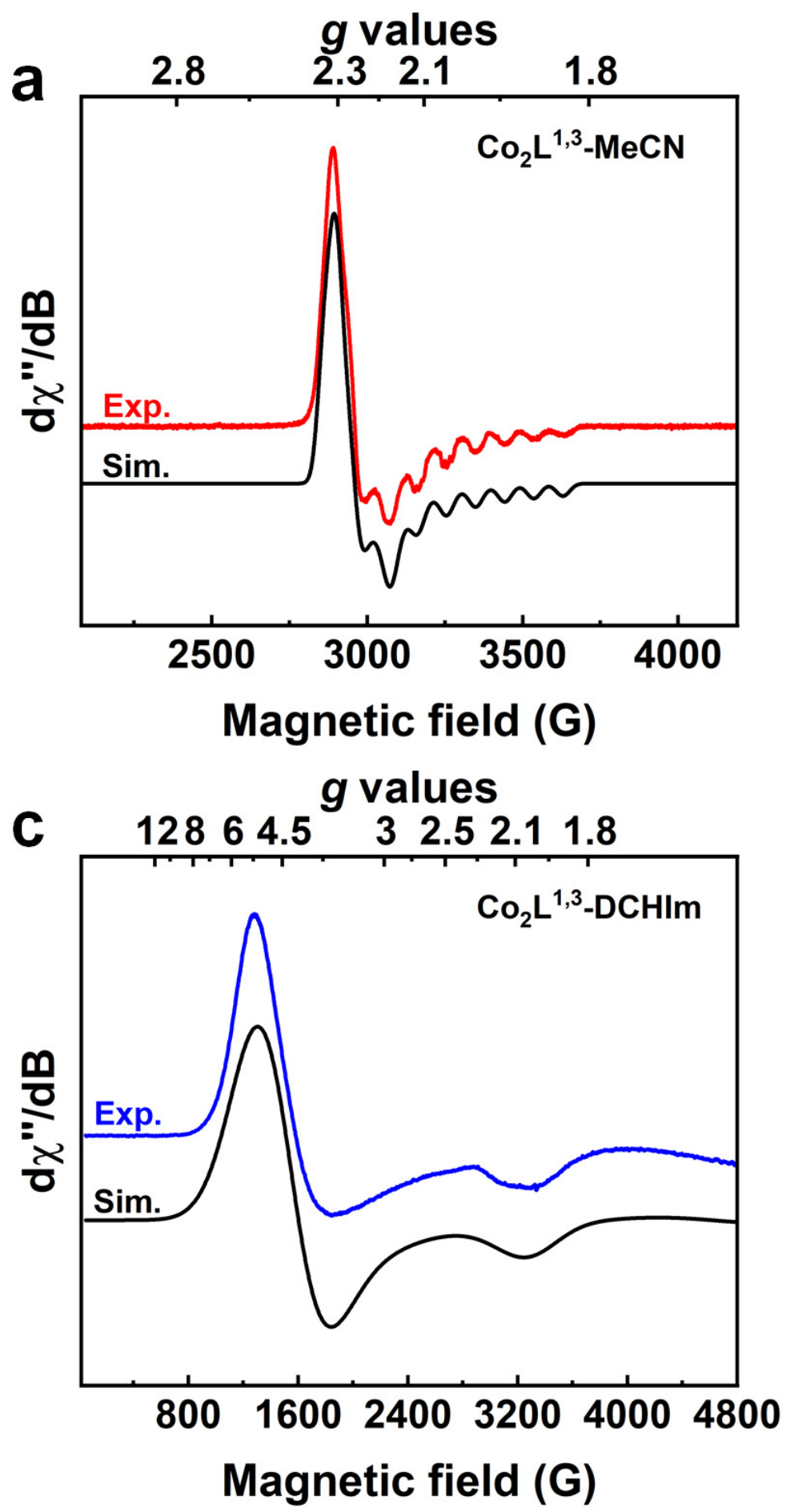

Figure S3. Experimental (colored traces) and simulated (black traces) frozen solution X-band EPR spectra of (a) **$Co_2L^{1,3}$-MeCN**, (b) **$Co_2L^{1,3}$-PPh_3**, (c) **$Co_2L^{1,3}$-DCHIm**, and (d) **$Co_2L^{1,3}$-SPh** in butyronitrile. No Co(II)-based EPR signal was detected for the **$Co_2L^{1,3}$-SPh** complex. Spectra are vertically offset for clarity. For simulation parameters please see Table S8. Experimental conditions: Microwave frequency ca. 9.35 GHz, microwave power: 0.016 mW, temperature: 13.5 K; modulation amplitude: 0.2 mT.

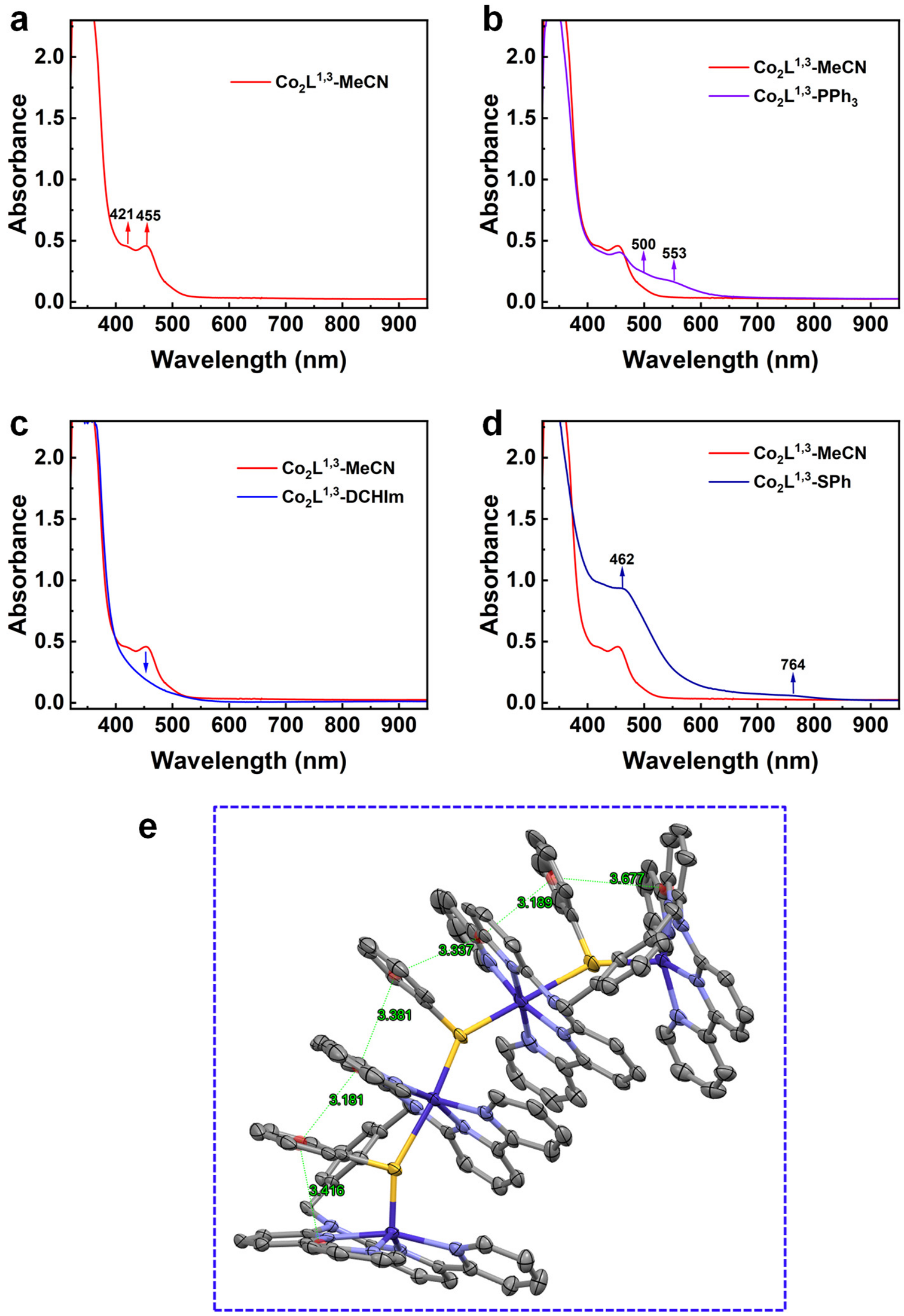

Figure S4. UV-vis spectrum of (a) **$Co_2L^{1,3}$-MeCN** (red), (b) **$Co_2L^{1,3}$-PPh_3** (violet), (c) **$Co_2L^{1,3}$-DCHIm** (blue), and (d) **$Co_2L^{1,3}$-SPh** (dark blue) in acetonitrile. (e) Asymmetric unit of **$Co_2L^{1,3}$-SPh** highlighting π–π stacking interactions between thiophenolate phenyl rings and the bbpya framework. Ring centroids are shown as red spheres, and centroid–centroid distances are indicated by green dashed lines. The distances range from 3.181 to 3.677 Å, with an average of ca. 3.36 Å. Hydrogen atoms, solvent molecules, and noncoordinating counterions are omitted for clarity.

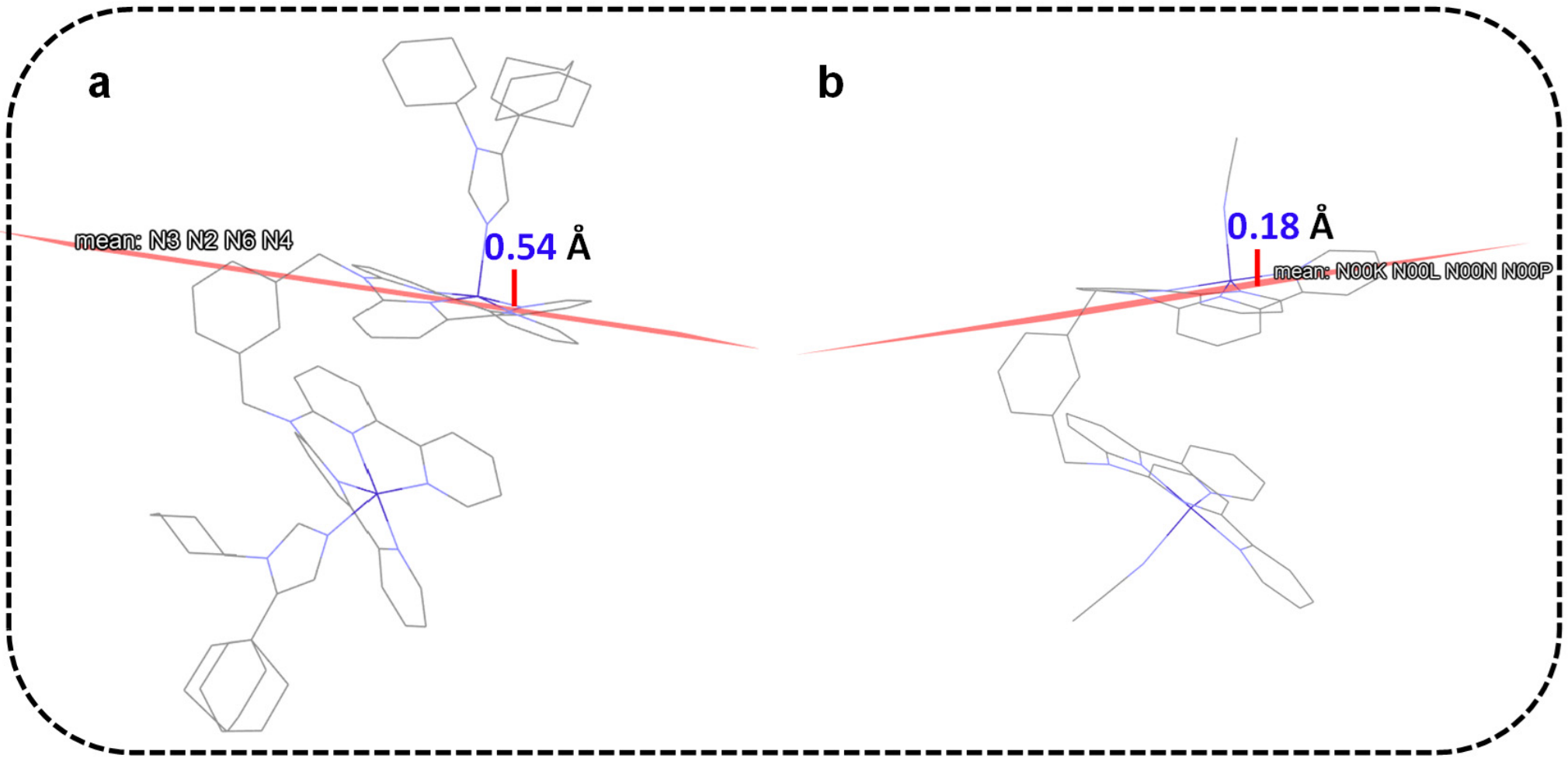

Figure S5. Comparison of the crystallographically determined Co coordination environments in (a) **$Co_2L^{1,3}$-DCHIm** and (b) **$Co_2L^{1,3}$-MeCN**. The red surfaces represent the least-squares planes defined by the four equatorial N-donor atoms. The perpendicular displacement of Co from the N_4 plane increases from 0.18 Å in **$Co_2L^{1,3}$-MeCN** to 0.54 Å in **$Co_2L^{1,3}$-DCHIm**, demonstrating the greater square-pyramidal distortion induced by DCHIm coordination.

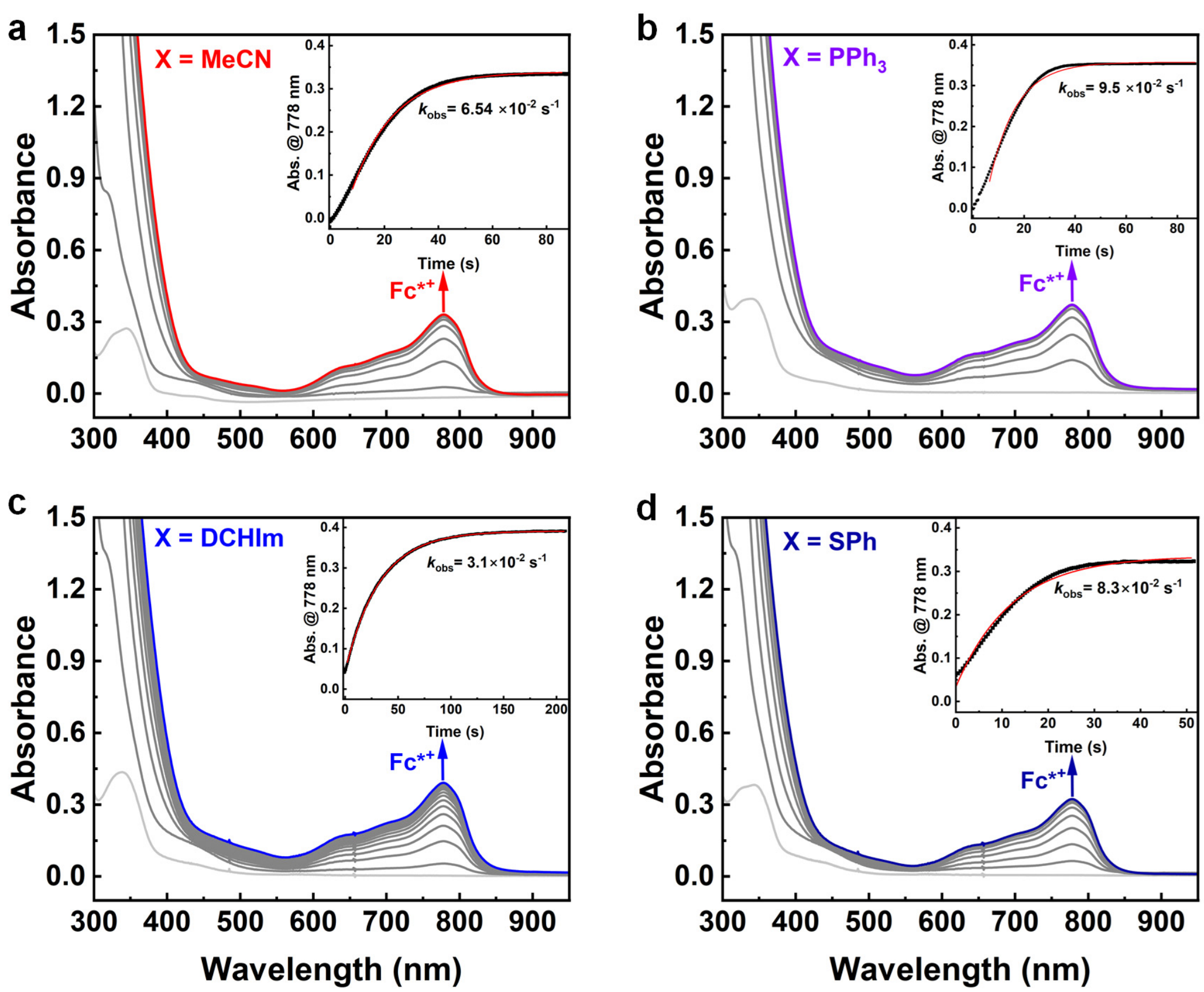

Figure S6. UV–vis spectral changes during the catalytic ORR with **$Co_2L^{1,3}$-X** in air saturated MeCN at 298 K in presence of (a) X = MeCN, (b) PPh_3, (c) DCHIm, (d) PhS^-. Inset shows the time profiles of the Fc^{*+} formation (@778 nm) during the ORR with **$Co_2L^{1,3}$** in presence of different axial ligand. [**$Co_2L^{1,3}$-X**] = 10 μM, [O_2] = 1.7 mM, [Fc*] = 1 mM, and [TFA] = 10 mM.

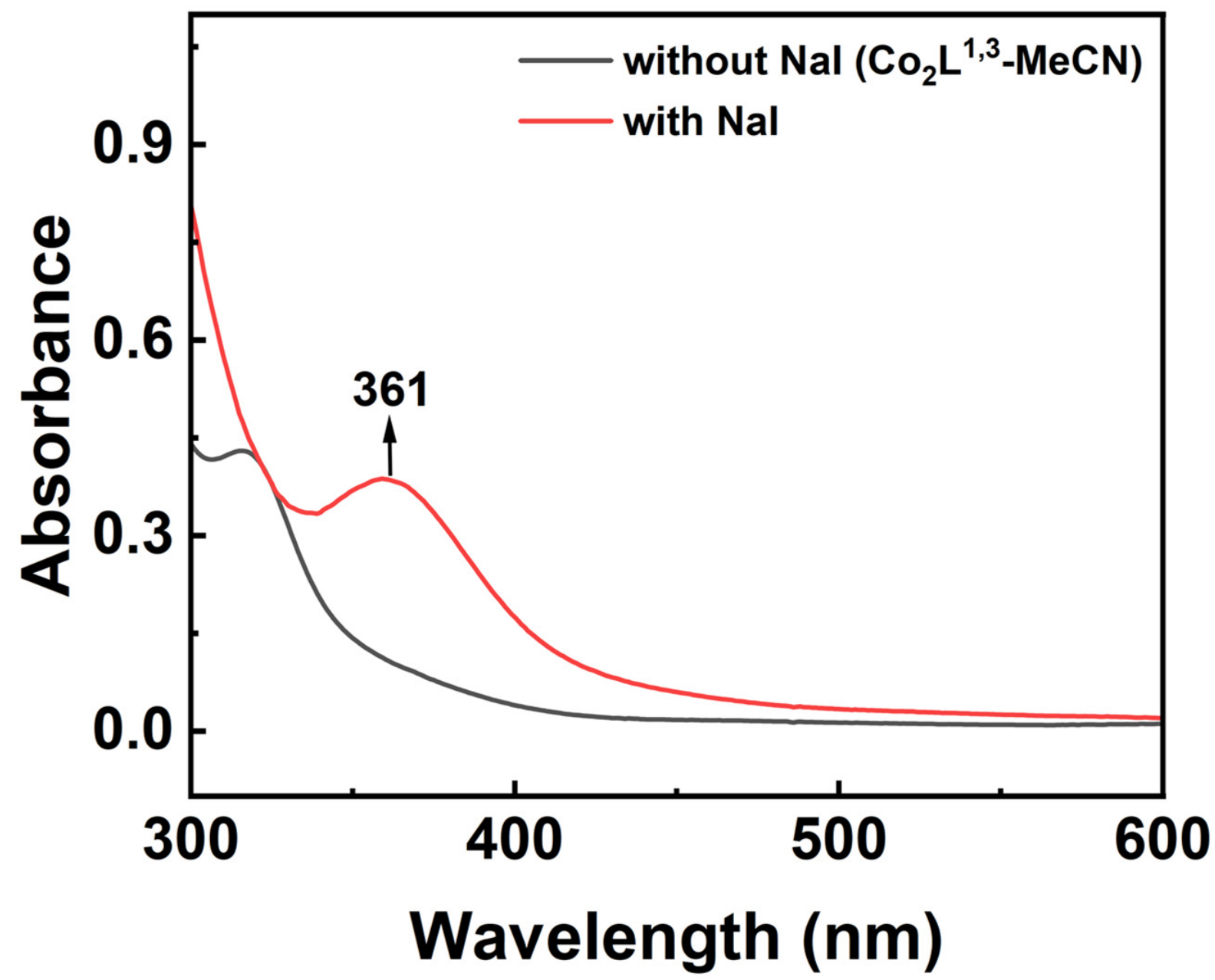

Figure S7. Iodometric titration of the solution after chemical ORR catalysis by diluting the after-reaction solution with MeCN (1/20). The yield of H_2O_2 was determined to be 68%.

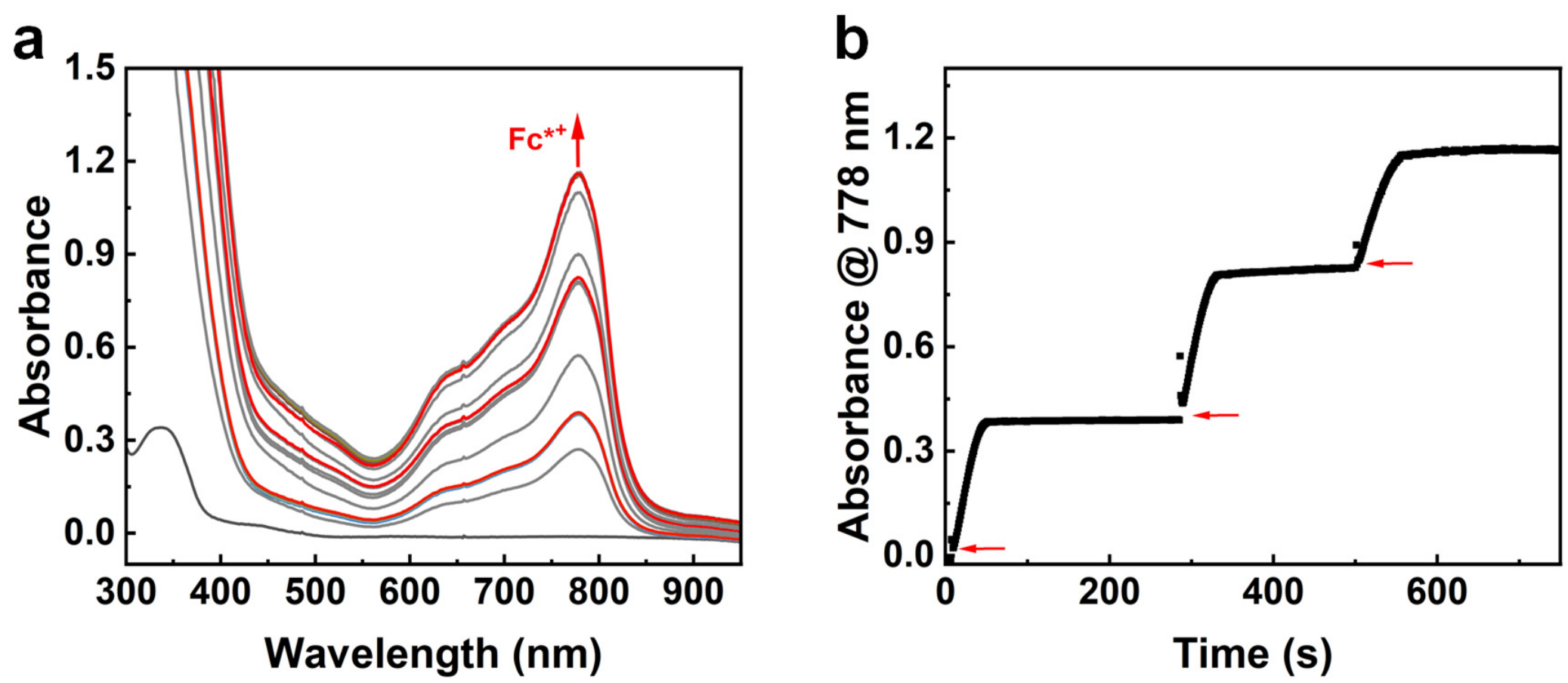

Figure S8. (a) UV–vis spectral changes during the catalytic ORR by $Co_2L^{1,3}$-X in MeCN at 298 K under air-saturated conditions ($[O_2]$ = 1.7 mM; $[Co_2L^{1,3}$-MeCN] = 10 μM; [Fc*] = 1 mM; [TFA] = 10 mM) (b) Time profiles for Fc^{*+} formation (absorbance at 778 nm). 1 mM of Fc* is added at the 5 s , 285 s, and 500 s, respectively (marked as red arrow).

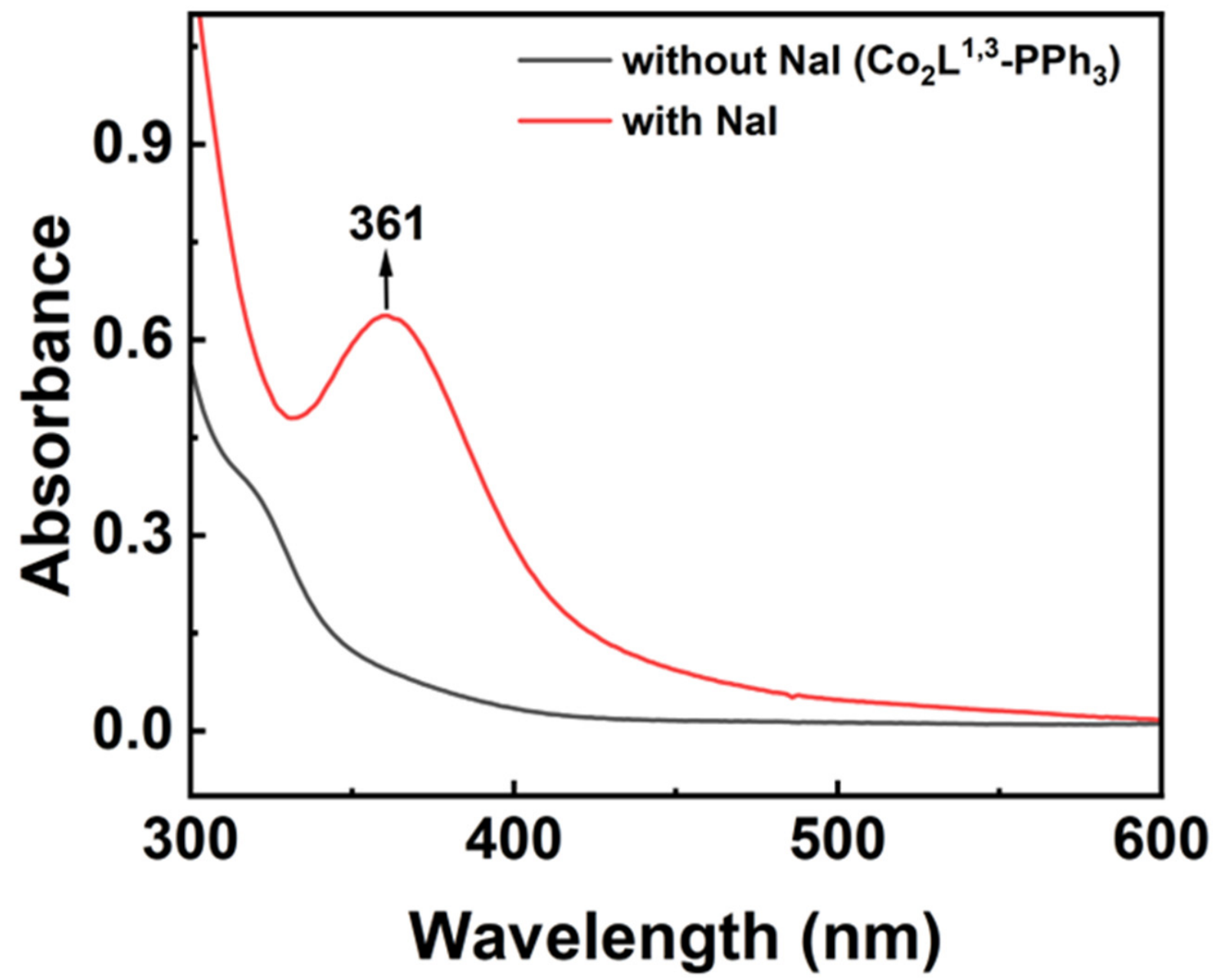

Figure S9. Iodometric titration of the solution after chemical ORR catalysis by diluting the after-reaction solution with MeCN (1/20). The yield of H_2O_2 was determined to be 94%.

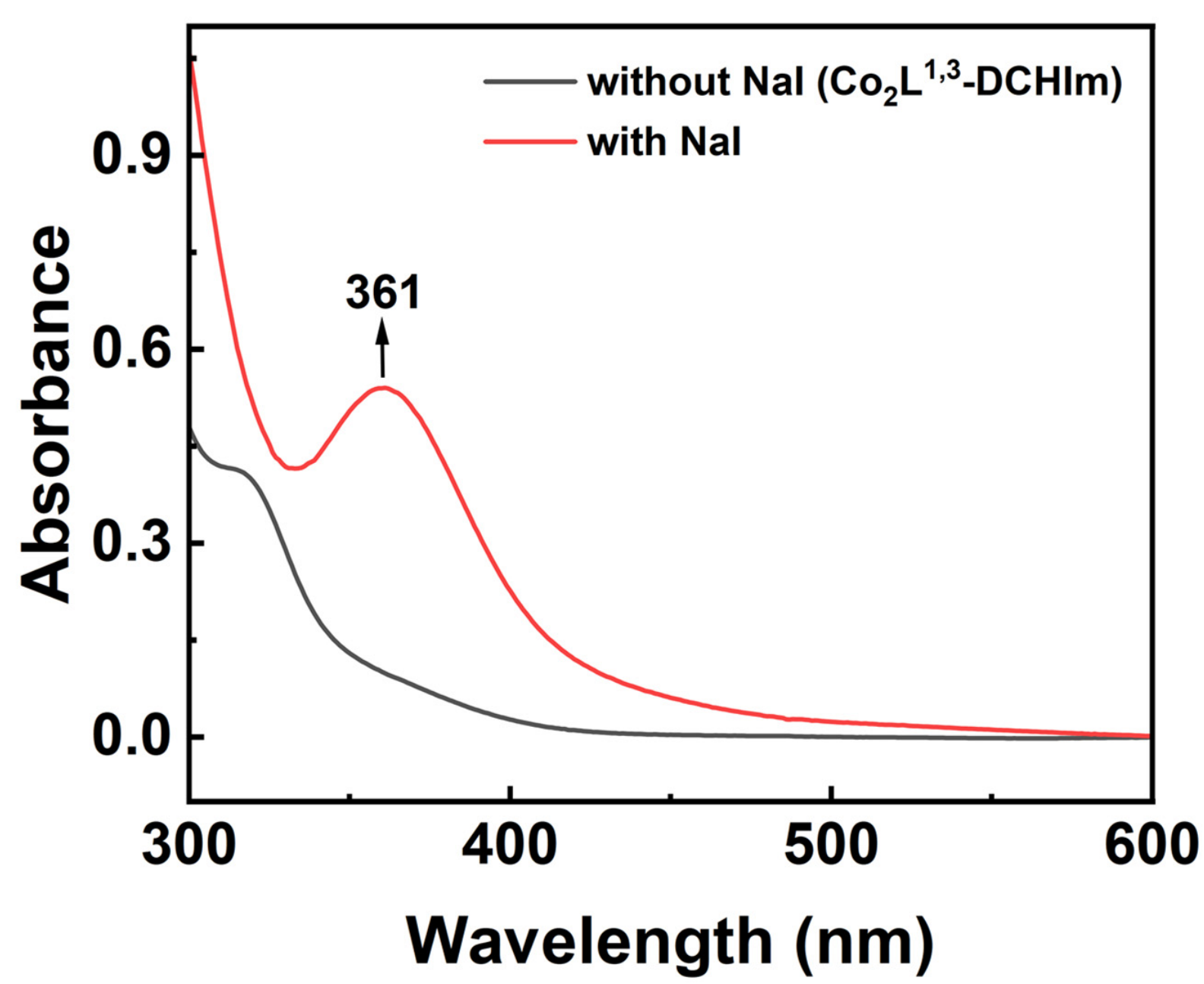

Figure S10. Iodometric titration of the solution after chemical ORR catalysis by diluting the after-reaction solution with MeCN (1/20). The yield of H_2O_2 was determined to be 88%.

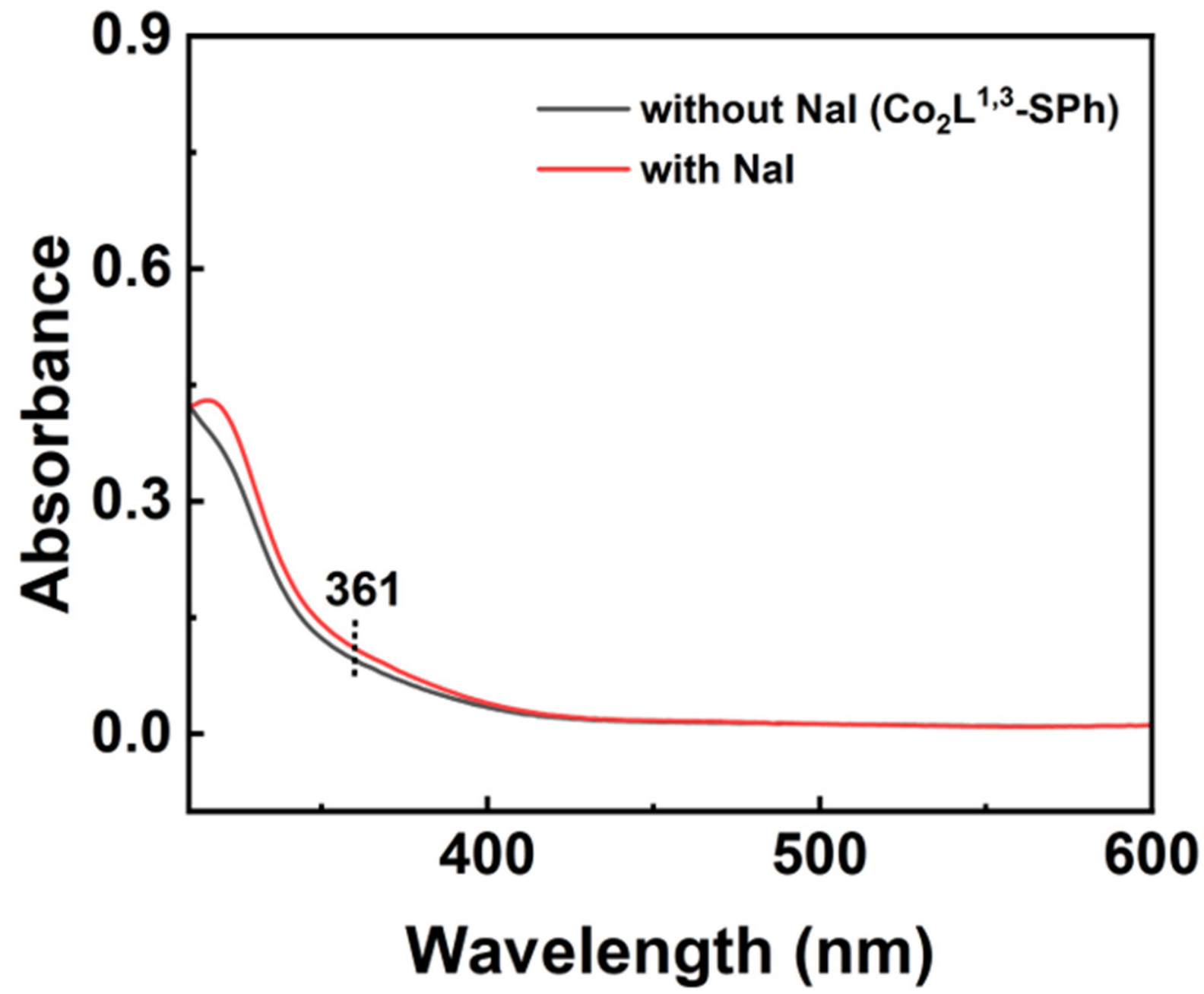

Figure S11. Iodometric titration of the solution after chemical ORR catalysis by diluting the after-reaction solution with MeCN (1/20). No H_2O_2 was detected.

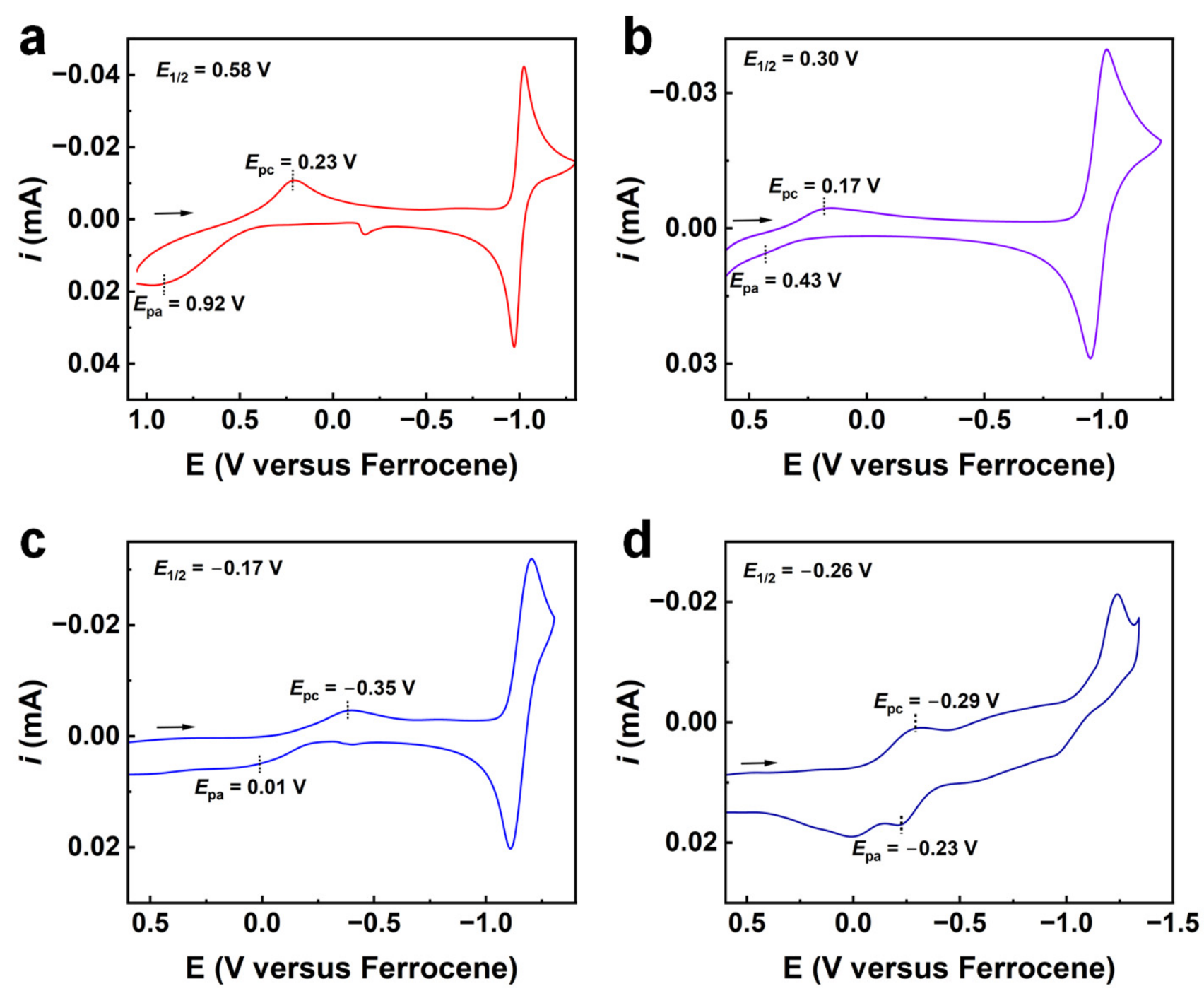

Figure S12. Cyclic voltammograms (CVs) of (a) **$Co_2L^{1,3}$-MeCN** (red), (b) **$Co_2L^{1,3}$-PPh$_3$** (purple), (c) **$Co_2L^{1,3}$-DCHIm** (blue), and (d) **$Co_2L^{1,3}$-SPh** (navy) under Ar in MeCN. The scans were initiated at +1 V for $Co_2L^{1,3}$-MeCN and at +0.6 V vs Fc^+/Fc for other complexes and swept cathodically (arrow). Apparent $E_{1/2}$ values are +0.58, +0.30, −0.17 V and −0.26 V for the MeCN, PPh_3, DCHIm, and PhS

complexes, respectively. The additional Epa around 0.0 V in **$Co_2L^{1,3}$-SPh** is tentatively assigned to the oxidation of PhS^-.

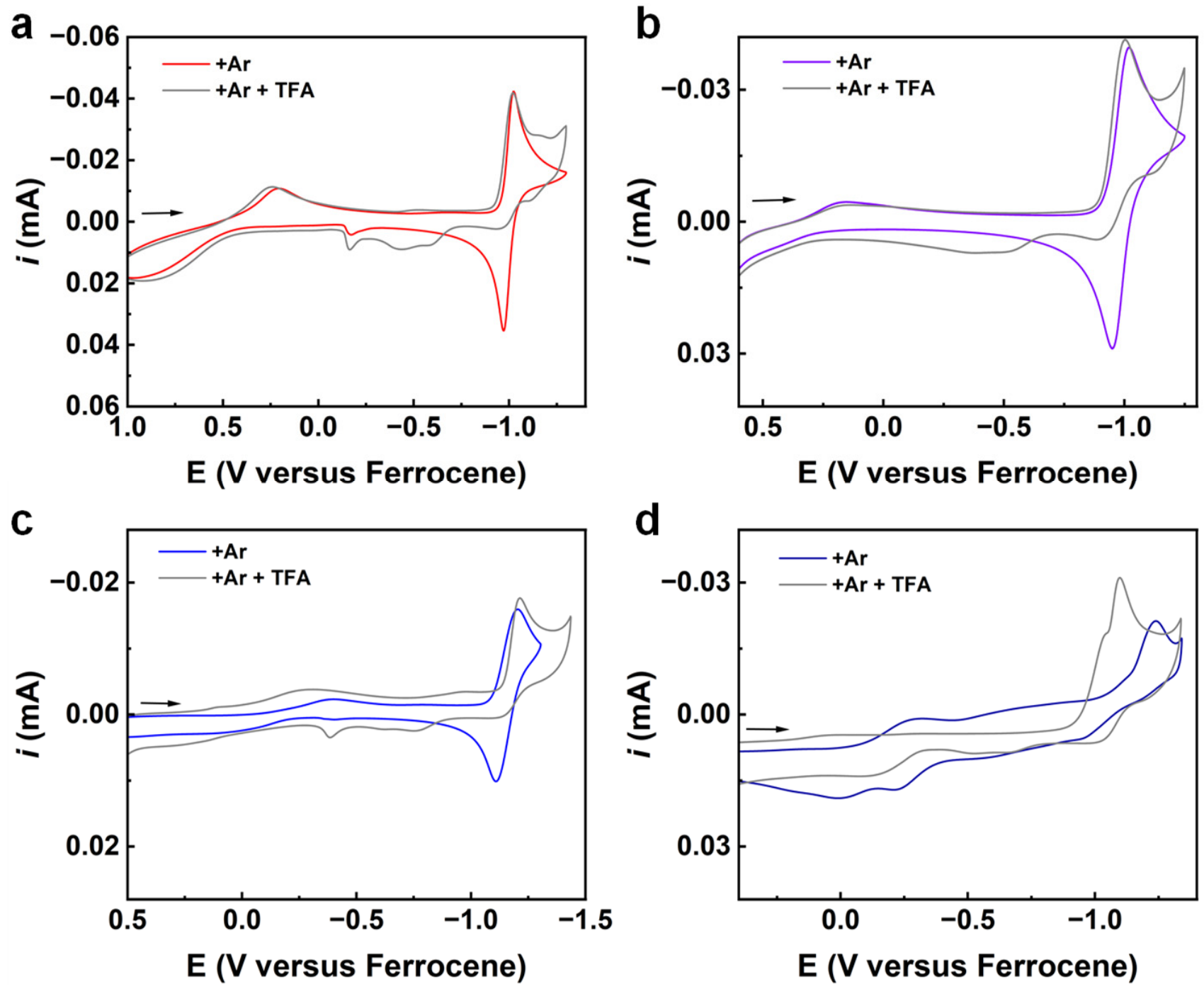

Figure S13. CVs of (a) $Co_2L^{1,3}$-MeCN, (b) $Co_2L^{1,3}$-PPh_3, (c) $Co_2L^{1,3}$-DCHIm, and (d) $Co_2L^{1,3}$-SPh under Ar in the absence (coloured traces) and presence (grey traces) of 20 mM TFA in MeCN. The scans were initiated at +1 V for $Co_2L^{1,3}$-MeCN and at +0.6 V vs Fc^+/Fc for other complexes and swept cathodically (arrow).

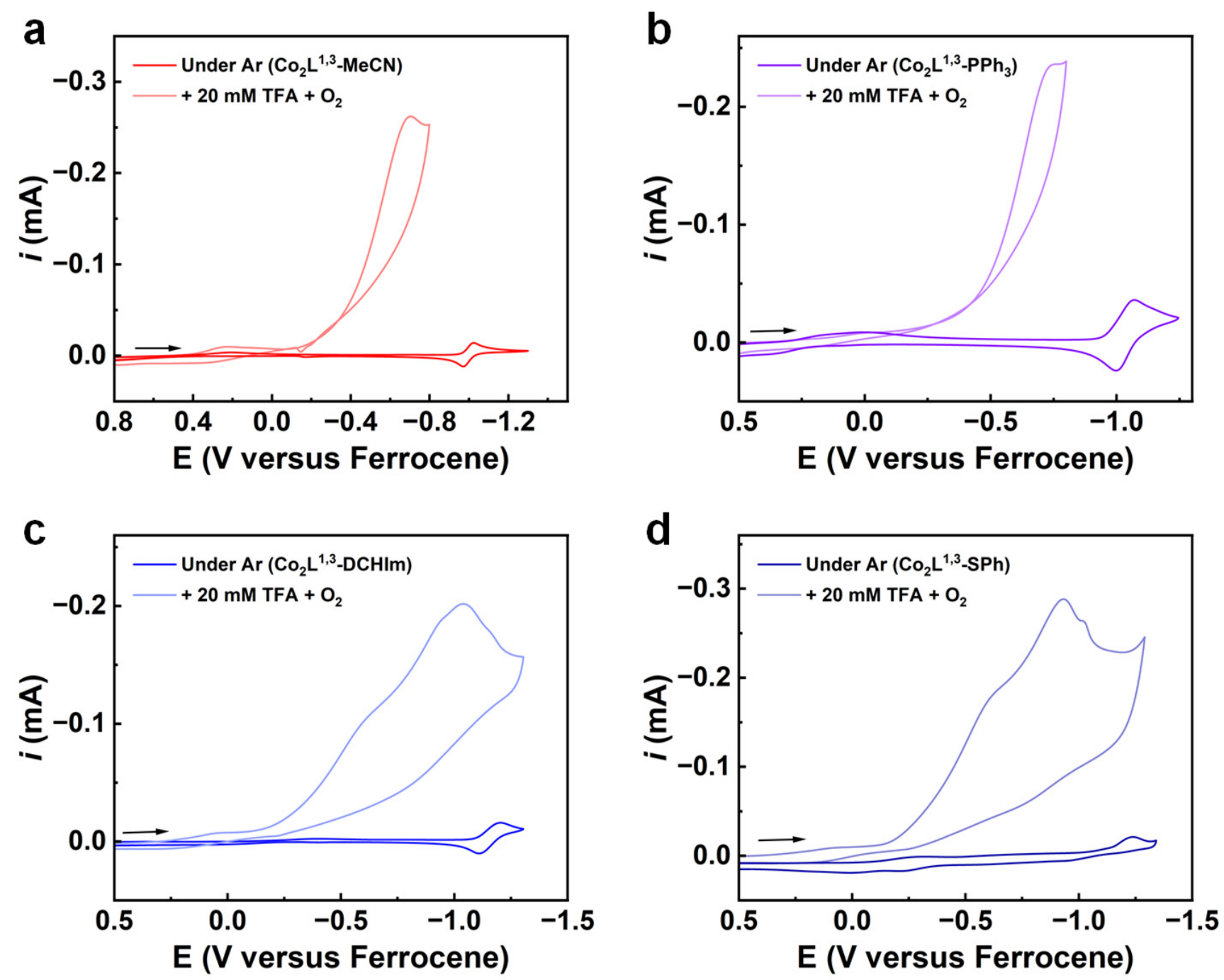

Figure S14. CV in presence of 20 mM TFA in Ar-saturated and in O_2 saturated MeCN at 298 K for (a) **$Co_2L^{1,3}$-MeCN**, (b) **$Co_2L^{1,3}$-PPh_3**, (c) **$Co_2L^{1,3}$-DCHIm**, and (d) **$Co_2L^{1,3}$-SPh**. The scans were initiated at +0.8 V for **$Co_2L^{1,3}$-MeCN** and at +0.5 V vs Fc^+/Fc for other complexes and swept cathodically (arrow).

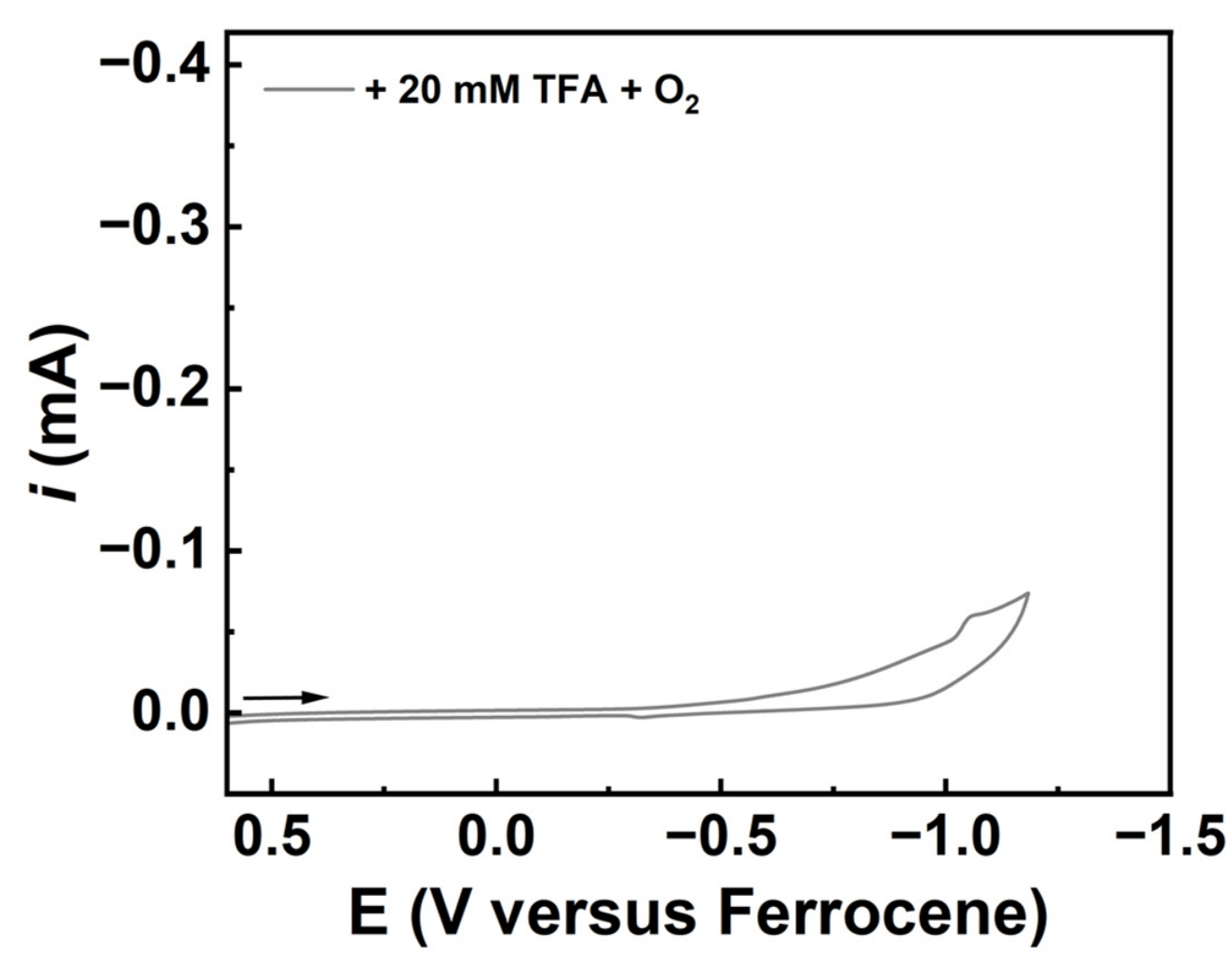

Figure S15. CV of 20 mM TFA in O_2 saturated MeCN at 298 K in the absence of any cobalt catalysts. Scan rate: 100 mV s^{-1}; scans were initiated at +0.6 V versus Fc^+/Fc and swept cathodically.

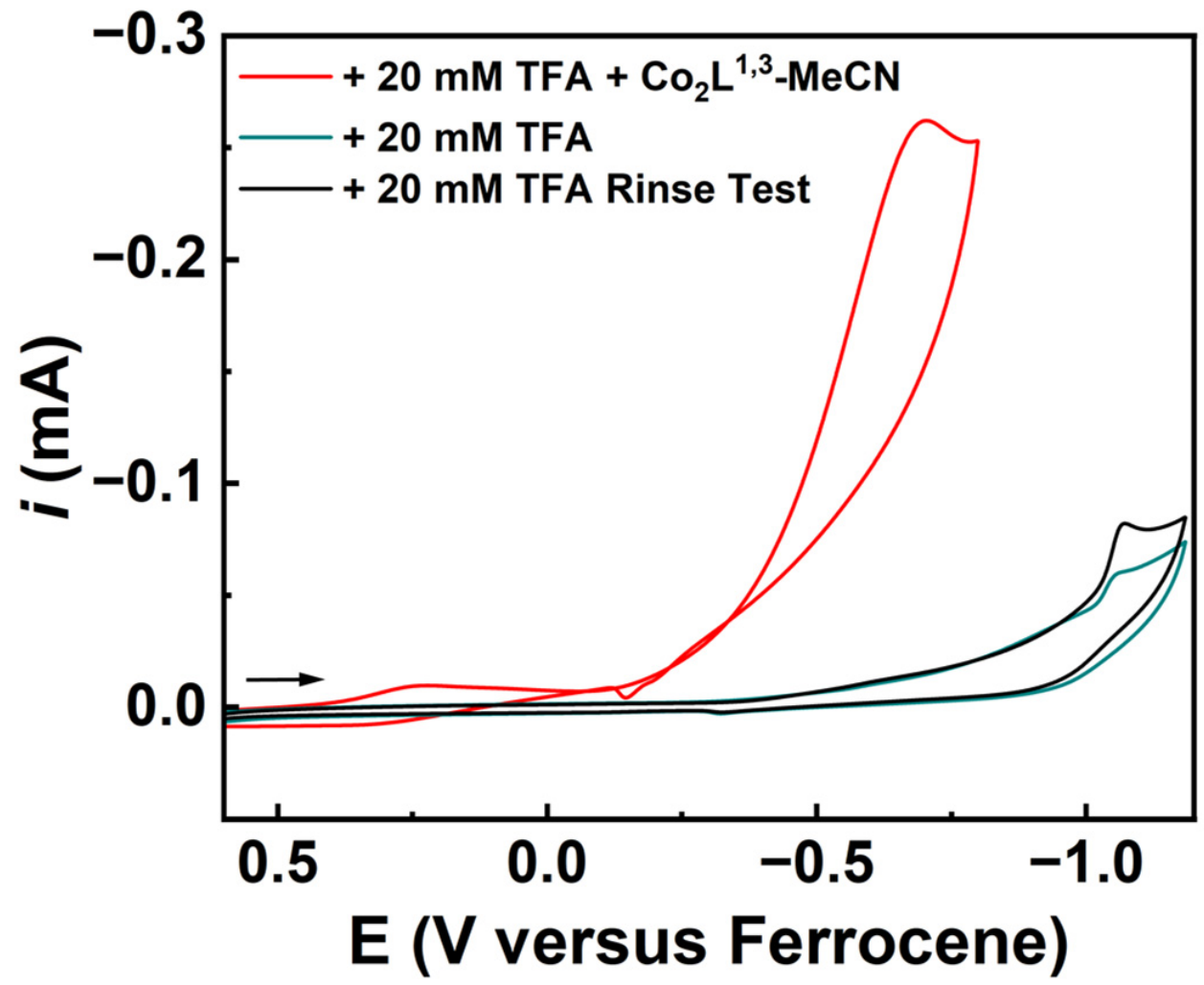

Figure S16. CV rinse test in O_2-saturated MeCN containing TFA (20 mM): **$Co_2L^{1,3}$-MeCN** (0.5 mM) under catalytic conditions (red); bare GC electrode in catalyst-free electrolyte (cyan); and GC electrode previously cycled under catalytic conditions, rinsed thoroughly with MeCN, and transferred to fresh catalyst-free electrolyte (black). The near-overlap of the black and cyan traces indicates no persistent catalytically active deposit. Scan rate: 100 mV s^{-1}; scans were initiated at +0.6 V versus Fc^+/Fc and swept cathodically (arrow).

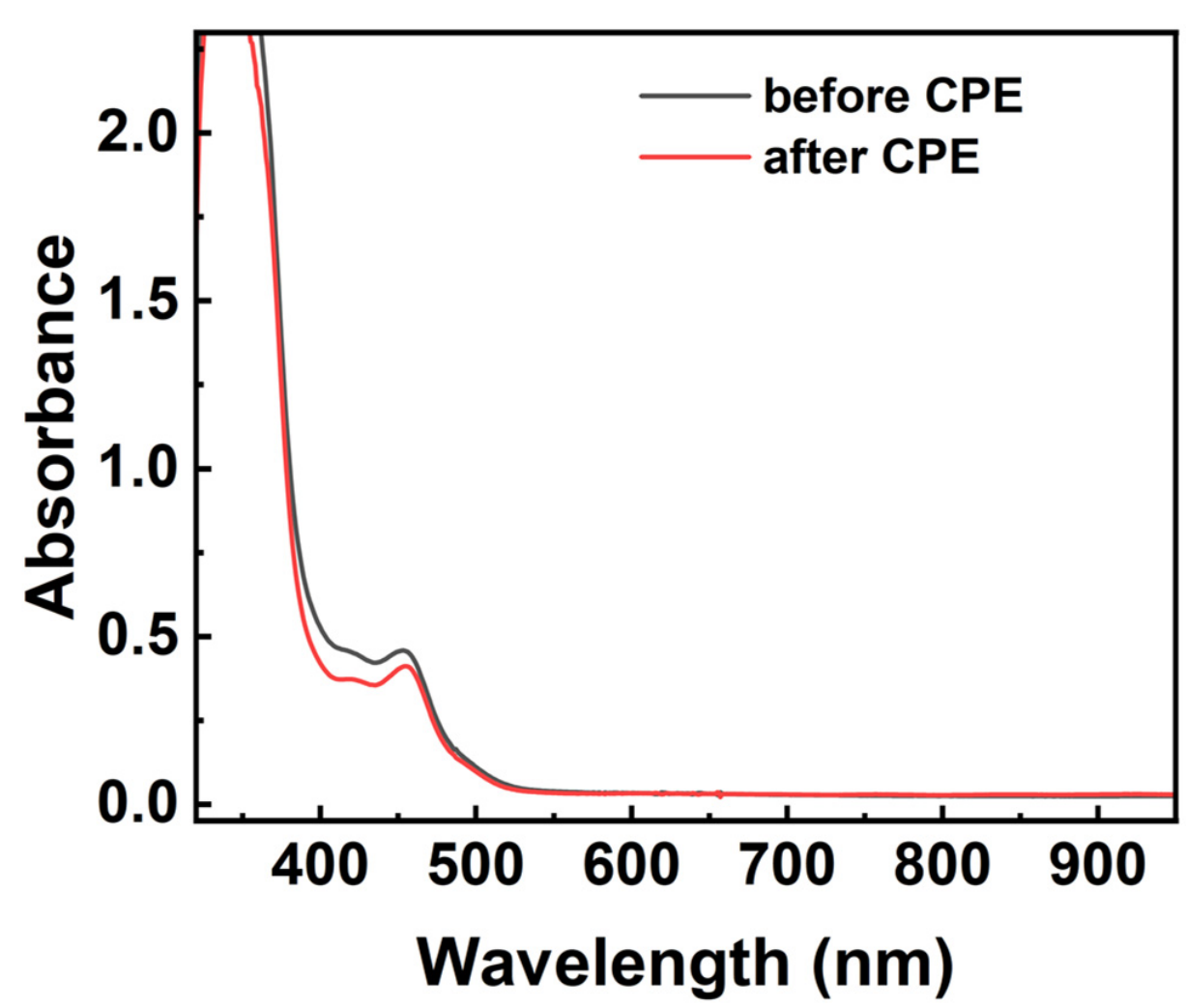

Figure S17. UV-vis spectra of the **$Co_2L^{1,3}$-MeCN** complex-containing electrolyte (200 µL of 1 mM Co(II)-containing electrolyte + 800 µL electrolyte) before CPE (black) and after CPE (red). CPE conditions: applied potential −0.3 V vs Fc^+/Fc; 1 mM $Co_2L^{1,3}$-MeCN, 20 mM TFA, O_2-saturated MeCN. 82% of the catalysts remaining after CPE.

Rotating-ring disk electrode (RRDE) Measurements:

Determination of the RRDE collection efficiency.

The empirical collection efficiency of the rotating ring-disk electrode (RRDE) was determined using ferrocene. Experiments were performed using a Metrohm RRDE setup equipped with a glassy carbon disk electrode (5 mm diameter) and a concentric Pt ring electrode. A Pt wire and an Ag/AgCl pseudoreference electrode were used as the counter and reference electrodes, respectively. An argon-saturated MeCN solution (50 mL) containing $TBAPF_6$ (0.1 M) was first prepared. Prior to the addition of ferrocene, a background cyclic voltammogram was recorded to confirm the absence of residual redox-active impurities or dissolved O_2. Ferrocene was then added to give a final concentration of 0.5 mM, and a cyclic voltammogram was recorded to determine the appropriate potential window for Fc/Fc^+ oxidation. Based on this measurement, the disk potential was swept from 0 to 0.8 V vs Ag/AgCl at a scan rate of 0.02 V s^{-1}, while the Pt ring electrode was held at 0 V vs Ag/AgCl to reduce the ferrocenium generated at the disk.

Linear sweep voltammograms were then recorded at rotation rates of 200, 600, 1000, 1400, 1800, and 2200 rpm. At each rotation rate, the background-corrected limiting disk current for ferrocene oxidation and the corresponding ring current for ferrocenium reduction were used to calculate the empirical collection efficiency according to:

$$N_{\mathrm{empirical}} = \left|\frac{i_{\mathrm{ring}}}{i_{\mathrm{disk}}}\right| \qquad (13)$$

Where, i_{ring} is the corresponding ring current and i_{disk} is the disk current. The collection efficiency at each rotation rate was obtained by multiplying $N_{\mathrm{emperical}}$ by 100. The average experimental collection efficiency was approximately 26%. This experimentally determined collection efficiency was used for all subsequent RRDE product-quantification experiments.

RRDE experiments: All measurements were carried out in MeCN (50 mL) containing $TBAPF_6$ (0.1 M) as the supporting electrolyte and catalyst (0.2 mM) at a scan rate of 0.03 V s^{-1}. For measurements under argon, the electrolyte solution was first sparged with Ar until saturation was achieved. A background CV was recorded to confirm the absence of residual O_2 or other redox-active impurities. The catalyst (0.25 mM) and the requisite amount of additional axial ligand was then dissolved in the solution, followed by addition of 20 equiv. TFA. A standard CV was recorded to confirm the appropriate potential window for the RRDE experiment. Based on this measurement, the disk potential was scanned from 0.4 to −1.2 V vs Fc^+/Fc, while the Pt ring electrode was held at 1 V vs Fc^+/Fc. Linear sweep voltammograms (LSV) were recorded at rotation rates 1600 rpm. The same procedure was then repeated under O_2-saturated conditions. O_2 saturation was achieved by sparging the solution with O_2 for approximately 15 min prior to the measurement and maintaining an O_2 atmosphere over the solution during the experiment.

The arithmetic mean of the number of electrons received by O_2 (n_{RRDE}) during the ORR was calculated from the disk current (i_{disk}) and ring current (i_{ring}) according to equation 8.

$$n_{\mathrm{RRDE}} = \frac{4 \times i_{\mathrm{disk}}}{i_{\mathrm{disk}} + (i_{\mathrm{ring}}/N_{H_2O_2})} \qquad (14)$$

The H_2O_2 ratio (p) is defined as the fraction of O_2 reduced to H_2O_2 and related to n_{RRDE} by equation 15.

$$n_{\mathrm{RRDE}} = 4 - 2p \qquad (15)$$

Multiplying p by 100 provides the % H_2O_2 selectivity of the ORR.

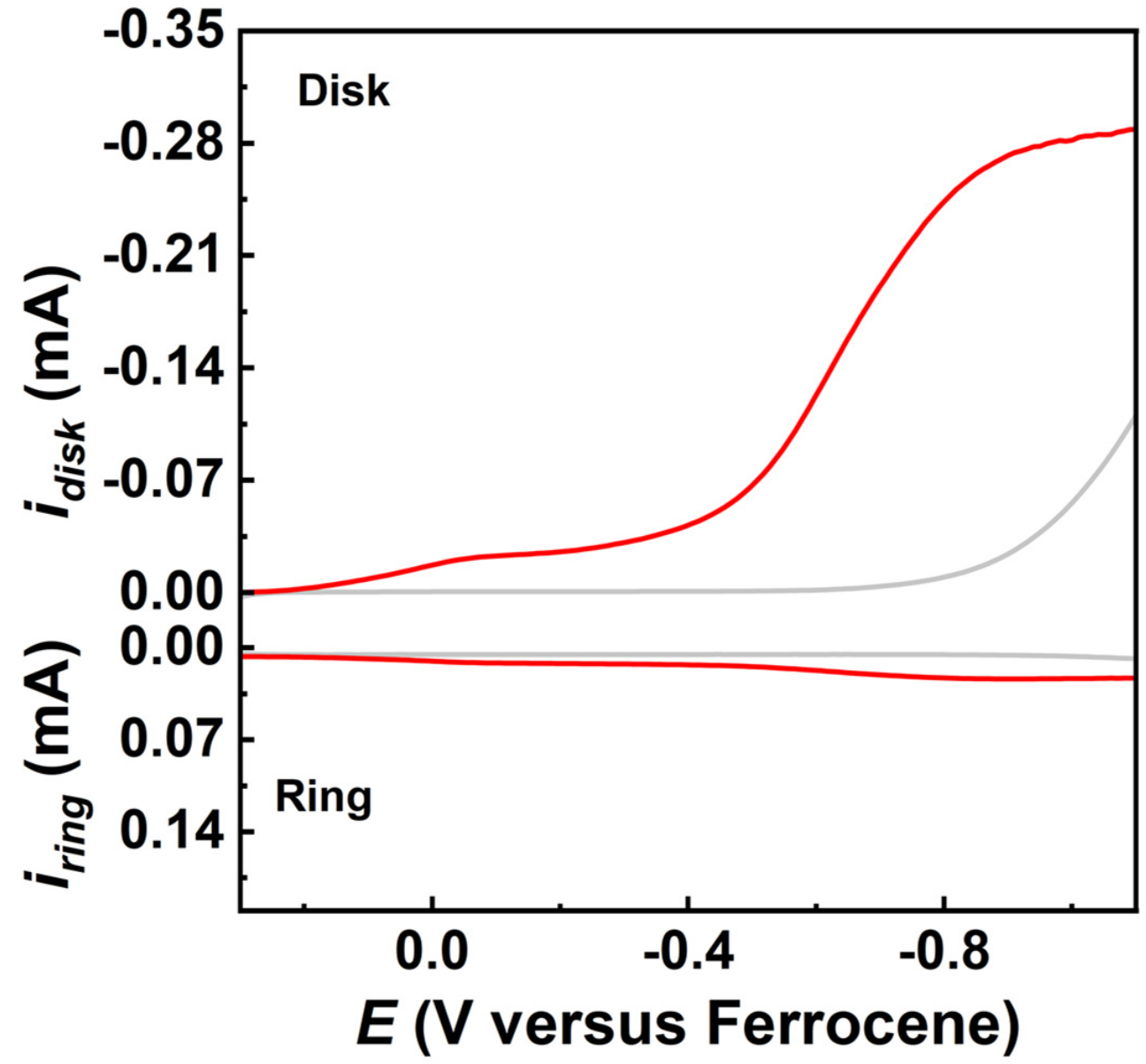

Figure S18. Linear sweep voltammograms of the RRDE experiment under O_2-saturated atmosphere of a 20 equiv. TFA in the absence (gray lines) or presence (red lines) of 0.2 mM $\mathbf{Co_2L^{1,3}}$**-MeCN**. Scan rate: 30 mV.s^{-1}; rotation rate: 1600 rpm, ring current measured with the Pt ring held at +1 V vs Fc^+/Fc.

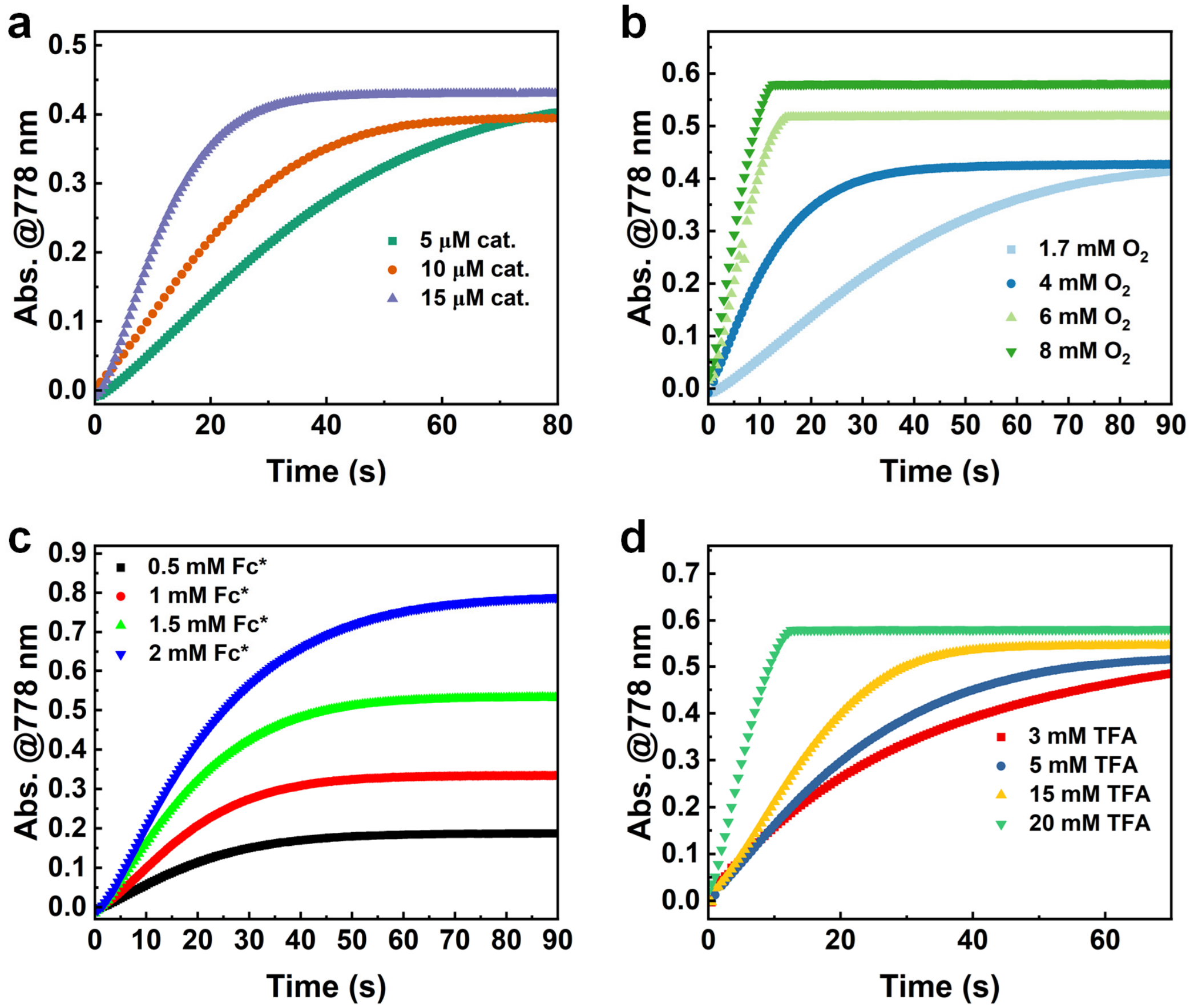

Figure S19. (a) Time traces of absorbance at 778 nm due to the formation of Fc^{*+} for the reaction of **$Co_2L^{1,3}$-MeCN** (5-15 μM) with O_2 (air-saturated) by Fc* (1 mM) in presence of TFA (10 mM) in MeCN. (b) Time traces of absorbance at 778 nm due to the formation of Fc^{*+} for the reaction of **$Co_2L^{1,3}$-MeCN** (10 μM) with O_2 (1.7 – 8 mM) by Fc* (1 mM) in presence of TFA (10 mM) in MeCN. (c) Time traces of absorbance at 778 nm due to the formation of Fc^{*+} for the reaction of **$Co_2L^{1,3}$-MeCN** (10 μM) with O_2 (air-saturated) by Fc* (0.5 - 2 mM) in presence of TFA (10 mM) in MeCN. (d) Time traces of absorbance at 778 nm due to the formation of Fc^{*+} for the reaction of **$Co_2L^{1,3}$-MeCN** (10 μM) with O_2 (air-saturated) by Fc* (1 mM) in presence of TFA (3 - 20 mM) in MeCN.

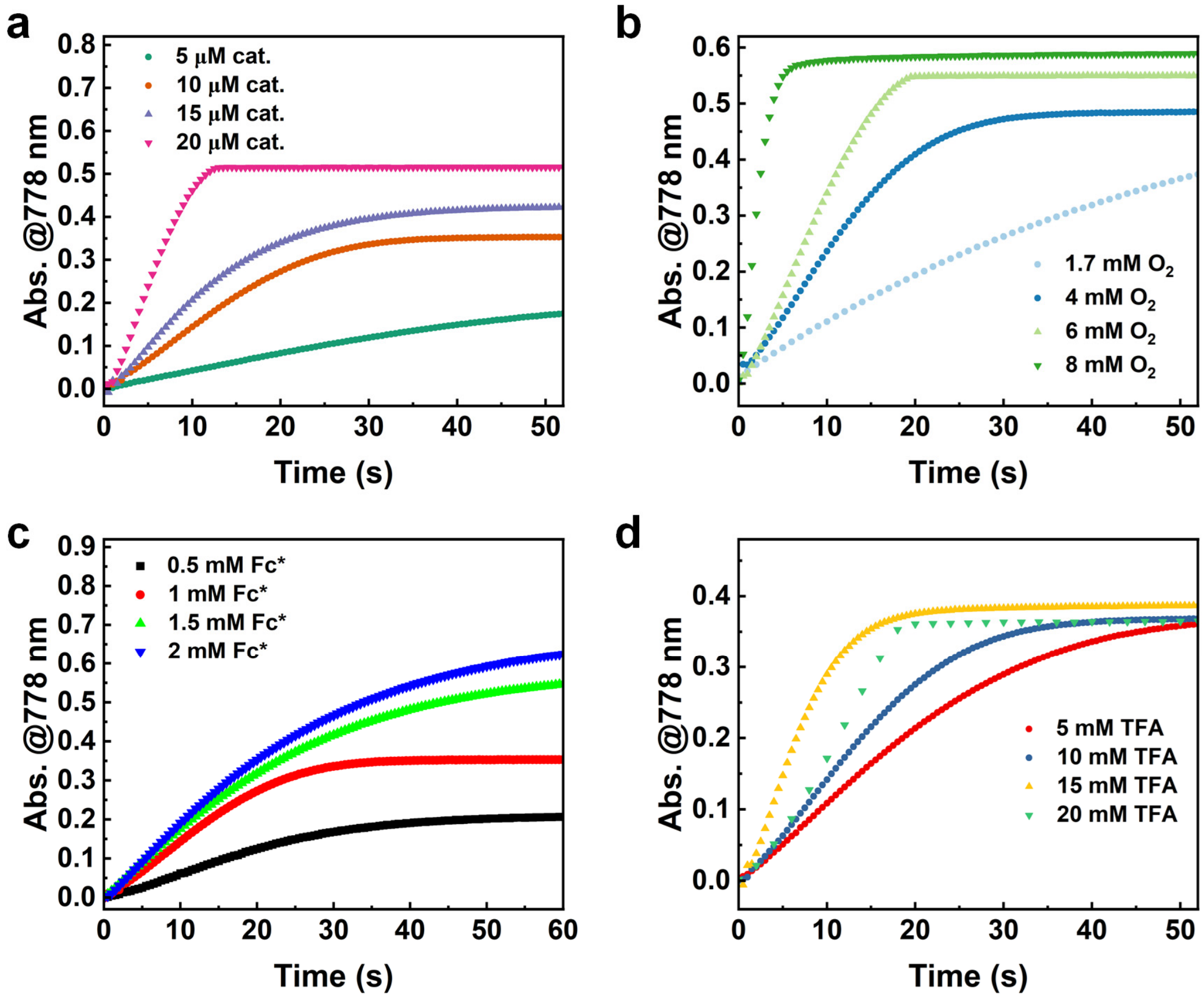

Figure S20. (a) Time traces of absorbance at 778 nm due to the formation of Fc^{*+} for the reaction of $\mathbf{Co_2L^{1,3}}$**-PPh$_3$** (5-15 μM) with O_2 (air-saturated) by Fc* (1 mM) in presence of TFA (10 mM) in MeCN. (b) Time traces of absorbance at 778 nm due to the formation of Fc^{*+} for the reaction of $\mathbf{Co_2L^{1,3}}$**-PPh$_3$** (10 μM) with O_2 (1.7 – 8 mM) by Fc* (1 mM) in presence of TFA (10 mM) in MeCN. (c) Time traces of absorbance at 778 nm due to the formation of Fc^{*+} for the reaction of $\mathbf{Co_2L^{1,3}}$**-PPh$_3$** (10 μM) with O_2 (air-saturated) by Fc* (0.5 - 2 mM) in presence of TFA (10 mM) in MeCN. (d) Time traces of absorbance at 778 nm due to the formation of Fc^{*+} for the reaction of $\mathbf{Co_2L^{1,3}}$**-PPh$_3$** (10 μM) with O_2 (air-saturated) by Fc* (1 mM) in presence of TFA (3 - 20 mM) in MeCN.

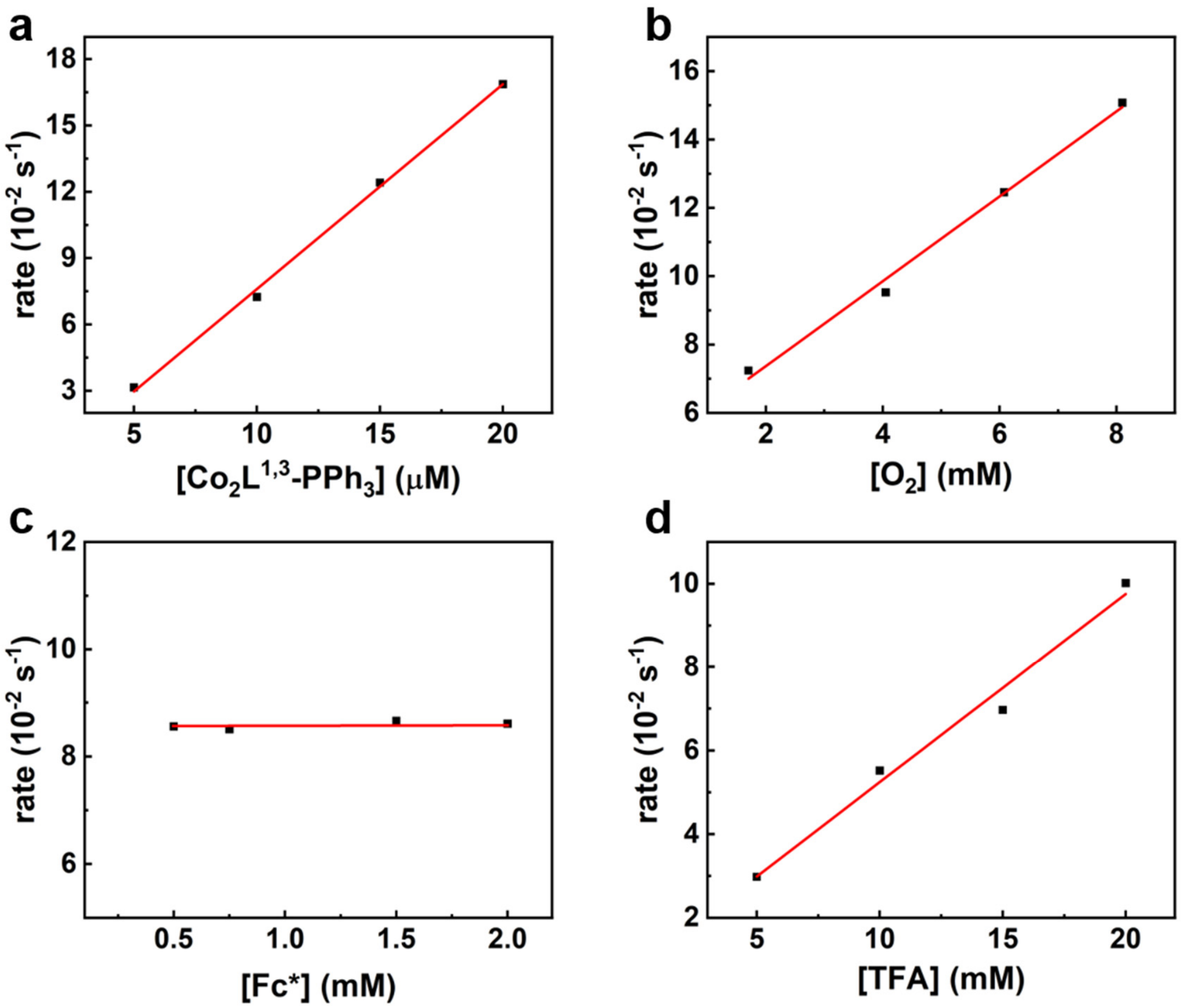

Figure S21. Plots of catalytic ORR rates of **$Co_2L^{1,3}$-PPh_3** vs (a) [**$Co_2L^{1,3}$-PPh_3**] (5-20 μM), (b) [O_2] (1.7-8 mM), (c) [Fc*] (0.5-2 mM), and (d) [TFA] (5-20 mM).

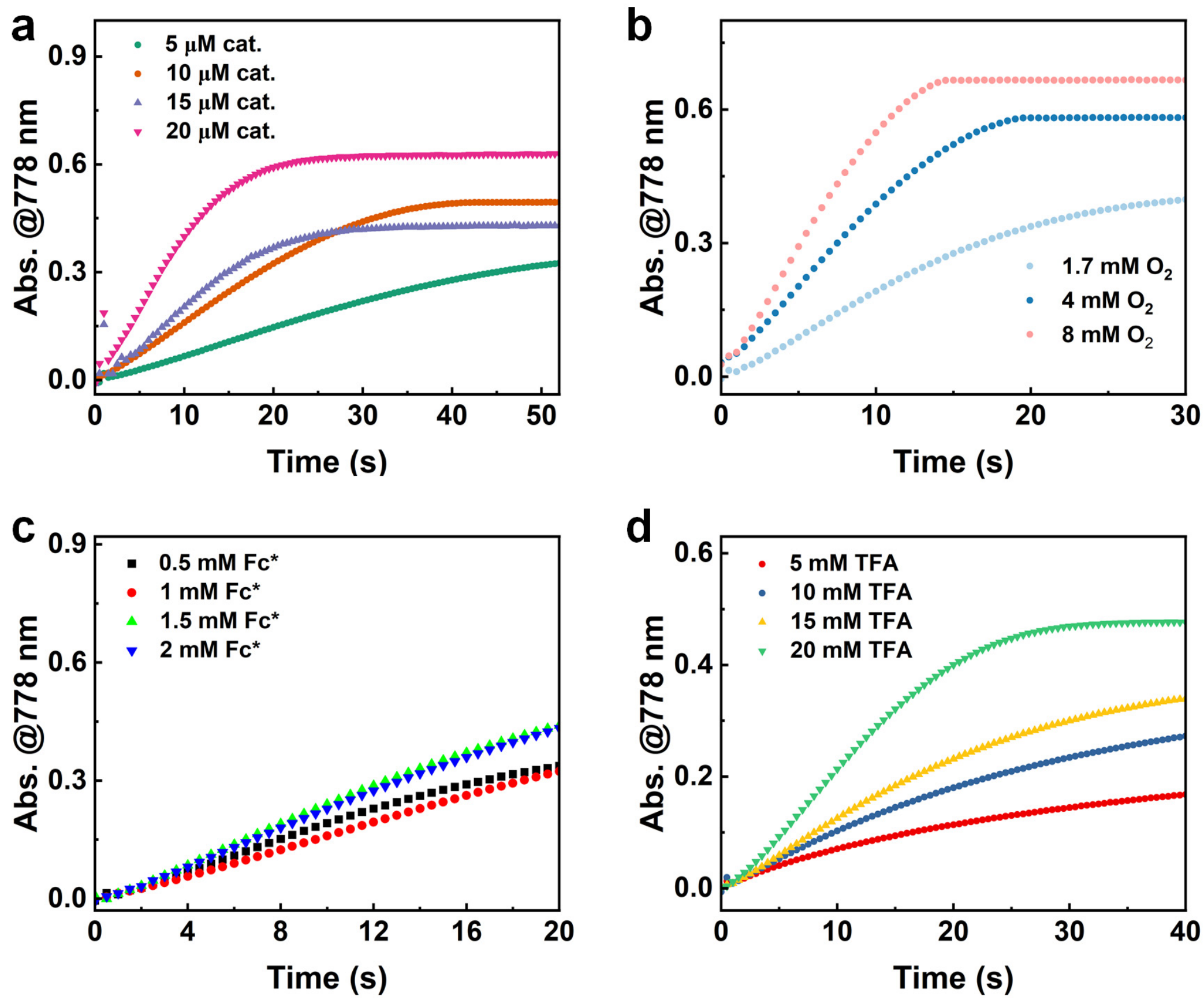

Figure S22. (a) Time traces of absorbance at 778 nm due to the formation of Fc^{*+} for the reaction of **$Co_2L^{1,3}$-DCHIm** (5-20 μM) with O_2 (air-saturated) by Fc* (1 mM) in presence of TFA (10 mM) in MeCN. (b) Time traces of absorbance at 778 nm due to the formation of Fc^{*+} for the reaction of **$Co_2L^{1,3}$-DCHIm** (10 μM) with O_2 (1.7 – 8 mM) by Fc* (1 mM) in presence of TFA (10 mM) in MeCN. (c) Time traces of absorbance at 778 nm due to the formation of Fc^{*+} for the reaction of **$Co_2L^{1,3}$-DCHIm** (10 μM) with O_2 (air-saturated) by Fc* (0.5 - 2 mM) in presence of TFA (10 mM) in MeCN. (d) Time traces of absorbance at 778 nm due to the formation of Fc^{*+} for the reaction of **$Co_2L^{1,3}$-DCHIm** (10 μM) with O_2 (air-saturated) by Fc* (1 mM) in presence of TFA (3 - 20 mM) in MeCN.

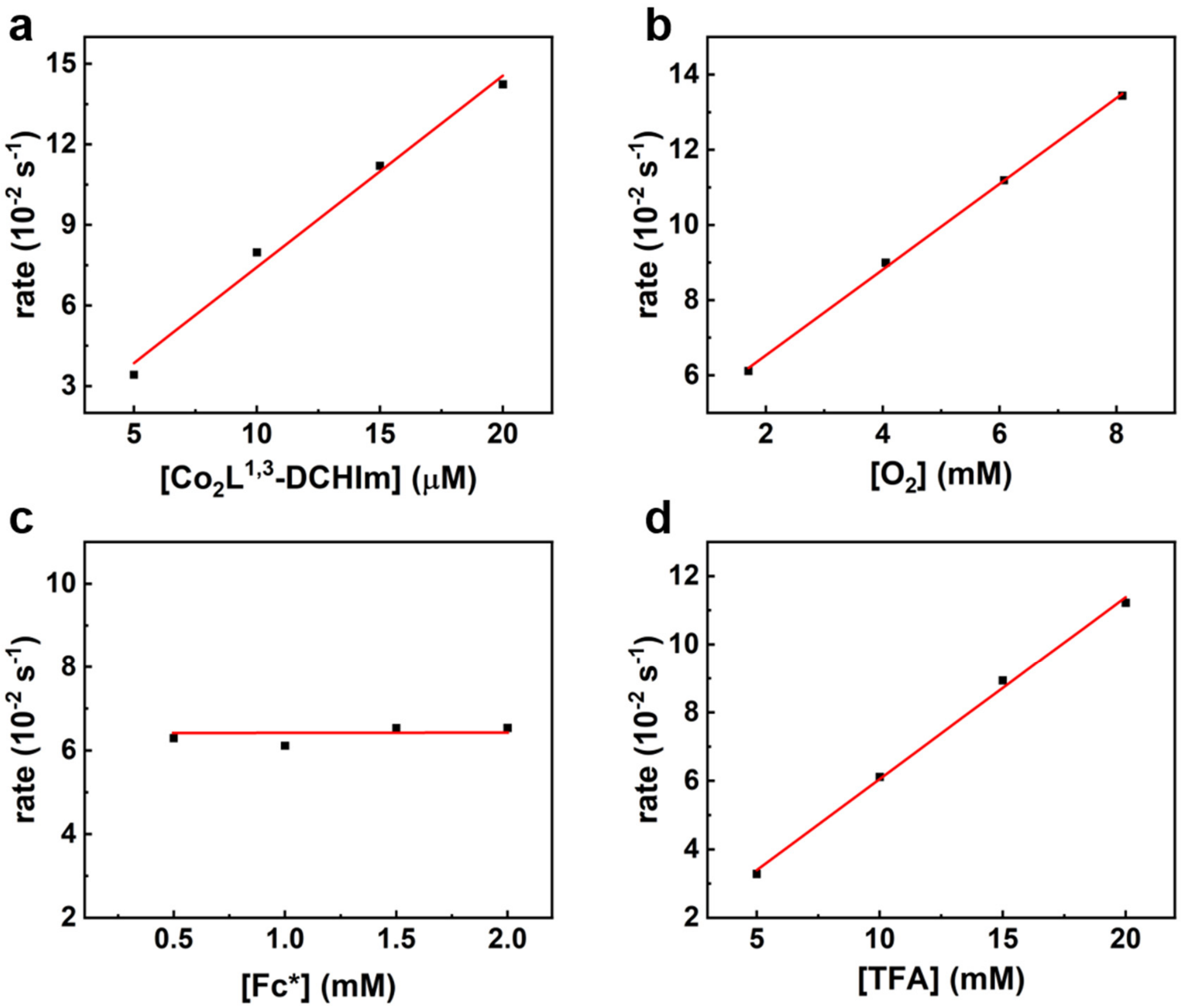

Figure S23. Plots of catalytic ORR rates of **$Co_2L^{1,3}$-DCHIm** vs (a) [**$Co_2L^{1,3}$-DCHIm**] (5-20 μM), (b) [O_2] (1.7-8 mM), (c) [Fc*] (0.5-2 mM), and (d) [TFA] (5-20 mM).

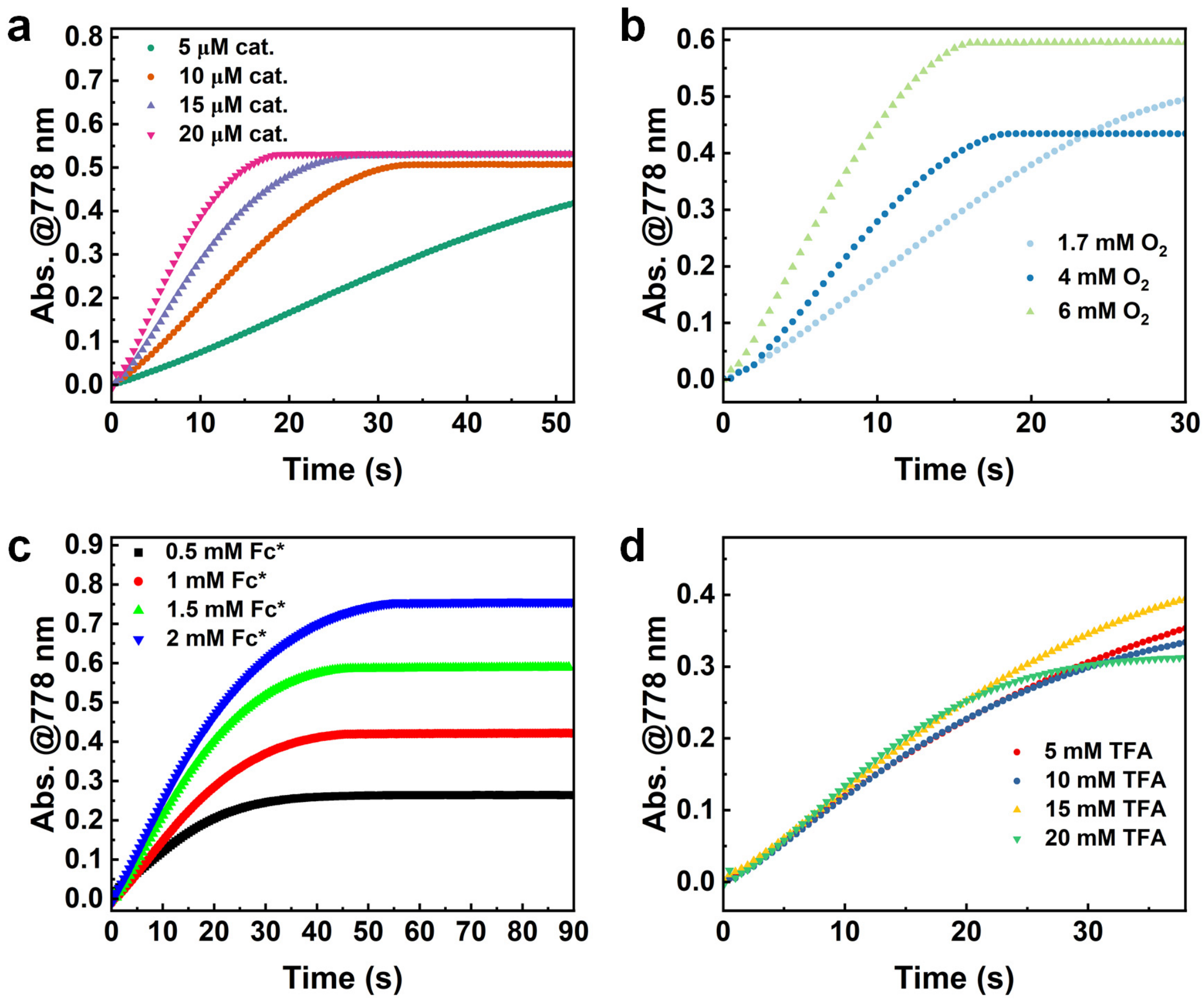

Figure S24. (a) Time traces of absorbance at 778 nm due to the formation of Fc^{*+} for the reaction of **$Co_2L^{1,3}$-SPh** (5-20 μM) with O_2 (air-saturated) by Fc* (1 mM) in presence of TFA (10 mM) in MeCN. (b) Time traces of absorbance at 778 nm due to the formation of Fc^{*+} for the reaction of **$Co_2L^{1,3}$-SPh** (10 μM) with O_2 (1.7 – 8 mM) by Fc* (1 mM) in presence of TFA (10 mM) in MeCN. (c) Time traces of absorbance at 778 nm due to the formation of Fc^{*+} for the reaction of **$Co_2L^{1,3}$-SPh** (10 μM) with O_2 (air-saturated) by Fc* (0.5 - 2 mM) in presence of TFA (10 mM) in MeCN. (d) Time traces of absorbance at 778 nm due to the formation of Fc^{*+} for the reaction of **$Co_2L^{1,3}$-SPh** (10 μM) with O_2 (air-saturated) by Fc* (1 mM) in presence of TFA (5 - 20 mM) in MeCN.

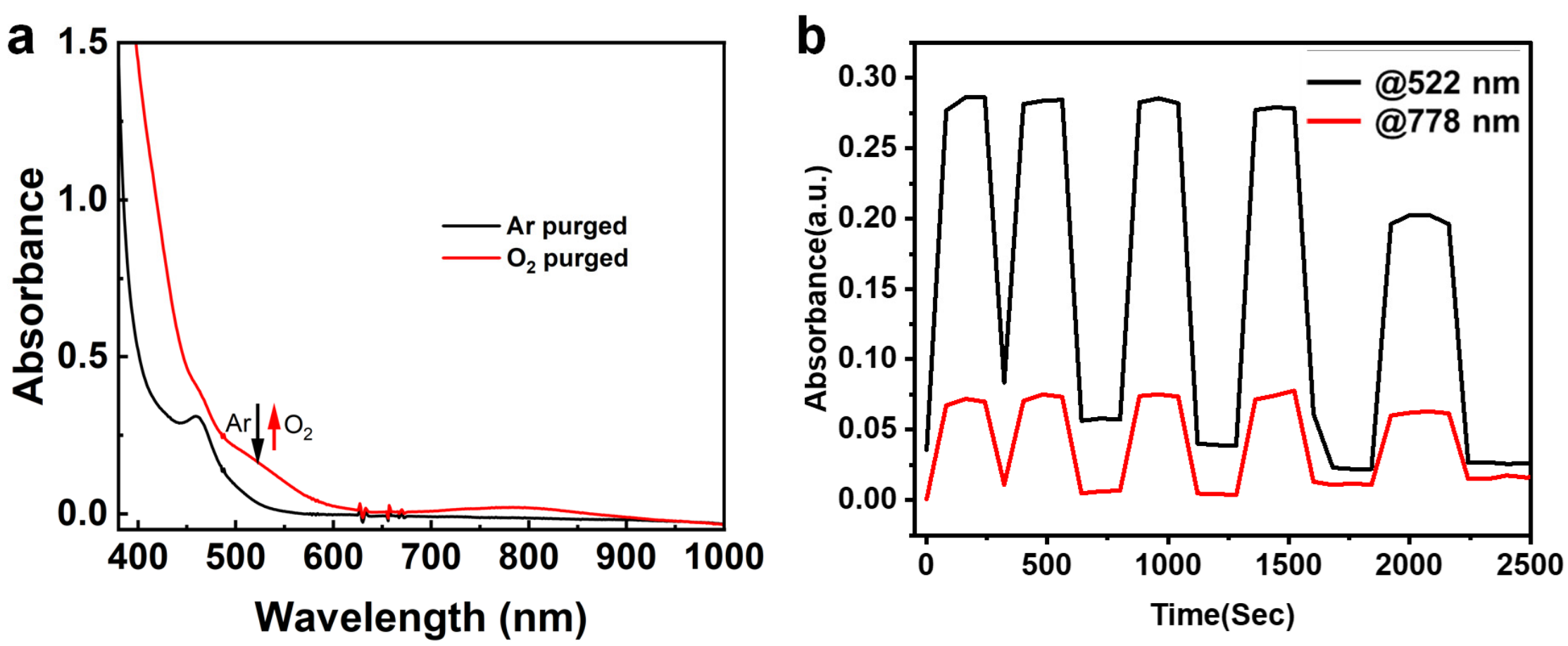

Figure S25. (a) UV–vis spectral changes observed upon O_2 purging of a MeCN solution of **$Co_2L^{1,3}$-MeCN**, followed by Ar purging at –40 ºC. (b) Time-dependent changes in the diagnostic absorption bands at 522, and 778 nm, showing reversible formation and decay of the O_2-bound intermediate.

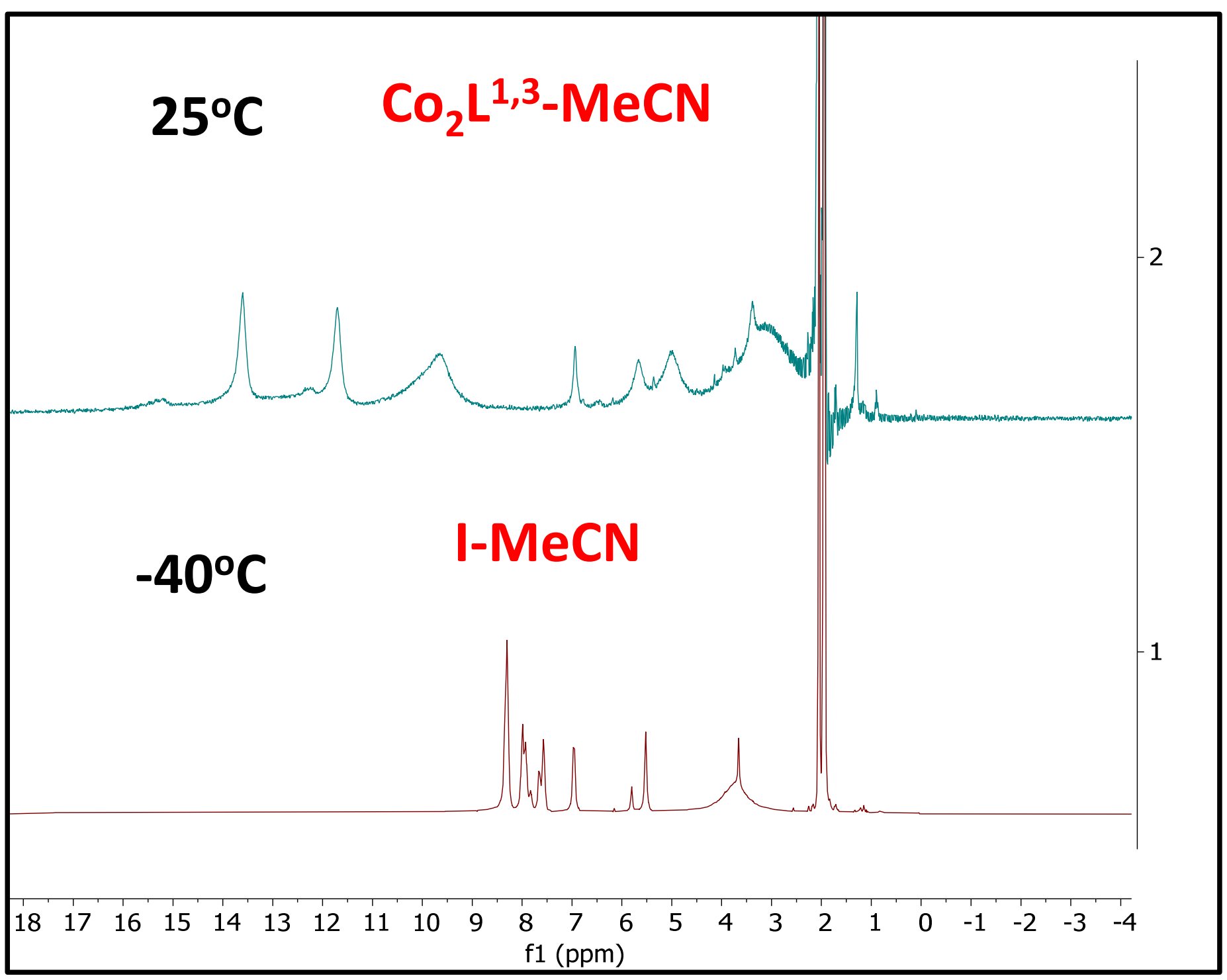

Figure S26. Changes in ^{1}H-NMR spectrum of **$Co_2L^{1,3}$-MeCN** upon O_2 purging in CD_3CN solution at 25 ºC (top) and at –40 ºC (bottom), showing thermal reversibility of the O_2-bound intermediate.

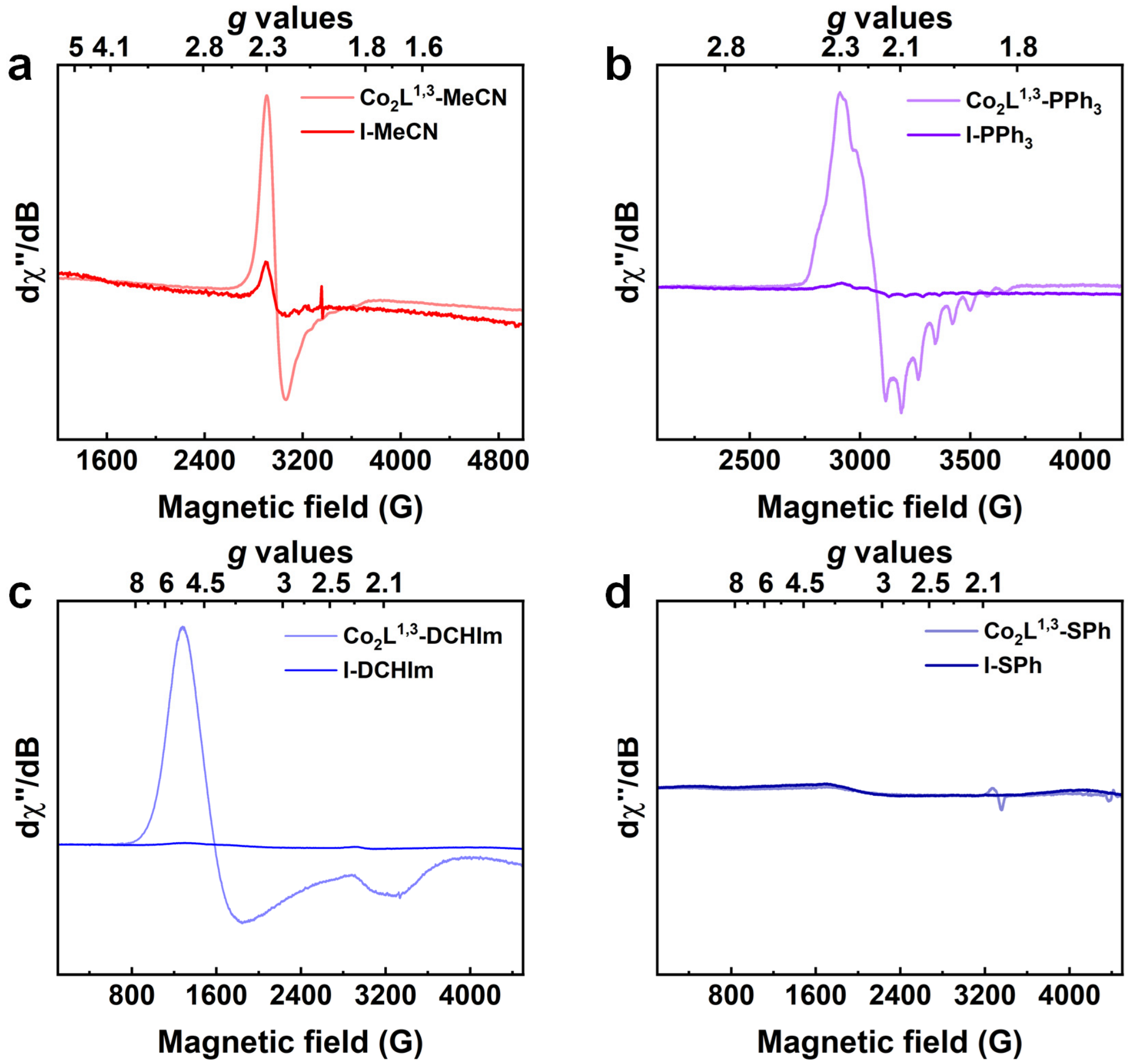

Figure S27. Comparison of the frozen-solution X-band EPR spectra of **$Co_2L^{1,3}$-X** starting complexes (light traces) and the corresponding O_2-derived intermediates **I-X** (dark traces): (a) X = MeCN, (b) X = PPh_3, (c) X = DCHIm, and (d) X = SPh. Experimental conditions: Microwave frequency ca. 9.35 GHz, microwave power: 0.016 mW, temperature: 13.5 K; modulation amplitude: 0.5 mT for $Co_2L^{1,3}$-MeCN, 0.2 mT for the other complexes; solevent: MeCN for **$Co_2L^{1,3}$-MeCN** and **I-MeCN**, butyronitrile for other complexes.

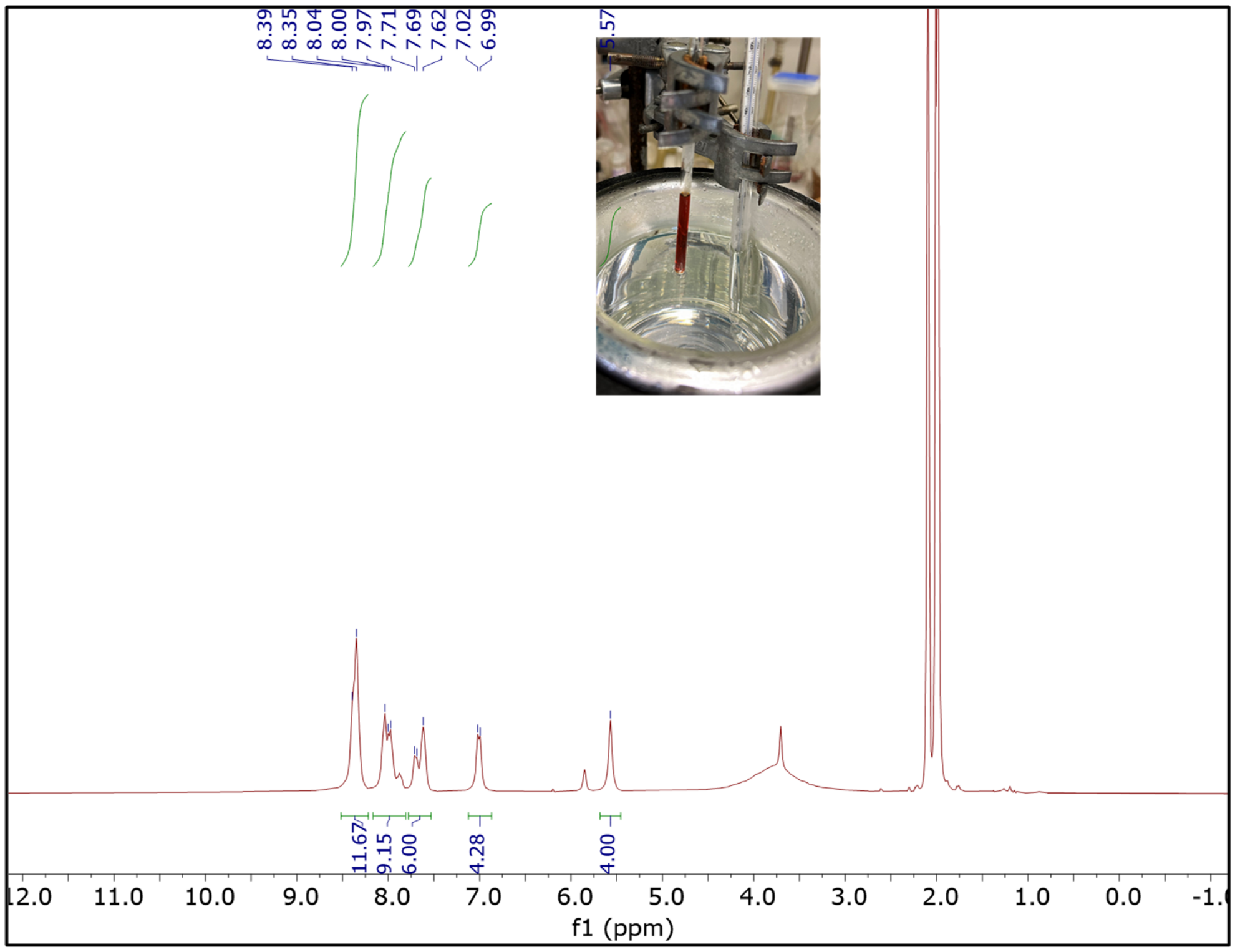

Figure S28. ^{1}H-NMR spectrum of the **I-MeCN** in CD_3CN at 233 K.

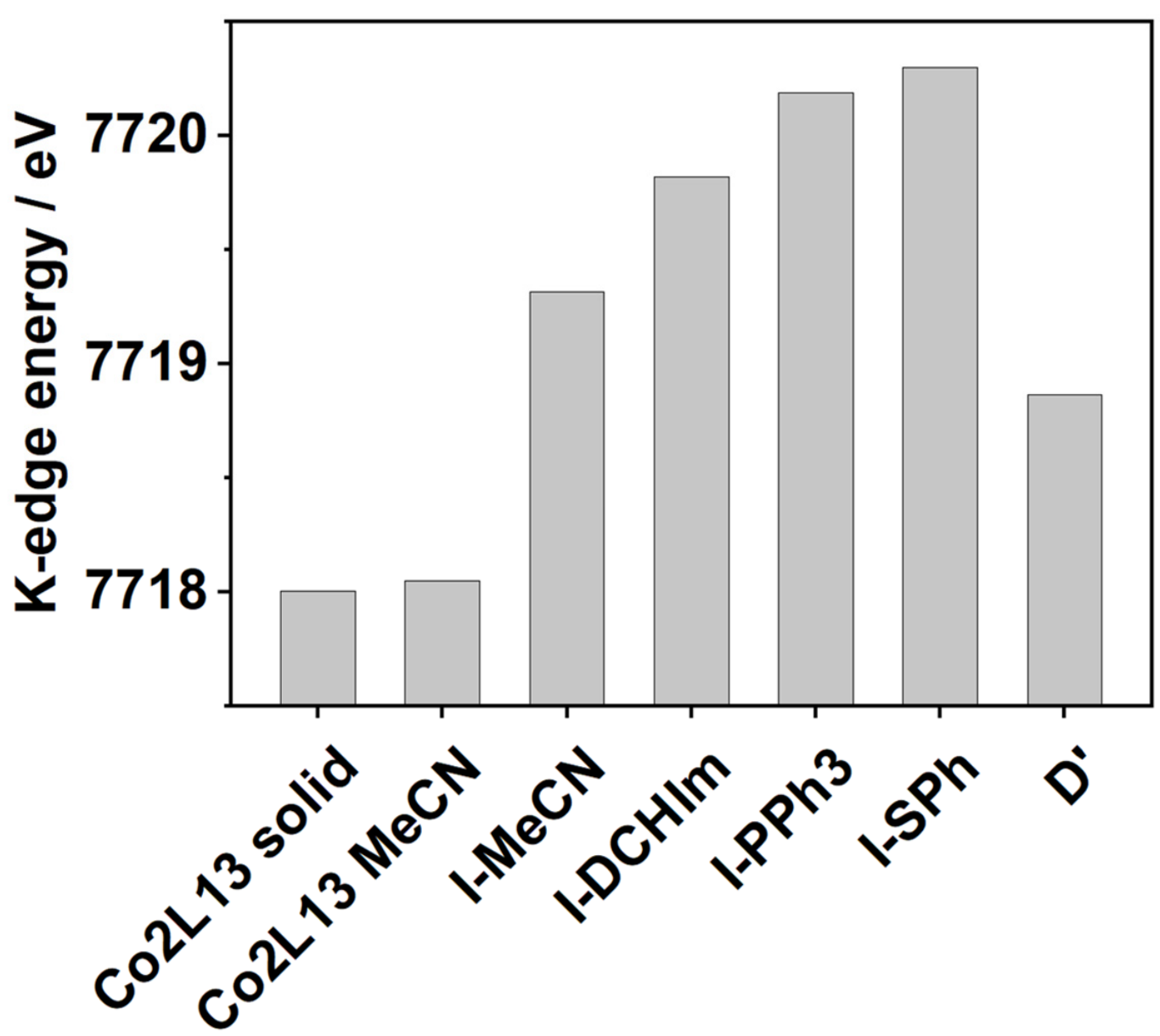

Figure S29. XAS spectra at the Co K-edge. Co K-edge energies (determined with the integral method in a 0.15-0.9 amplitude range) of the XANES spectra shown in figure 4a.

.

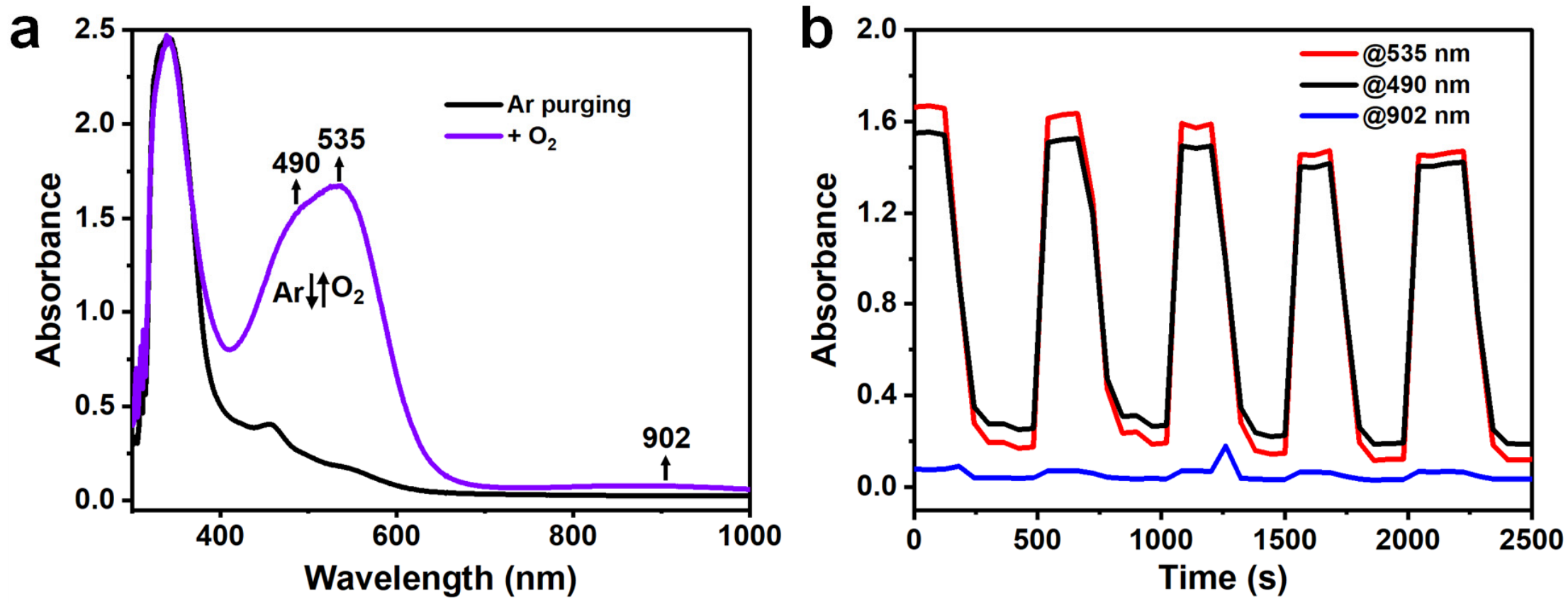

Figure S30. (a) UV–vis spectral changes observed upon O_2 purging of a MeCN solution of $\mathbf{Co_2L^{1,3}}$**-PPh**$_3$, followed by Ar purging at –40 ºC. (b) Time-dependent changes in the diagnostic absorption bands at 490, 535, and 902 nm, showing reversible formation and decay of the O_2-bound intermediate.

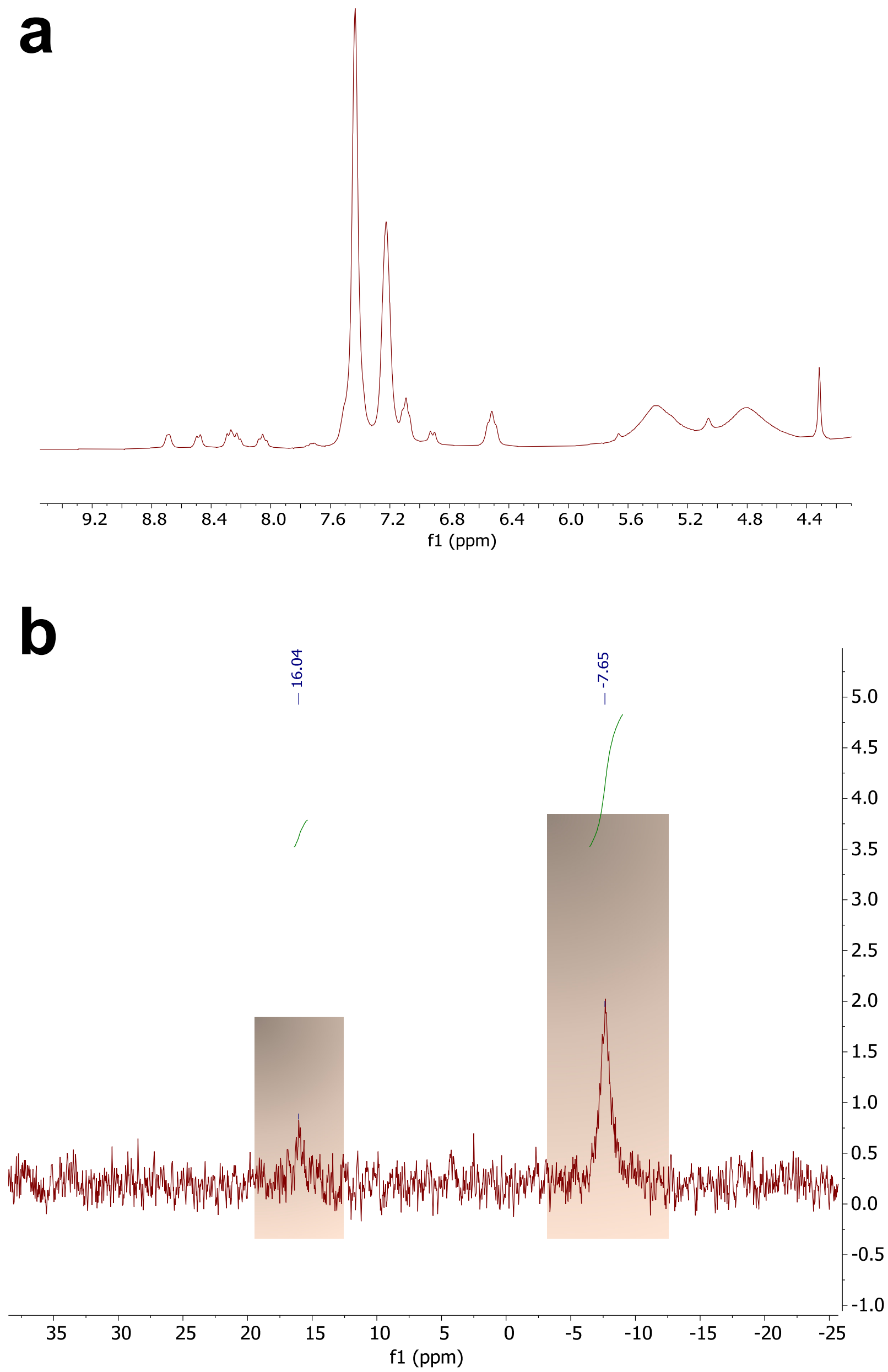

Figure S31. (a) ^{1}H-NMR and (b) ^{31}P-NMR spectrum of the **I-PPh$_3$** in CD_3CN at –40 °C.

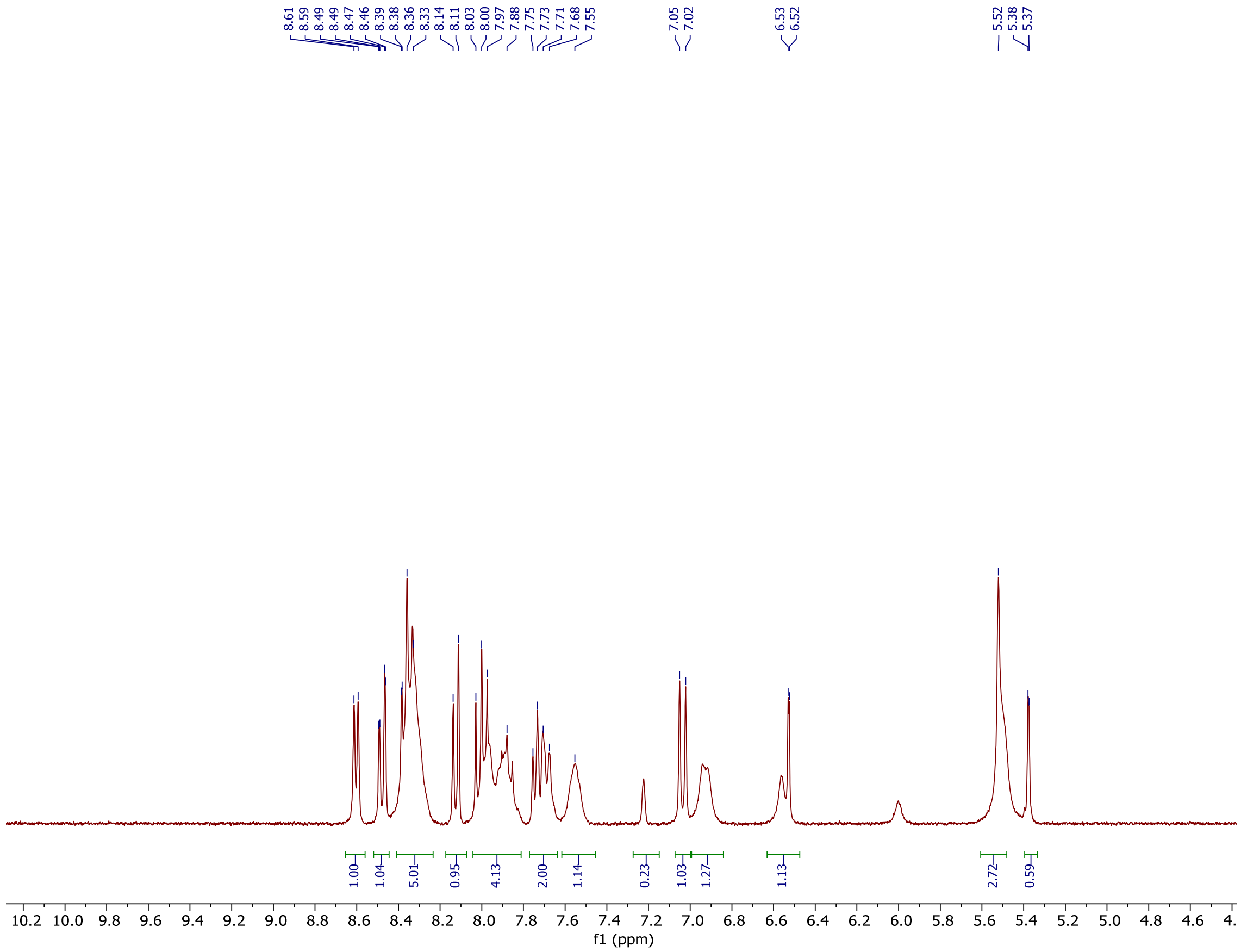

Figure S32. ^{1}H-NMR spectrum of the **I-DCHIm** in CD_3CN at 25 ºC.

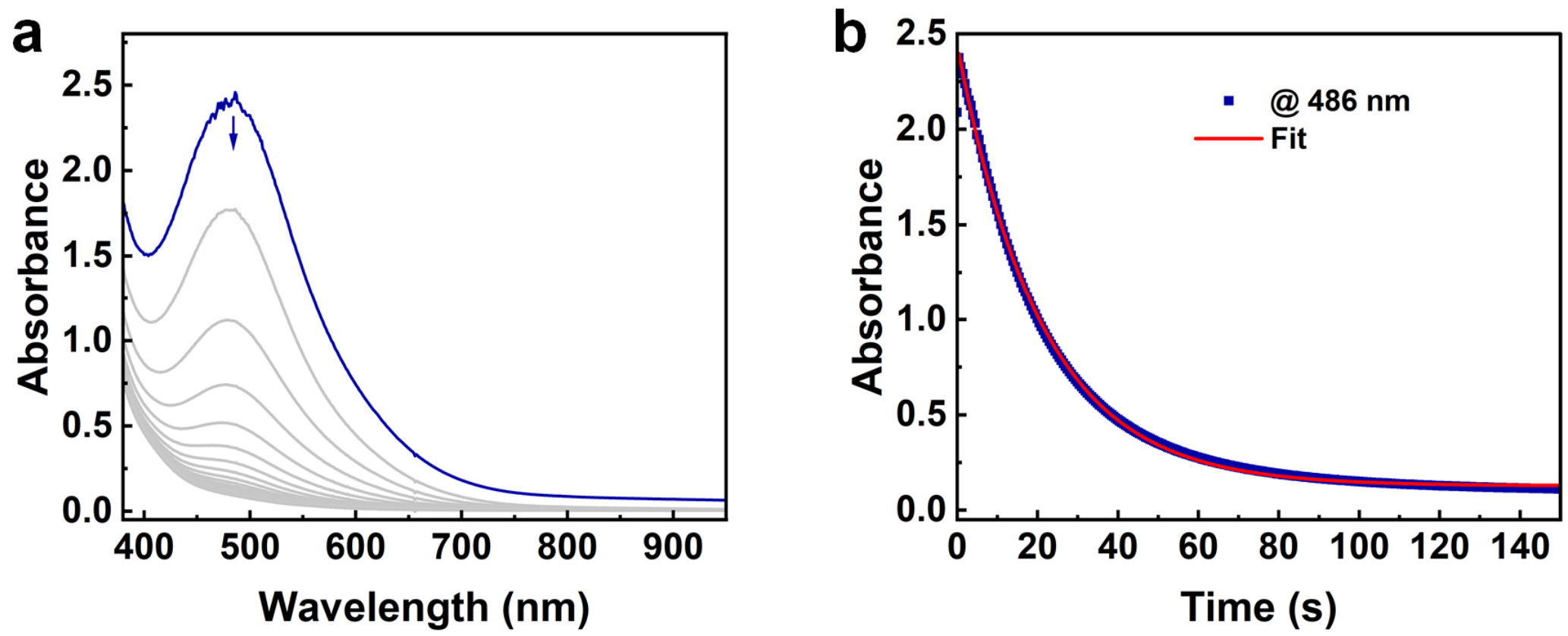

Figure S33. (a) UV-vis profile for the self-decay of **I-SPh** intermediate at r.t. in MeCN. (b) Time trace of the 486 nm (dark blue). Fitting (red) of the band gave k_{obs} value of 4.76×10^{-2} s^{-1}.

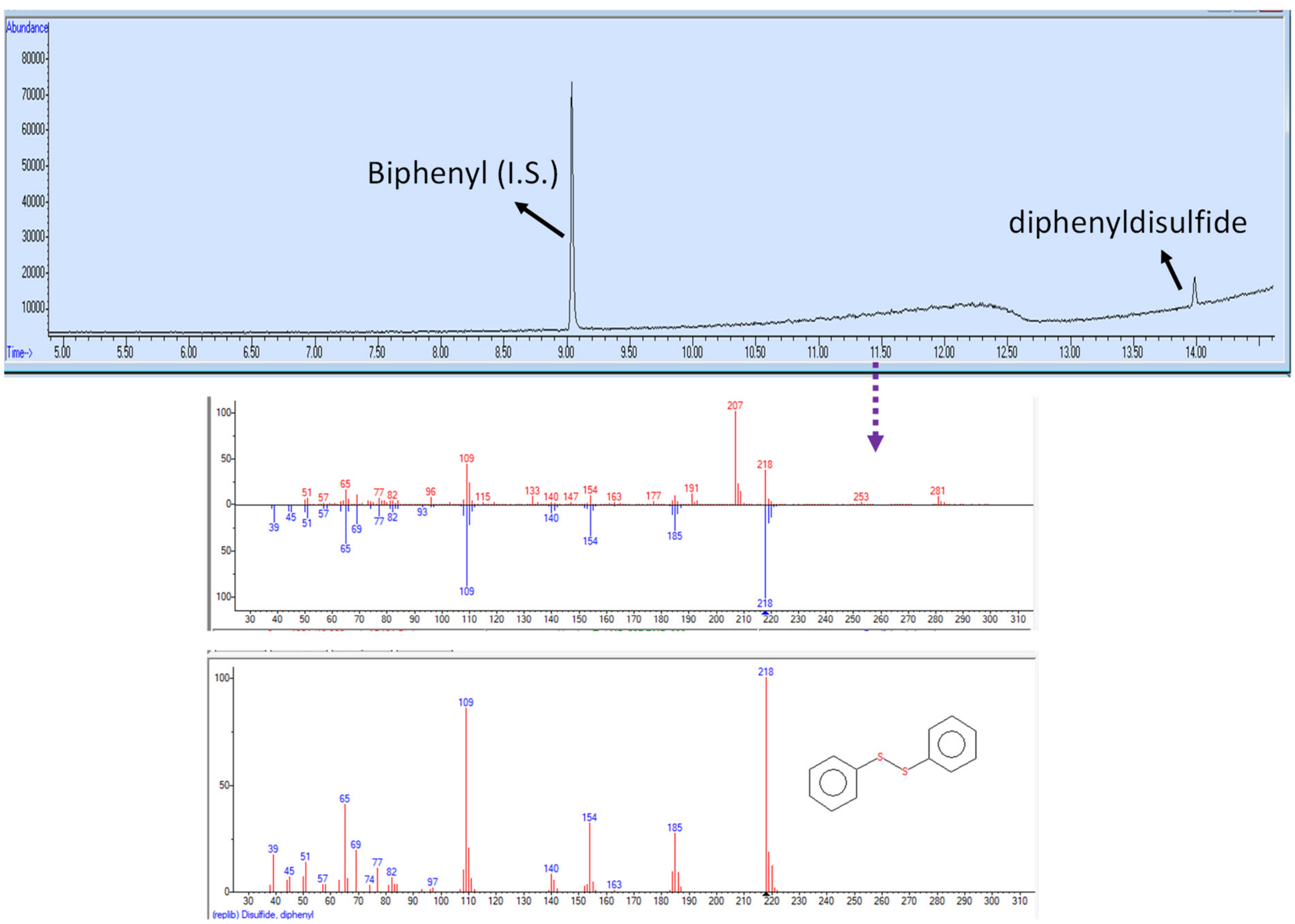

Figure S34. (Top) GC-MS analysis after the self-decay of **I-SPh** intermediate at r.t to **D´** for organic product analysis with biphenyl as internal standard (I.S.) which showed diphenyl disulfide formation. (Bottom) The mass spectrum (red) at 14 minutes compared to the bibliographical reported mass spectrum of diphenyl disulfide (blue).

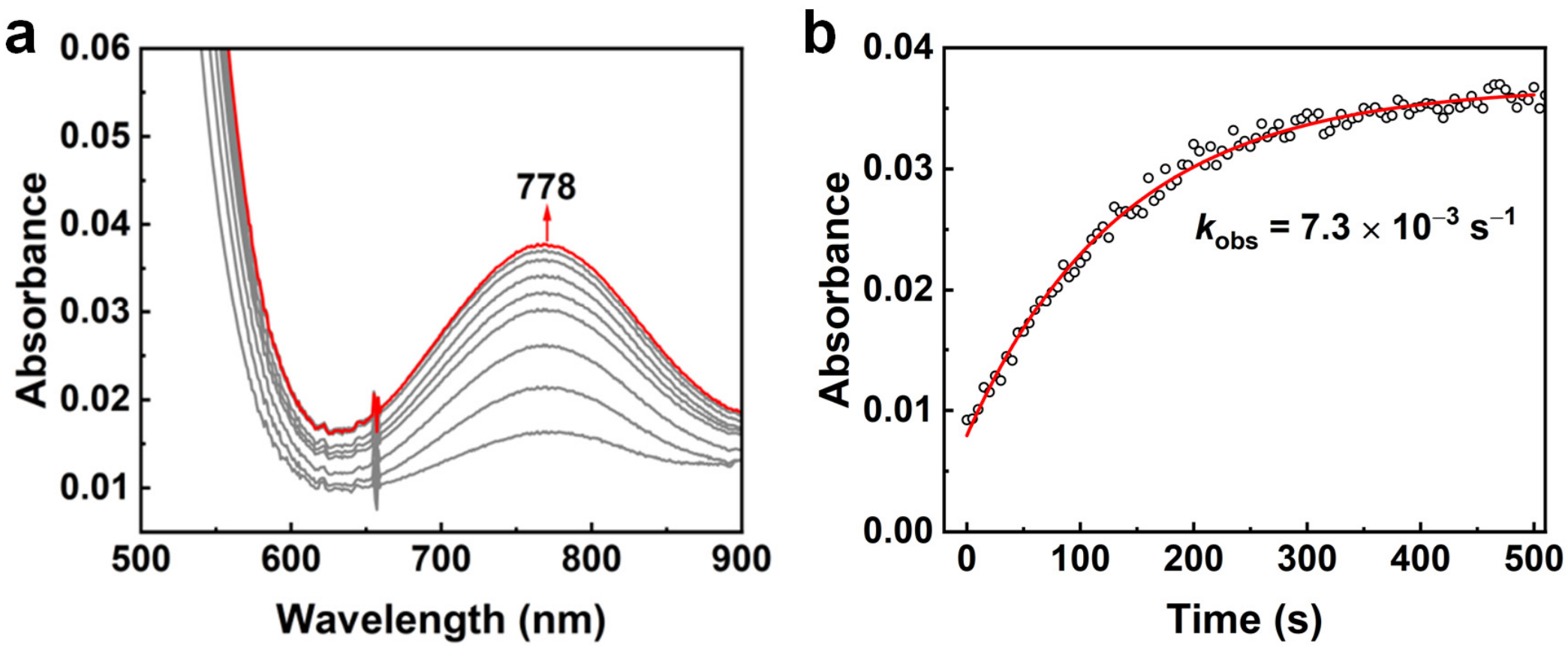

Figure S35. (a) UV-vis profile for the generation of **I-MeCN** from the reaction of **$Co_2L^{1,3}$-MeCN** (0.25 mM) and O_2 (1.7 mM) at –40 °C in MeCN. (b) Time trace of the band 778 nm for the generation of **I-MeCN** which gives a $k_{obs} = 7.3 \times 10^{-3}$ s^{-1}.

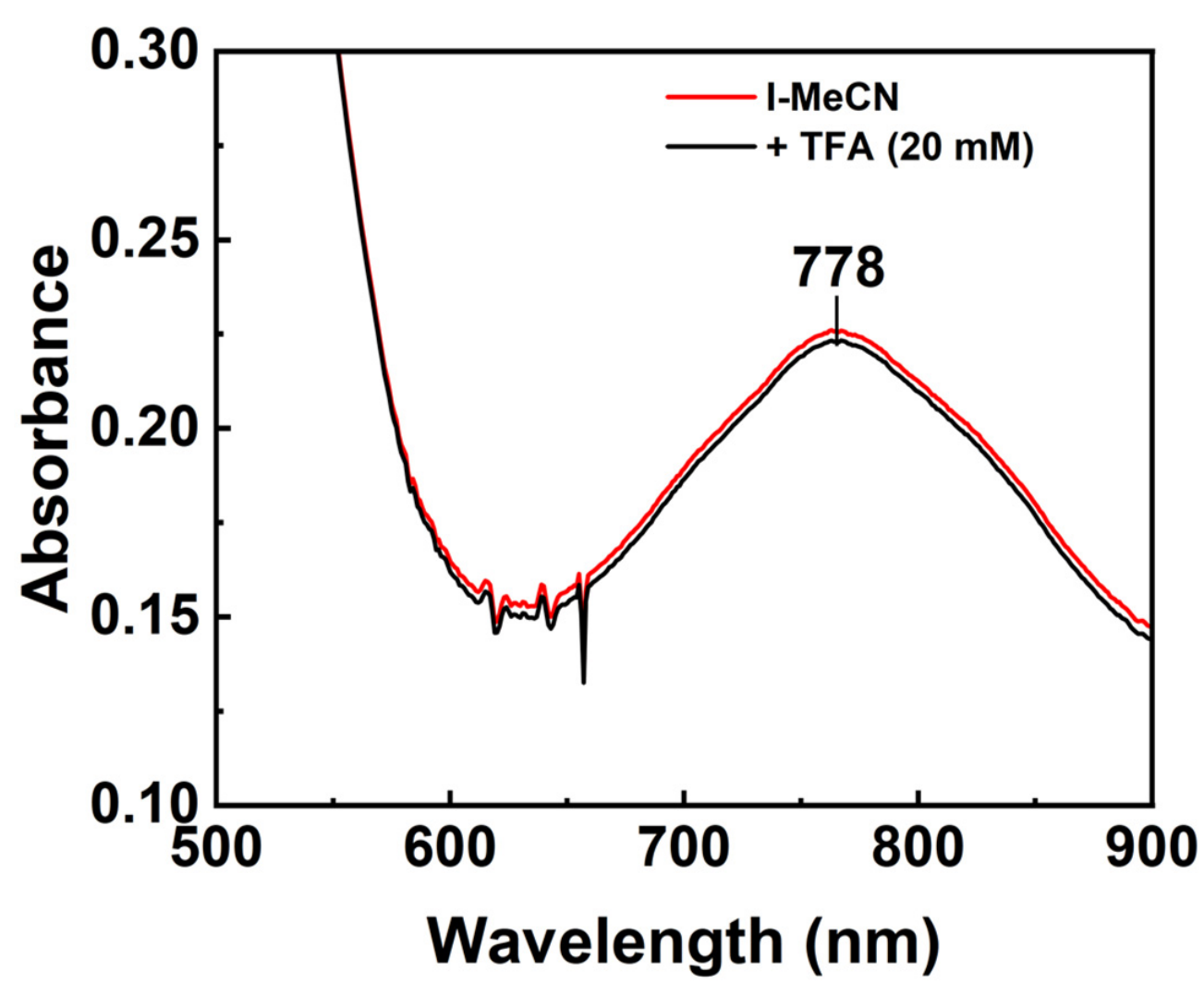

Figure S36. Reaction of **I-MeCN** with TFA (20 mM) at –40 °C in MeCN.

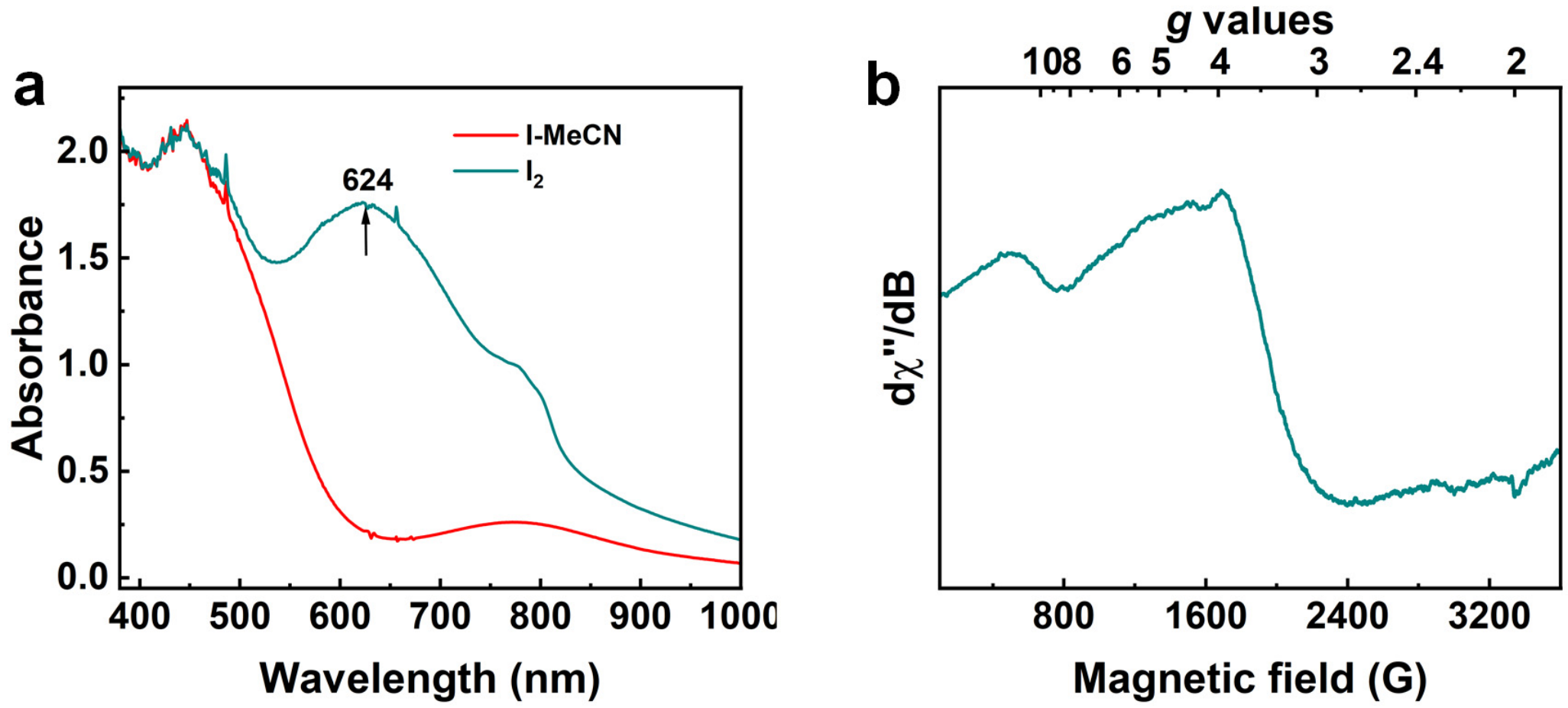

Figure S37A. (a) UV-vis, (b) EPR spectra after the reaction of **I-MeCN** (1 mM) intermediate with 1 equiv. Fc* at –40 ºC in MeCN.

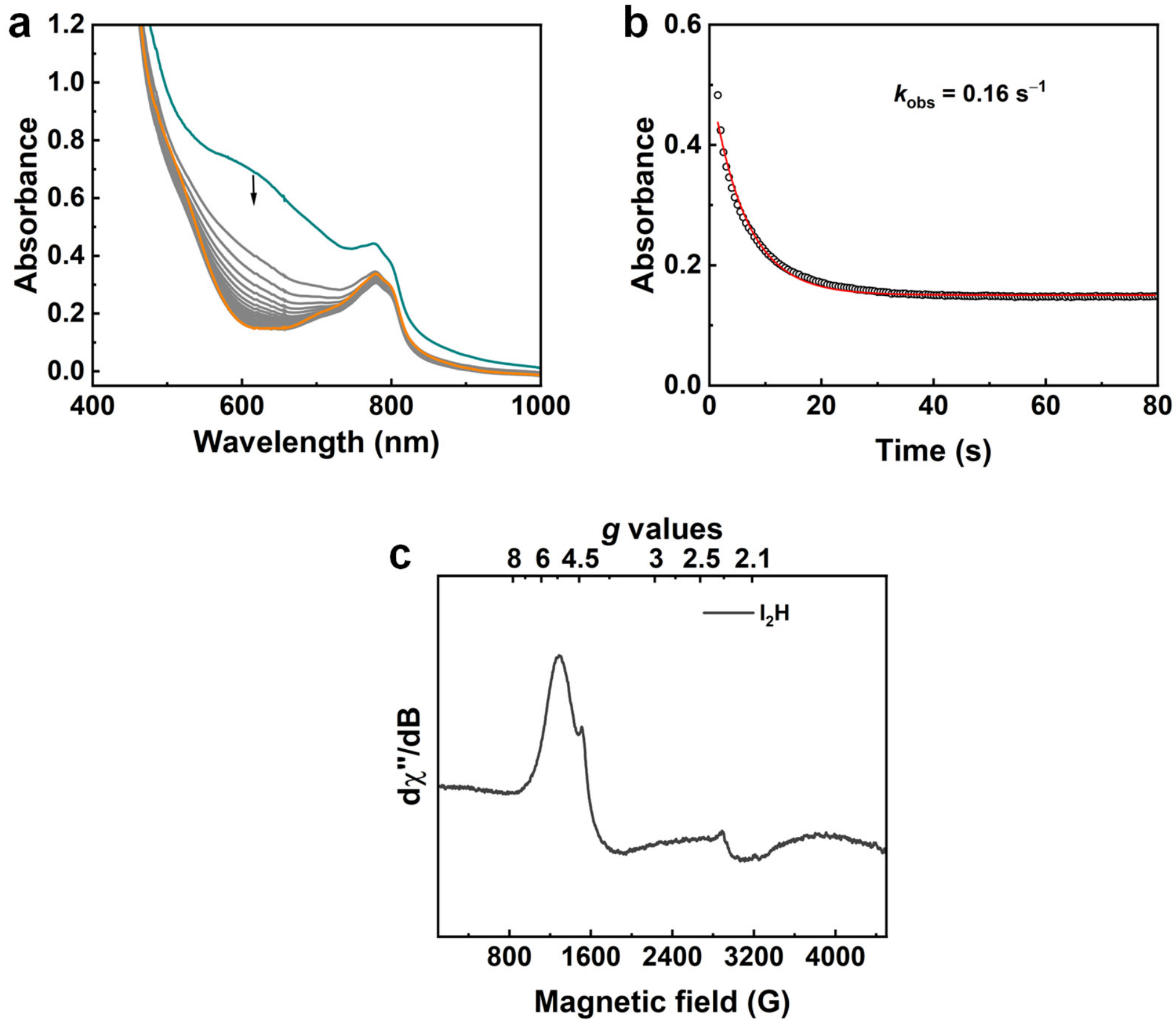

Figure S37B. (a) UV-vis profile for the generation of $\mathbf{I_2H}$ from the reaction of $\mathbf{I_2}$ (0.25 mM) and TFA (10 mM) at –40 ºC in MeCN. (b) Absorbance–time profile at 624 nm; the red line represents a single-exponential fit affording $k_{obs} = 0.16\ s^{-1}$. (c) EPR spectra of $\mathbf{I_2H}$ measured at 13 K, consistent with an $S = 3/2$ ground state.

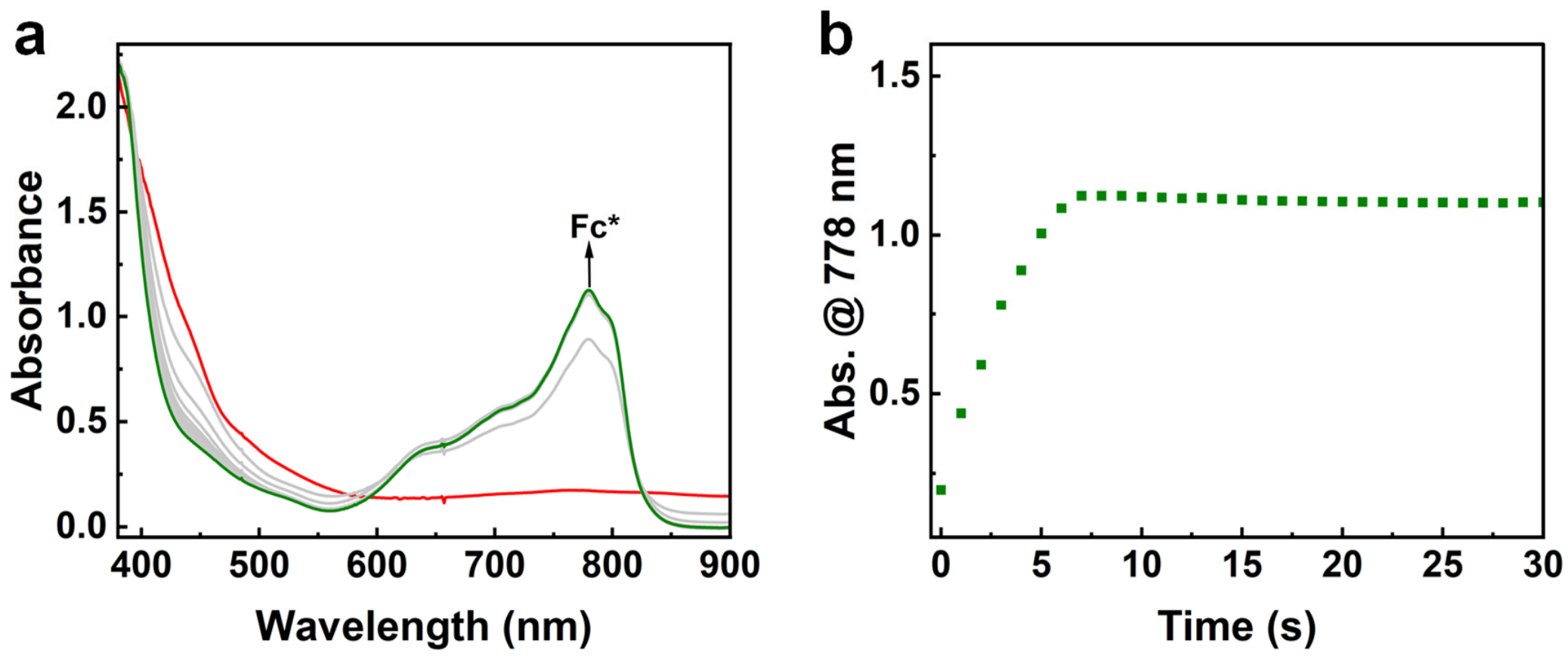

Figure S38. (a) UV-vis profile for the reaction of **I-MeCN** intermediate with Fc* (1.5 mM) in presence of TFA (10 mM) at –40 ºC in MeCN. (b) Time trace of the 778 nm band corresponding to the formation of Fc^{*+}.

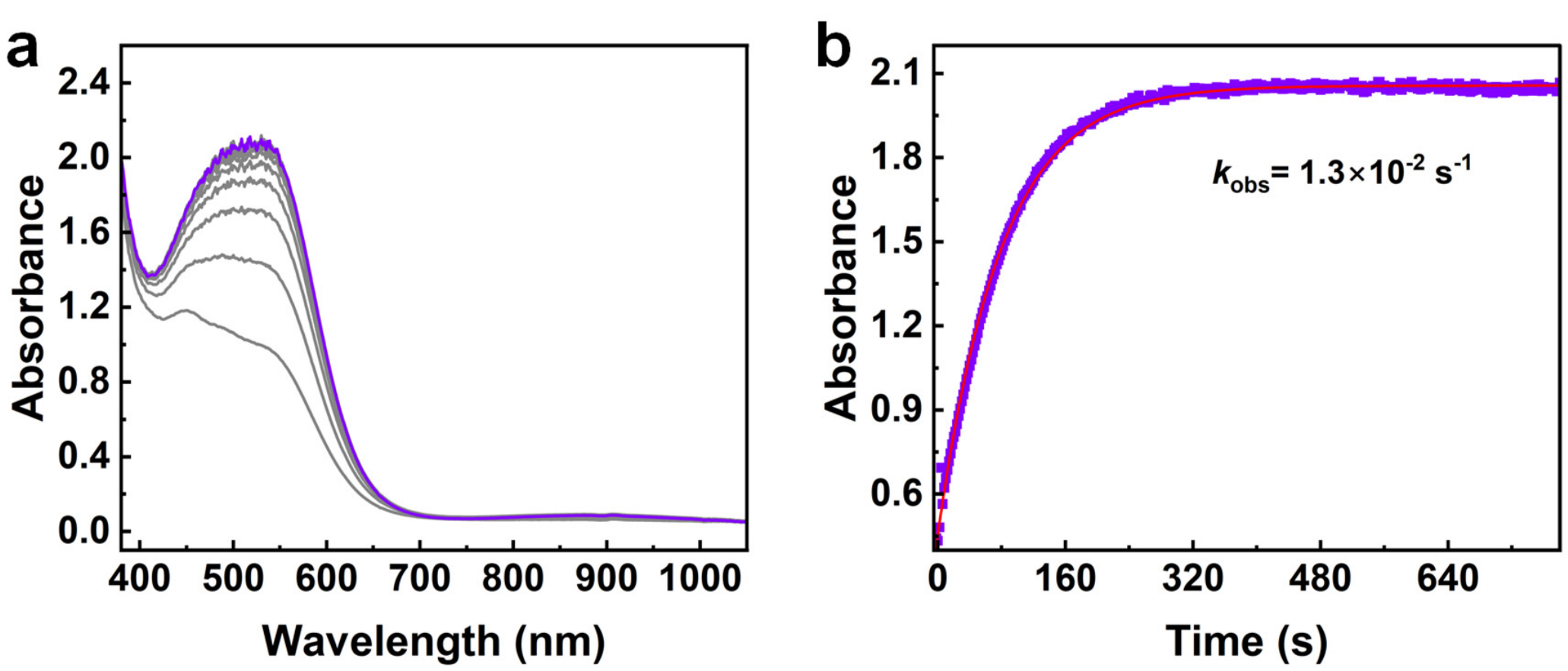

Figure 39. (a) UV-vis profile for the generation of **I-PPh$_3$** from the reaction of **Co$_2$L^{1,3}-PPh$_3$** (0.25 mM) and O_2 (1.7 mM) at –40 ºC in MeCN. (b) Time trace of the band 535 nm for the generation of **I-PPh$_3$** which gives a $k_{obs} = 1.3 \times 10^{-2}$ s^{-1}.

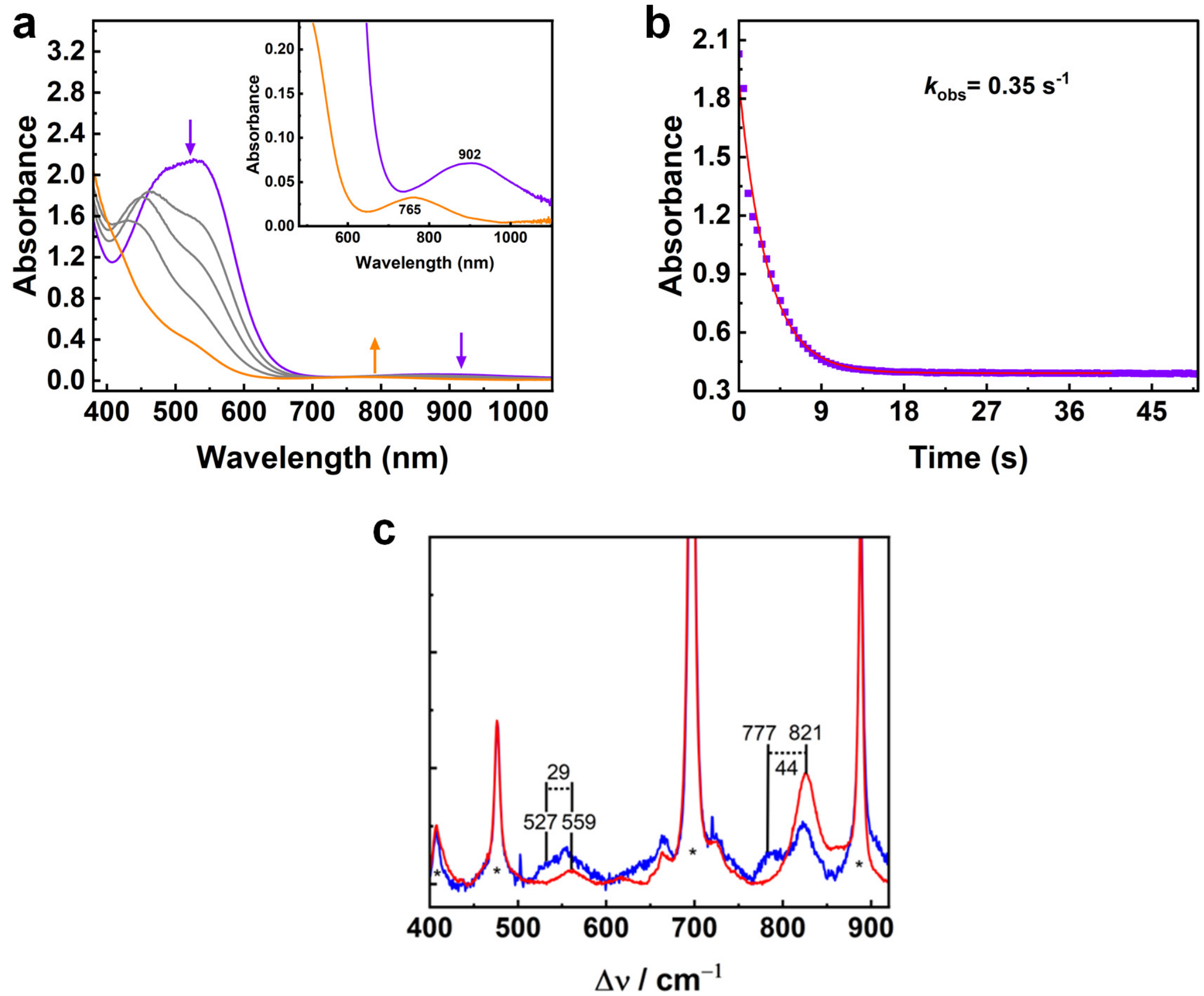

Figure S40. (a) UV-vis profile for the generation of hydroperoxido (**I_3**) intermediate from the reaction of **I-PPh_3** after reaction with TFA (10 mM) at –40 ºC in MeCN. Inset shows the low-energy absorption region highlighting diagnostic bands of the intermediates. (b) Time trace of the band 535 nm for the generation of **I_3** which gives a k_{obs} = 0.35 s^{-1}. (c) rRaman spectra of **I_3** (red) and ^{18}O-enriched analogue (blue) (λ_{ex} = 568 nm, T = –40 ºC in acetone-d$_6$). The peak marked by '∗' originates from the solvent.

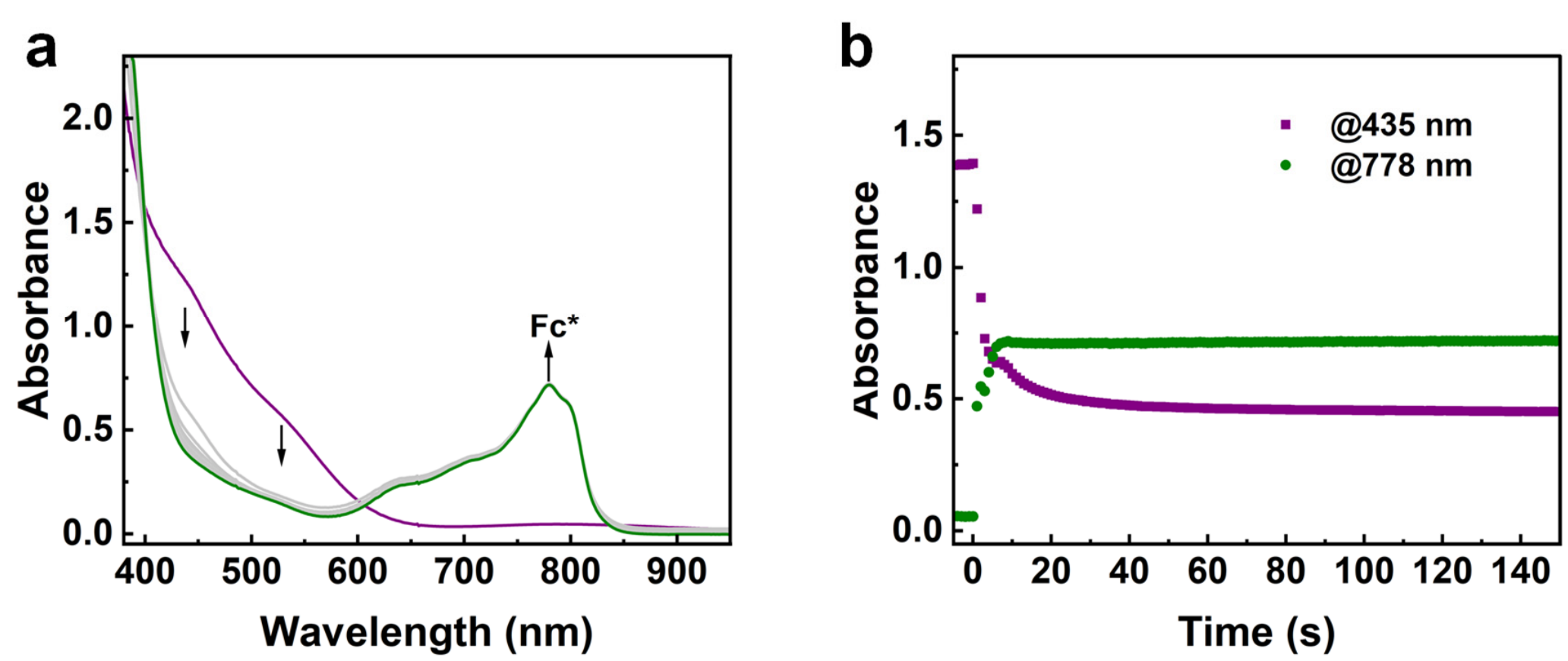

Figure S41. (a) UV-vis profile for the reaction of **I_3** intermediate with Fc* (1.5 mM) in presence of TFA (10 mM) at –40 ºC in MeCN. (b) Time trace of the 435 nm and 778 nm band corresponding to the decay of the hydroperoxido intermediate and the formation of Fc*$^+$ respectively.

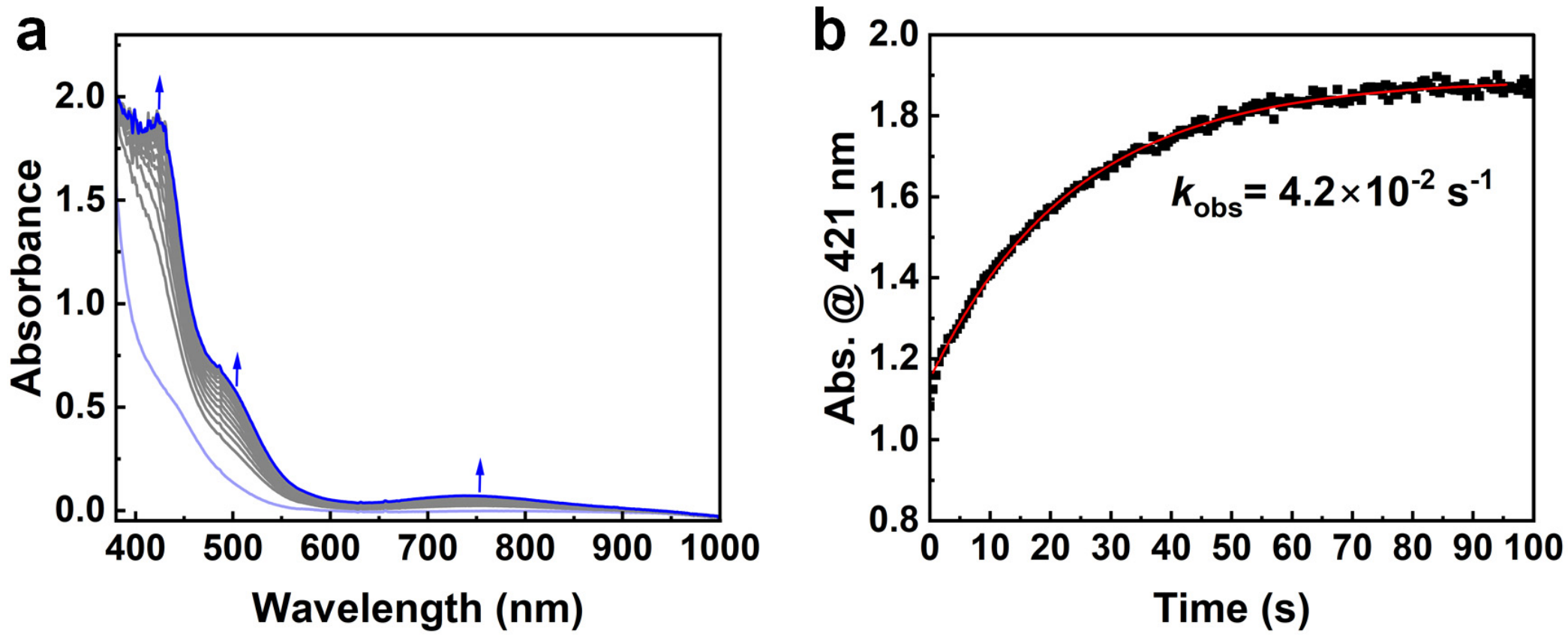

Figure 42. (a) UV-vis profile for the generation of **I-DCHIm** from the reaction of **$Co_2L^{1,3}$-DCHIm** (0.25 mM) and O_2 (0.2 mM) at −40 ºC in MeCN. (b) Time trace of the band 421 nm for the generation of **I-DCHIm** which gives a $k_{obs} = 4.3 \times 10^{-2}$ s^{-1}.

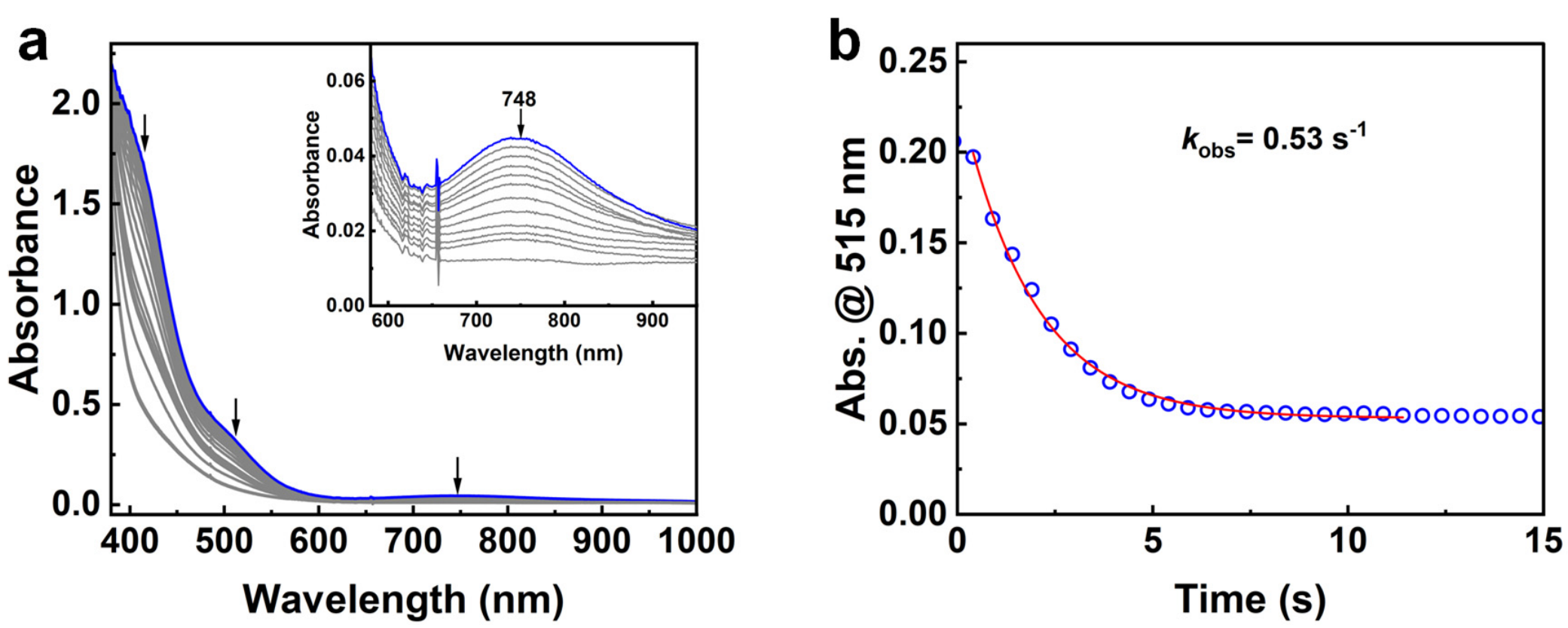

Figure S43. (a) UV-vis profile for the reaction of **I-DCHIm** after reaction with TFA (10 mM) at −40 ºC in MeCN. Inset shows the decay of the 748 nm band. (b) Time trace of the band 515 nm. Fitting (red) of the band gave k_{obs} value of 0.53 s^{-1}.

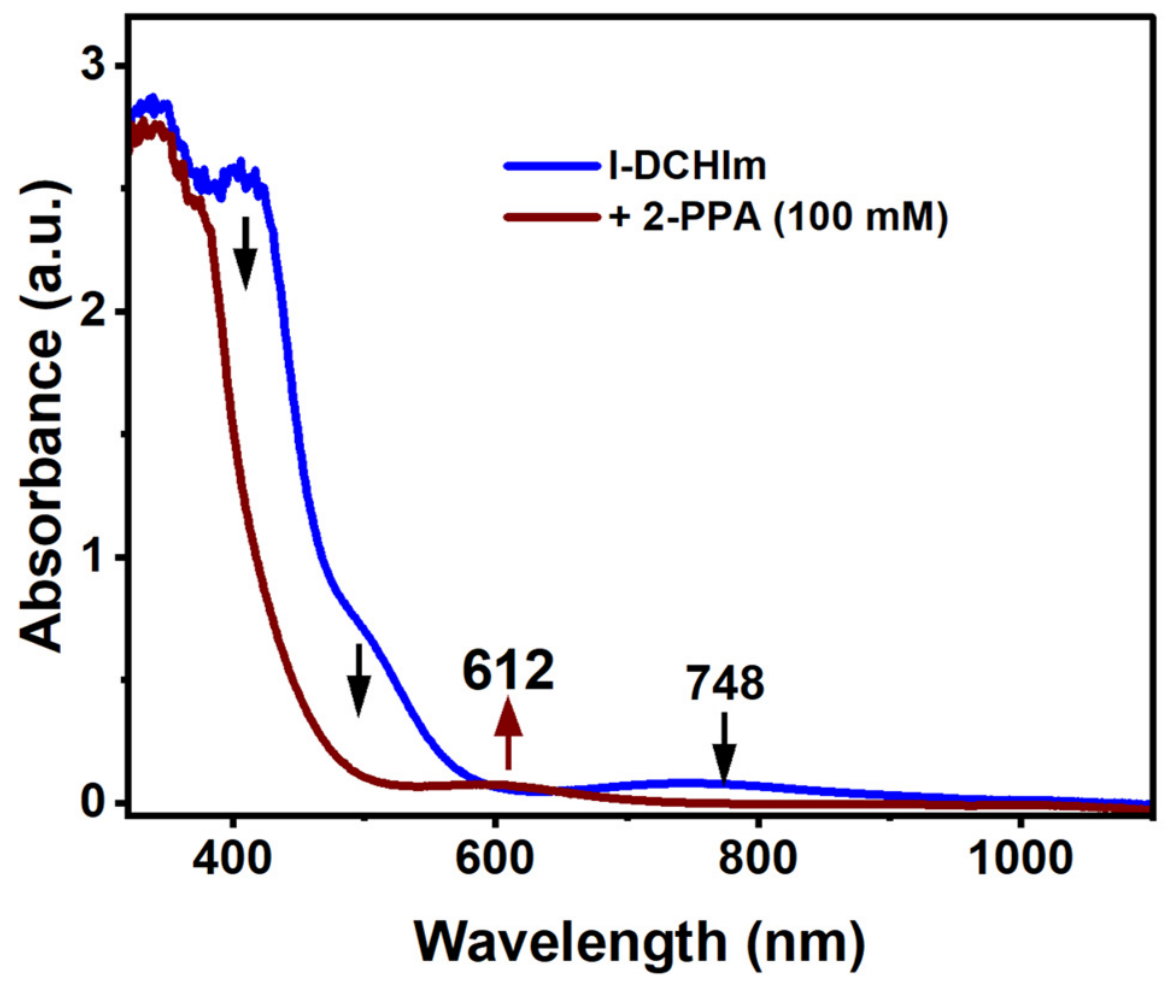

Figure S44. Change in UV-vis spectra after the reaction of **I-DCHIm** with 2-PPA (100 mM) at r.t. showing its nucleophilic character.

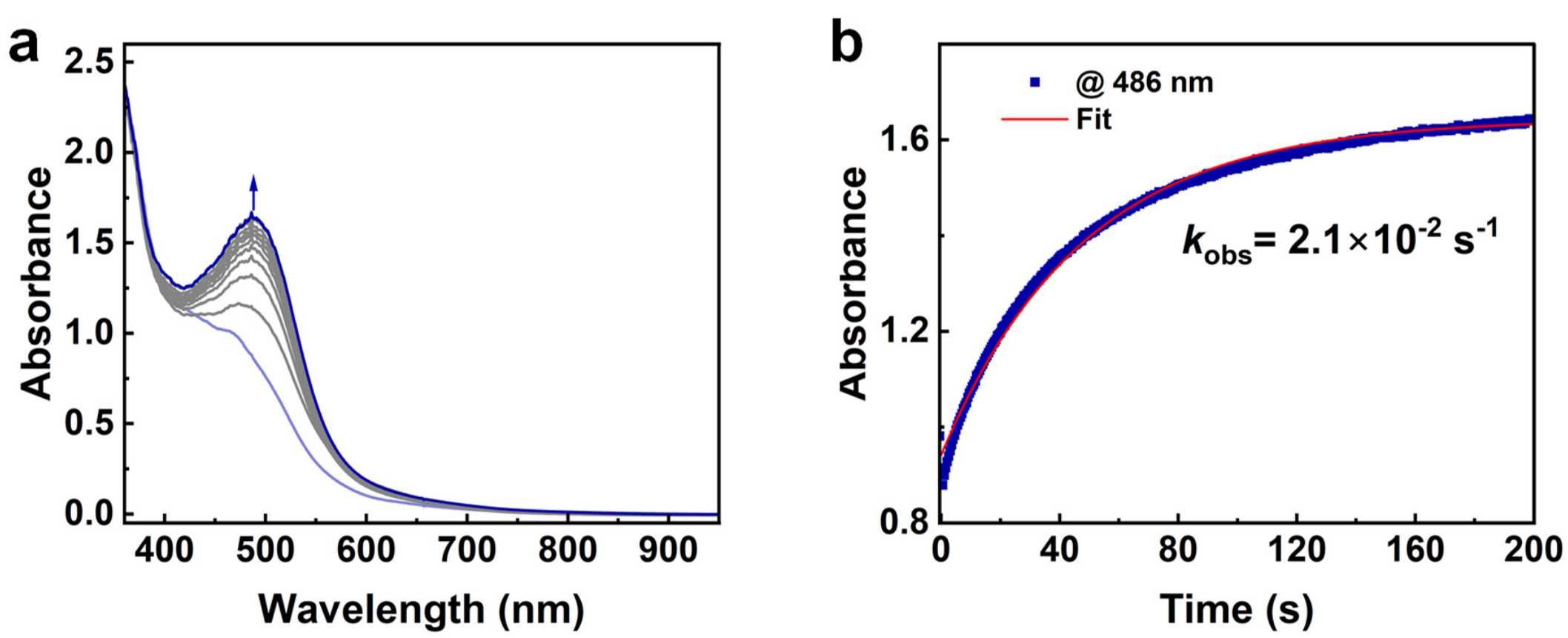

Figure S45. (a) UV-vis profile for the formation of **I-SPh** from the reaction of **$Co_2L^{1,3}$-SPh** (0.25 mM) with O_2 ([O_2] = 0.2 mM) at −40 ºC in MeCN. (b) Time trace of the 486 nm (dark blue). Fitting (red) of the band gave k_{obs} value of 2.1×10^{-2} s^{-1}.

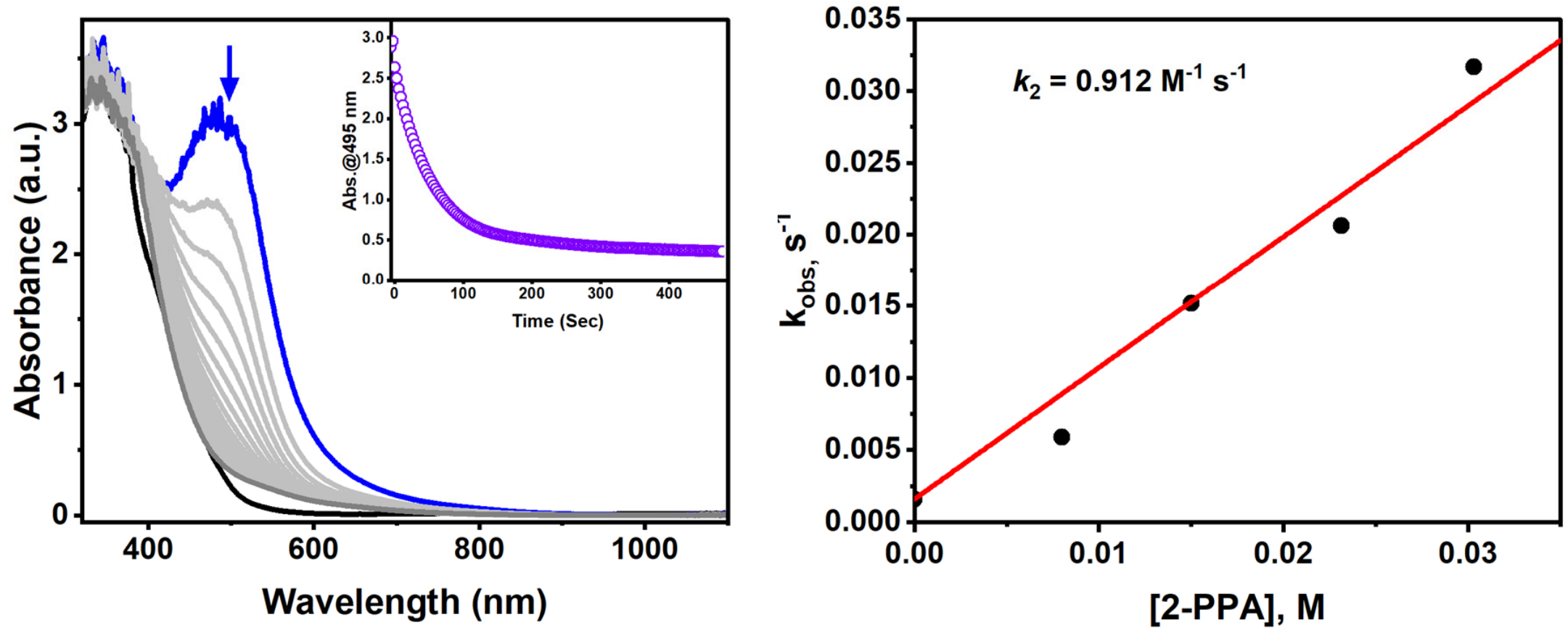

Figure S46. (a) UV-vis spectral changes observed in the reaction of **I-SPh** (blue line) and 2-PPA (15 mM) in MeCN at –10 °C. Inset shows the time course monitored at 486 nm. (B) Plots of pseudo-first-order rate constants (k_{obs}) against the concentrations of 2-PPA to determine second-order rate constant (k_2).

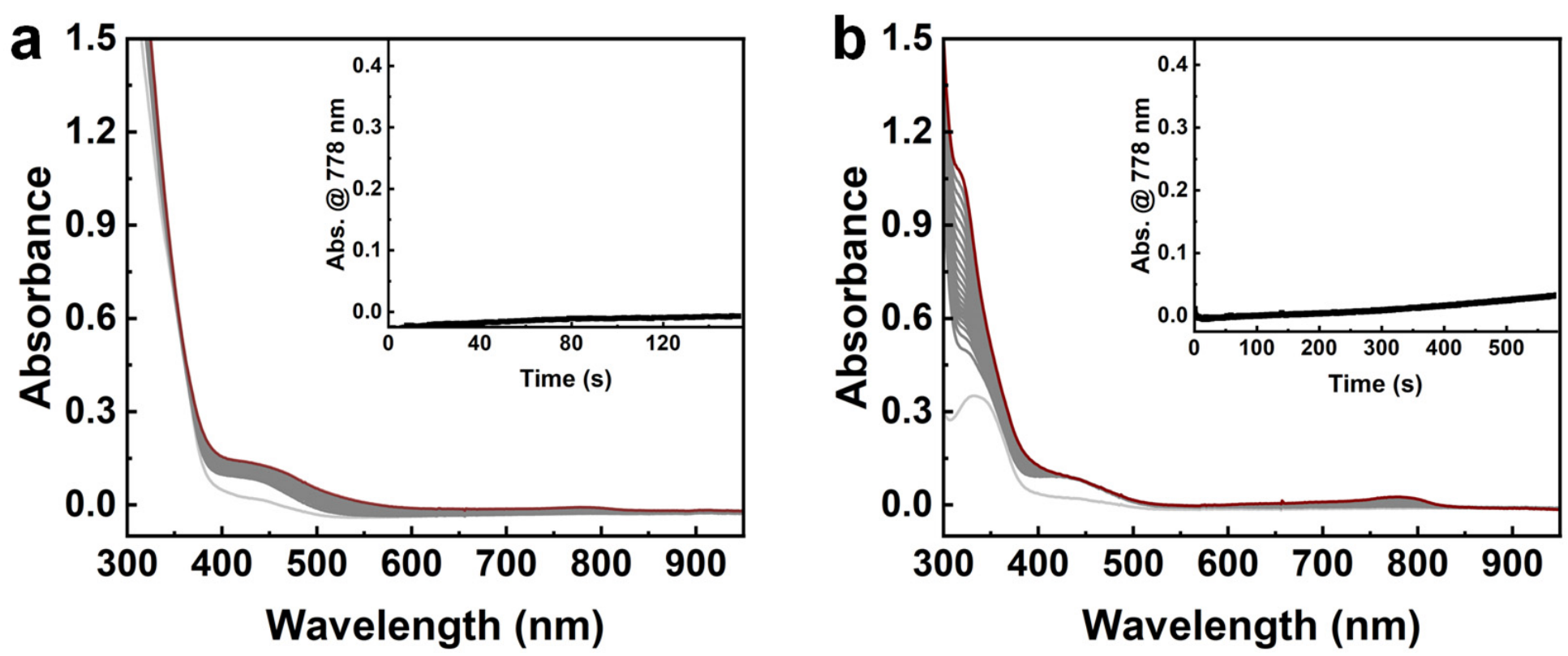

Figure S47. Time-resolved UV-vis spectra examining the reduction of PhSSPh by Fc* in MeCN at 298 K: (a) Fc* (1.0 mM), PhSSPh (1.0 mM), and TFA (10 mM); (b) Fc* (1.0 mM), PhSSPh (20 μM), **$Co_2L^{1,3}$-SPh** (10 μM), and TFA (10 mM). Insets show the corresponding absorbance changes at 778 nm, characteristic of Fc^{*+} formation. The residual formation of Fc^{*+} formation is due to trace O_2 entering during the timescale of the reaction.

5 References

(1) Jux, M.; Mebs, S.; Haumann, M.; Katz, S.; Garcia-Serres, R.; Hildebrandt, P.; Wang, Y.; Nam, W.; Ray, K. A mononuclear nonheme iron complex with higher affinity for O_2 than CO via hydrogen bonding. *Nat Commun* **2026**, *17* (1). DOI: https://doi.org/10.1038/s41467-026-72599-y.
(2) Stoll, S.; Schweiger, A. EasySpin, a comprehensive software package for spectral simulation and analysis in EPR. *Journal of magnetic resonance* **2006**, *178* (1), 42–55. DOI: https://doi.org/10.1016/j.jmr.2005.08.013.
(3) Sheldrick, G. SHELXT - Integrated space-group and crystal-structure determination. *Acta Crystallographica Section A* **2015**, *71* (1), 3–8. DOI: https://doi.org/10.1107/S2053273314026370.
(4) Dolomanov, O.; Bourhis, L.; Gildea, R.; Howard, J.; Puschmann, H. OLEX2: A complete structure solution, refinement and analysis program. *J. Appl. Cryst. J. Appl. Cryst* **2009**, *42*, 339–341. DOI: https://doi.org/10.1107/S0021889808042726.
(5) Sheldrick, G. Crystal structure refinement with SHELXL. *Acta Crystallographica Section C* **2015**, *71* (1), 3–8. DOI: https://doi.org/10.1107/S2053229614024218.
(6) (a) Devi, T.; Dutta, K.; Deutscher, J.; Mebs, S.; Kuhlmann, U.; Haumann, M.; Cula, B.; Dau, H.; Hildebrandt, P.; Ray, K. A high-spin alkylperoxo–iron (III) complex with cis-anionic ligands: implications for the superoxide reductase mechanism. *Chemical Science* **2024**, *15* (2), 528–533. (b) Kass, D.; Yao, S.; Krause, K. B.; Corona, T.; Richter, L.; Braun, T.; Mebs, S.; Haumann, M.; Dau, H.; Lohmiller, T. Spectroscopic Properties of a Biologically Relevant [Fe2 (μ-O) 2] Diamond Core Motif with a Short Iron-Iron Distance. *Angewandte Chemie International Edition* **2023**, *62* (10), e202209437.
(7) Dau, H.; Liebisch, P.; Haumann, M. X-ray absorption spectroscopy to analyze nuclear geometry and electronic structure of biological metal centers—potential and questions examined with special focus on the tetra-nuclear manganese complex of oxygenic photosynthesis. *Analytical and bioanalytical chemistry* **2003**, *376* (5), 562–583.
(8) Rehr, J. J.; Kas, J. J.; Vila, F. D.; Prange, M. P.; Jorissen, K. Parameter-free calculations of X-ray spectra with FEFF9. *Physical Chemistry Chemical Physics* **2010**, *12* (21), 5503–5513.
(9) Bera, A.; Bimmermann, S.; Gerschel, P.; Barman, D. J.; Gerndt, L.; Lohmiller, T.; Abdiaziz, K.; Schnegg, A.; Orio, M.; Hetterscheid, D. G. H.; et al. Mechanistic Promiscuity in Cobalt-Mediated CO_2 Reduction Reaction: One- Versus Two-Electron Reduction Process. *Angew Chem Int Ed Engl* **2025**, *64* (31), e202503705. DOI: https://doi.org/10.1002/anie.202503705.
(10) Hicks, M.; Gebicki, J. M. A spectrophotometric method for the determination of lipid hydroperoxides. *Analytical Biochemistry* **1979**, *99* (2), 249–253. DOI: https://doi.org/10.1016/S0003-2697(79)80003-2.
(11) (a) Chang, C. J.; Deng, Y.; Shi, C.; Chang, C. K.; Anson, F. C.; Nocera, D. G. Electrocatalytic four-electron reduction of oxygen to water by a highly flexible cofacial cobalt bisporphyrin. *Chemical Communications* **2000**, *36* (15), 1355–1356. DOI: https://doi.org/10.1039/B001620I. (b) Chang, C. J.; Loh, Z.-H.; Shi, C.; Anson, F. C.; Nocera, D. G. Targeted Proton Delivery in the Catalyzed Reduction of Oxygen to Water by Bimetallic Pacman Porphyrins. *J Am Chem Soc* **2004**, *126* (32), 10013–10020. DOI: https://doi.org/10.1021/ja049115j. (c) Fukuzumi, S.; Okamoto, K.; Gros, C. P.; Guilard, R. Mechanism of Four-Electron Reduction of Dioxygen to Water by Ferrocene Derivatives in the Presence of Perchloric Acid in Benzonitrile, Catalyzed by Cofacial Dicobalt Porphyrins. *J Am Chem Soc* **2004**, *126* (33), 10441–10449. DOI: https://doi.org/10.1021/ja048403c.
(12) (a) Kadish, K. M.; Frémond, L.; Ou, Z.; Shao, J.; Shi, C.; Anson, F. C.; Burdet, F.; Gros, C. P.; Barbe, J.-M.; Guilard, R. Cobalt(III) Corroles as Electrocatalysts for the Reduction of Dioxygen: Reactivity of a Monocorrole, Biscorroles, and Porphyrin−Corrole Dyads. *J Am Chem Soc* **2005**, *127* (15), 5625–5631. DOI: https://doi.org/10.1021/ja0501060. (b) Volpe, M.; Hartnett, H.; Leeland, J. W.; Wills, K.; Ogunshun, M.; Duncombe, B. J.; Wilson, C.; Blake, A. J.; McMaster, J.; Love, J. B. Binuclear Cobalt Complexes of Schiff-Base Calixpyrroles and Their Roles in the Catalytic Reduction of Dioxygen. *Inorganic Chemistry* **2009**, *48* (12), 5195–5207. DOI: https://doi.org/10.1021/ic9001175.
(13) Givaja, G.; Volpe, M.; Edwards, M. A.; Blake, A. J.; Wilson, C.; Schröder, M.; Love, J. B. Dioxygen Reduction at Dicobalt Complexes of a Schiff Base Calixpyrrole Ligand. *Angew Chem Int Ed Engl* **2007**, *46* (4), 584–586. DOI: https://doi.org/10.1002/anie.200603201.

(14) Askarizadeh, E.; Yaghoob, S. B.; Boghaei, D. M.; Slawin, A. M. Z.; Love, J. B. Tailoring dicobalt Pacman complexes of Schiff-base calixpyrroles towards dioxygen reduction catalysis. *Chemical Communications* **2010**, *46* (5), 710–712. DOI: https://doi.org/10.1039/b923189g.
(15) Oldacre, A. N.; Friedman, A. E.; Cook, T. R. A Self-Assembled Cofacial Cobalt Porphyrin Prism for Oxygen Reduction Catalysis. *J Am Chem Soc* **2017**, *139* (4), 1424–1427. DOI: https://doi.org/10.1021/jacs.6b12404.
(16) Crawley, M. R.; Zhang, D.; Oldacre, A. N.; Beavers, C. M.; Friedman, A. E.; Cook, T. R. Tuning the Reactivity of Cofacial Porphyrin Prisms for Oxygen Reduction Using Modular Building Blocks. *J Am Chem Soc* **2020**, *143* (2), 1098–1106. DOI: https://doi.org/10.1021/jacs.0c11895.
(17) Liu, Y.; Zhou, G.; Zhang, Z.; Lei, H.; Yao, Z.; Li, J.; Lin, J.; Cao, R. Significantly improved electrocatalytic oxygen reduction by an asymmetrical Pacman dinuclear cobalt(II) porphyrin–porphyrin dyad. *Chemical Science* **2020**, *11* (1), 87–96. DOI: https://doi.org/10.1039/c9sc05041h.
(18) Zhang, D.; Crawley, M. R.; Oldacre, A. N.; Kyle, L. J.; MacMillan, S. N.; Cook, T. R. Lowering the Symmetry of Cofacial Porphyrin Prisms for Selective Oxygen Reduction Electrocatalysis. *Inorganic Chemistry* **2022**, *62* (5), 1766–1775. DOI: https://doi.org/10.1021/acs.inorgchem.2c01109.
(19) Zhang, D.; Snider, R. L.; Crawley, M. R.; Fang, M.; Sanchez-Lievanos, K. R.; Ang, S.; Cook, T. R. Gram-Scale, One-Pot Synthesis of a Cofacial Porphyrin Bridged by Ortho-xylene as a Scaffold for Dinuclear Architectures. *Inorganic Chemistry* **2024**, *63* (47), 22532–22541. DOI: https://doi.org/10.1021/acs.inorgchem.4c03958.
